# Assessment of Double-Hybrid Functionals for the Vertical Excitation Energies of Porphyrins

**DOI:** 10.3390/molecules31173027

**Published:** 2026-08-28

**Authors:** Wissam Helal, Rahma Sarayra, Ahmad S. Barham, Ali Elrashidi

**Affiliations:** 1 Department of Chemistry, School of Science, The University of Jordan, Amman 11942, Jordan; a.barham@ju.edu.jo; 2 International Independent Schools, Khalda Branch, P.O. Box 499, Amman 11732, Jordan; rahmaosarayra@gmail.com; 3 Electrical Engineering Department, College of Engineering, University of Business and Technology, Jeddah 23435, Saudi Arabia; a.elrashidi@ubt.edu.sa; 4 Engineering Mathematics Department, Faculty of Engineering, Alexandria University, Alexandria 21544, Egypt

**Keywords:** porphyrins, TD-DFT, double-hybrid functionals, spin-component-scaled, vertical excitation

## Abstract

The vertical excitation energies of 13 porphyrins and metalloporphyrins were benchmarked using 28 double-hybrid (DH) density functionals within the full TD-DFT framework, in conjunction with the def2-TZVP basis set and CPCM solvation model. The assessed functionals comprise global double hybrids (GDHs), range-separated double hybrids (RSDHs), and their spin-component-/spin-opposite-scaled variants (SCS/SOS-GDHs and SCS/SOS-RSDHs). Performance was evaluated for the B, Q1, and Q3 bands by comparison with experimental absorption maxima. B2PLYP provides one of the most balanced overall agreements, with mean absolute errors of approximately 0.03, 0.06, and 0.07 eV for the B, Q1, and Q3 bands, respectively. B2GPPLYP, ωB88PP86, and ωPBEPP86 also yield MAEs below 0.1 eV for all three bands. In contrast, several SCS/SOS-RSDH functionals perform very well for the B band but show pronounced systematic underestimation for the Q bands, revealing substantial band dependence. Comparison with published multireference vertical excitation energies further shows that close agreement with experimental absorption maxima does not necessarily imply equally close agreement with theoretical vertical excitation energies, particularly for the Q states. Overall, the results highlight the importance of state-specific assessment when benchmarking DH functionals for porphyrinic excitations.

## 1. Introduction

Porphyrins and their derivatives constitute an important class of chromophores renowned for their remarkable photophysical properties, which render them highly relevant across biology, chemistry, and materials science [1,2]. Consequently, these macrocycles are extensively utilized in diverse applications, including dye-sensitized solar cells [3,4,5], photocatalysis [6,7,8,9,10], nonlinear optics [11], the construction of metal–organic and covalent–organic frameworks (MOFs/COFs) [12,13,14,15], and photodynamic therapy [16,17,18,19,20]. Consequently, numerous theoretical and computational studies have been reported in the literature to elucidate their electronic excited-state properties [21,22,23,24,25,26,27]. Wavefunction-based ab initio methods have been widely deployed for porphyrinic structures [28,29,30]. Furthermore, complete active space second-order perturbation theory (CASPT2) [28,31,32], second-order multireference Moller–Pleset (MRMP2) [33,34], and second-order N-electron valence state perturbation theory (NEVPT2) [35], have been successfully implemented to yield highly accurate results. These multireference perturbation theory levels are able to accurately capture the electronic structure of quasi-degenerate states, which characterize complex configurations such as electronic excitations [36,37] and mixed-valence species [38,39,40]. Yet, while ab initio methods reliably deliver high-accuracy excitation energies for porphyrinic architectures, those robust approaches suffer from severe computational scaling, rendering them intractable for larger or structurally extended molecular systems.

Time-dependent density functional theory (TD-DFT) [41,42,43,44,45,46,47,48] has greatly impacted calculations of excitations and spectra of a wide range of molecules [49,50,51,52,53,54]. However, TD-DFT performs poorly in a number of complex situations such as double-excitations [55], long-range charge-transfer excitations [56], and ground-state/excited-state (S0/S1) conical intersections [57]. Nevertheless, due to its efficiency and relative speed, TD-DFT is still the method of choice when dealing with “real-life” medium size to large molecules. Numerous functionals spanning different levels of approximation have been assessed for porphyrins within the TD-DFT framework. For example, the hybrid functionals B3LYP, PBE0, and M06-2X, as well as the range-separated hybrids ωB97X-D, LC-BLYP, and CAM-B3LYP, have been examined for their ability to accurately predict the electronic excited states of various porphyrinic systems [27,58,59,60,61,62,63,64,65,66]. The results of these studies generally indicate that range-separated hybrid functionals, particularly CAM-B3LYP, perform reasonably well for porphyrinic molecular systems, often yielding mean absolute errors (MAEs) on the order of 0.1 eV. Nevertheless, the scope of these studies remains highly limited, with many investigations centering exclusively on one or a few porphyrin structures. Building on these limited benchmark studies, many subsequent studies of porphyrins have employed TD-DFT calculations with B3LYP [67,68,69] or CAM-B3LYP [66,70,71]. CAM-B3LYP has also been applied in TD-DFT studies involving excited-state gradients and normal-mode analysis [72]. Other functionals, such as PBE0 [70] and M05-2X [70], have likewise been used in TD-DFT calculations on porphyrins and related molecular systems.

Double-hybrid (DH) functionals include a perturbative second-order correlation contribution [73,74,75]. DHs are increasingly used in electronic excited state calculations since they provide improved accuracy, consistency, and computational efficiency for many different types of challenging chromophores [76,77,78,79,80,81,82,83,84,85,86,87,88,89,90,91,92]. In addition, DH functionals do not produce artificial ghost-states that are common in conventional hybrid functionals [76,93]. Moreover, the spin-component- and spin-opposite-scaling (SCS/SOS) schemes have been introduced in the context of DHs for excited state calculations [88,94,95,96,97]. In recent benchmark studies on challenging chromophores such as BODIPYs and cyanines, we and others found that DH functionals generally outperform lower-rung density functionals, including GGAs, hybrid-GGAs, and even range-separated hybrid-GGAs [84,85,89,91]. Indeed, the average errors obtained with some of these functionals, such as B2GP-PLYP and some SCS/SOS variants, fall within the range of those reported for the more computationally demanding ab initio methods [98,99,100], i.e., errors below 0.1 eV. In addition, Batra et al. recently assessed a broad range of functionals, including three DH functionals [71]. Their study was primarily aimed at benchmarking TD-DFT methods for large and extended biological systems, employing the Tamm–Dancoff approximation (TDA) [101] in conjunction with the simplified TDA (sTDA) [102] and simplified full TD-DFT (sTD) approaches [103]. They found that the range-separated hybrid CAM-B3LYP, combined with sTD, provides excellent accuracy, with mean absolute errors around 0.05 eV [71]. Similar results for phthalocyanines were also obtained by Martynov et al. [104]. Nevertheless, the study of Batra et al. was limited to the Q bands of the porphyrins considered, and only a small number of DH functionals were examined.

In the present work, we assess the performance of 28 DH functionals, including recent SCS/SOS variants [96], for the vertical excitation energies of 13 porphyrins and metalloporphyrins. The benchmark covers both the B and Q bands and is designed to identify DH approaches that provide consistently reliable performance across different classes of porphyrinic excitations. Particular attention is devoted to the transferability of recent SCS/SOS double hybrids between the distinct B and Q states, especially in light of their favorable performance previously reported for other chromophore classes [89,91].

## 2. Results and Discussion

### 2.1. Experimental Reference of Porphyrin Excitation Energies

**Figure 1:**
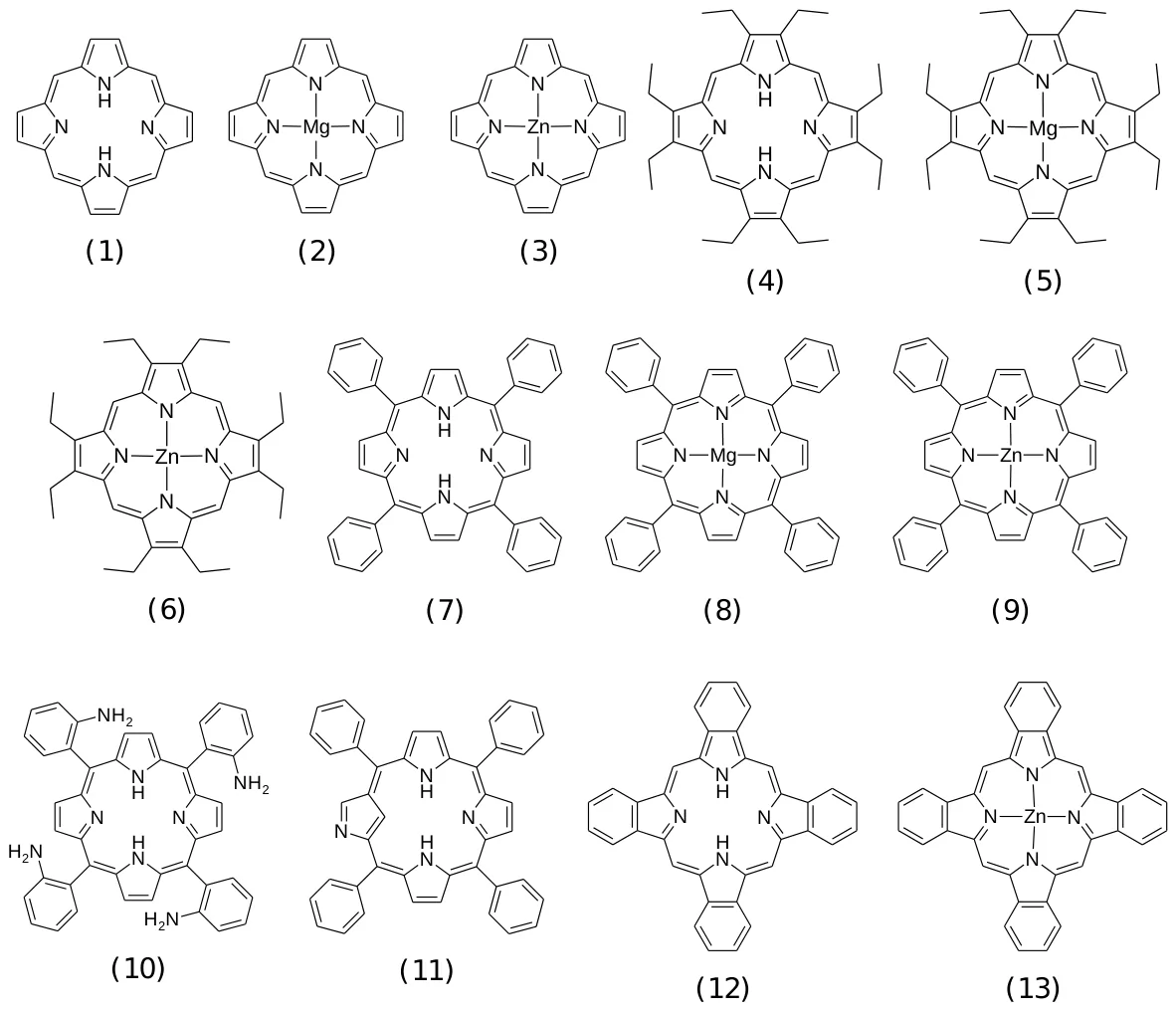
Molecular structures of porphyrins considered in the study.

A set of 13 structurally diverse porphyrin molecules was selected for the present benchmark study and is labeled **1**–**13** (Figure 1). The set comprises six free-base porphyrins and seven metalloporphyrins, including three Mg- and four Zn-containing derivatives. Several of these chromophores have been previously examined in TD-DFT benchmark investigations [66,71], which makes them particularly suitable for assessing the performance of advanced TD-DFT excited-state methods.

All compounds have published experimental UV–Vis absorption data available in the literature [105,106,107,108,109,110,111,112,113,114,115,116]. The experimental absorption maxima used throughout the present benchmark were taken exclusively from the recent PhotochemCAD database [105,106]. PhotochemCAD provides a curated compilation of absorption and fluorescence spectra together with the corresponding spectral $\lambda_{\max}$ values and experimental solvents. The experimental B-, Q1-, and Q3-band maxima used as reference values, together with the corresponding solvents, are summarized in Table 1. Although the underlying spectra compiled in PhotochemCAD originate from experimental studies performed at different times, the use of a single curated database ensures a uniform source for the reference values employed in the present benchmark.

The benchmark set considered herein was restricted to free-base, Mg, and Zn porphyrins, while porphyrins containing open-shell transition-metal centers, such as Fe(II), were not included. This choice was made in order to avoid the additional electronic-structure complications commonly associated with transition-metal systems, including stronger static correlation and more intricate spin-state energetics, which can compromise the reliability of many density-functional approximations, including double-hybrid approaches. In this respect, double-hybrid functionals are generally most reliable when static correlation remains limited. Zn-containing porphyrins were nevertheless retained in the present set because zinc is commonly excluded from the class of transition elements under the IUPAC definition [117], and Zn(II) porphyrins correspond to closed-shell $d^{10}$ systems.

### 2.2. Electronic Structure and Spectroscopy of Porphyrins

**Figure 2:**
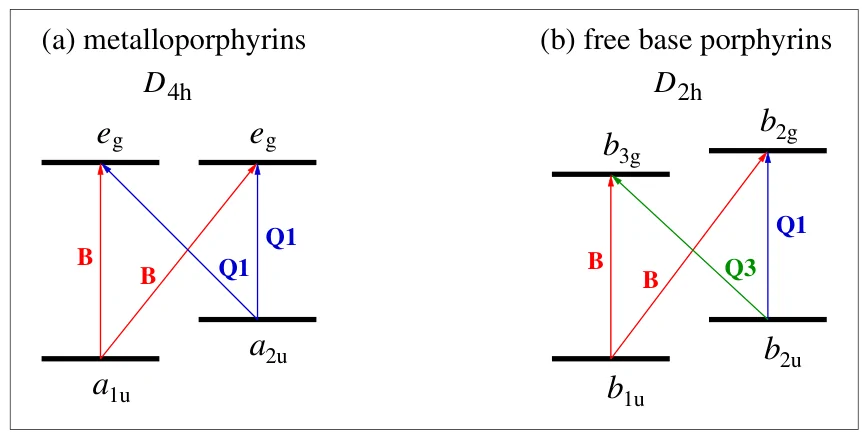
Orbital diagrams showing possible transitions for (**a**) metalloporphyrin, and (**b**) free-base porphyrins in Gouterman model.

The simplest porphyrin (free-base porphin, molecule **1** in Figure 1) is a highly conjugated macrocyclic molecule. In the Gouterman four-orbital model, both the HOMO and LUMO are degenerate pairs of orbitals [115], in which the absorption bands in porphyrin systems arise from transitions between two HOMOs and two LUMOs, and it is the identities of the metal center and the substituents on the ring that affect the relative energies of these transitions. Metalloporphyrins with $D_{4h}$ have four orbitals that are involved in the electronic transitions: two HOMOs ($a_{1u}$, $a_{2u}$) and two LUMOs ($e_{\mathrm{gx}}$, $e_{\mathrm{gy}}$). The HOMOs are not degenerate, with the $a_{1u}$ being at a lower energy. The $a_{1u}\to e_{g}$ transitions give rise to the intense B (or soret) bands (typically in the UV region) and $a_{2u}\to e_{g}$ transitions give rise to Q bands (typically in the visible region)—see Figure 2a. Replacing a metal with two hydrogen atoms (free-base porphyrins) reduces the symmetry from $D_{4h}$ to $D_{2h}$. This removes the degeneracy of the LUMO $e_{g}$ orbitals and doubles the number of observed transitions (Q1 and Q3)—see Figure 2b.

Experimentally, although reduction in symmetry from $D_{4h}$ to $D_{2h}$ lifts the degeneracy of the frontier orbitals and formally splits both Q and B excited states, the splitting is spectroscopically apparent primarily for the Q band. The B transitions remain strongly allowed and closely spaced in energy, and their splitting is typically masked by their large oscillator strength and vibronic broadening. Therefore, symmetric metalloporphyrins experimentally exhibit two Q bands (labeled Q1 and Q3), while free-base porphyrins have four bands labeled Q1, Q2, Q3, and Q4. However, quantum-chemical calculations reveal strong mixing among the configurations associated with both the B and Q bands [33,118]. As a result, the experimentally observed absorption pattern is more conveniently interpreted in terms of a reduced number of symmetry-adapted excited states: one dominant Q transition for metalloporphyrins and two main Q transitions (Q1 and Q3) for free-base porphyrins. The underlying electronic configurations mix through configuration interaction, giving rise to excited states whose transition dipole moments combine constructively for the B bands and destructively for the Q bands, which explains the much greater intensity of the former.

### 2.3. Benchmarking Protocol and Statistical Measures

In the present work, we adopt the so-called vertical excitation approximation, in which the experimental $\lambda_{\max}$ of the UV–vis absorption band is modeled as the energy of the first dipole-allowed transition between the ground and excited electronic states, evaluated at the equilibrium geometry of the ground state. One serious limitation of this vertical model is that it neglects vibronic contributions and structural relaxation effects that are inherently associated with experimental UV–vis spectra of real molecular systems. Theoretically, these effects can be accounted for by calculating $E^{0\text{-}0}$ values, which are typically compared with experimental absorption–fluorescence crossing point (AFCP) measurements [119,120,121]. However, reliable $E^{0\text{-}0}$ calculations require the evaluation of excited-state Hessians in solvent, which is computationally demanding and often impractical for medium- to large-sized molecules. Moreover, experimental AFCP values are not available for many molecular systems of practical interest. Another rigorous approach involves comparing experimental absorption spectra directly with calculated vibronic transitions rather than relying on simplified vertical excitation energies. This facilitates a much closer match in peak positions and overall band shapes [48,122]. However, evaluating vibronic profiles requires geometry optimizations of electronic excited states, which is computationally demanding and frequently hampered by non-adiabatic complexities, such as conical intersections, potential energy surface crossings, and excited-state root-flipping [48]. Consequently, in the present study we compare the experimental $\lambda_{\max}$ values with the calculated vertical excitation energies of the corresponding electronic states, as detailed below. In fact, the simplified vertical excitation approximation is widely employed in excited-state calculations and benchmark studies of electronic structure methods [71,84,85,86,87,98].

Electronic transitions were assigned to their corresponding absorption bands by selecting the lowest-energy dipole-allowed transition that matches the molecular orbital symmetry of each electronic state. In free-base porphyrins, the B bands are characterized by $b_{1u}\to b_{3g}$ transitions, while the Q1 and Q3 bands arise from $b_{2u}\to b_{2g}$ and $b_{2u}\to b_{3g}$ transitions, respectively. On the other hand, for metalloporphyrins, $a_{1u}\to e_{g}$ transitions correspond to the B bands, and $a_{2u}\to e_{g}$ transitions represent the Q1 bands. The calculated vertical excitation energies of the B, Q1, and Q3 bands for all compounds are reported in Tables S4–S35 of the Supplementary Materials, together with their electronic transitions, oscillator strengths, and molecular orbital assignments with their coefficients. The isosurface densities of the molecular orbitals involved in these transitions are shown in Figures S1–S3 of the Supplementary Materials.

**Figure 3:**
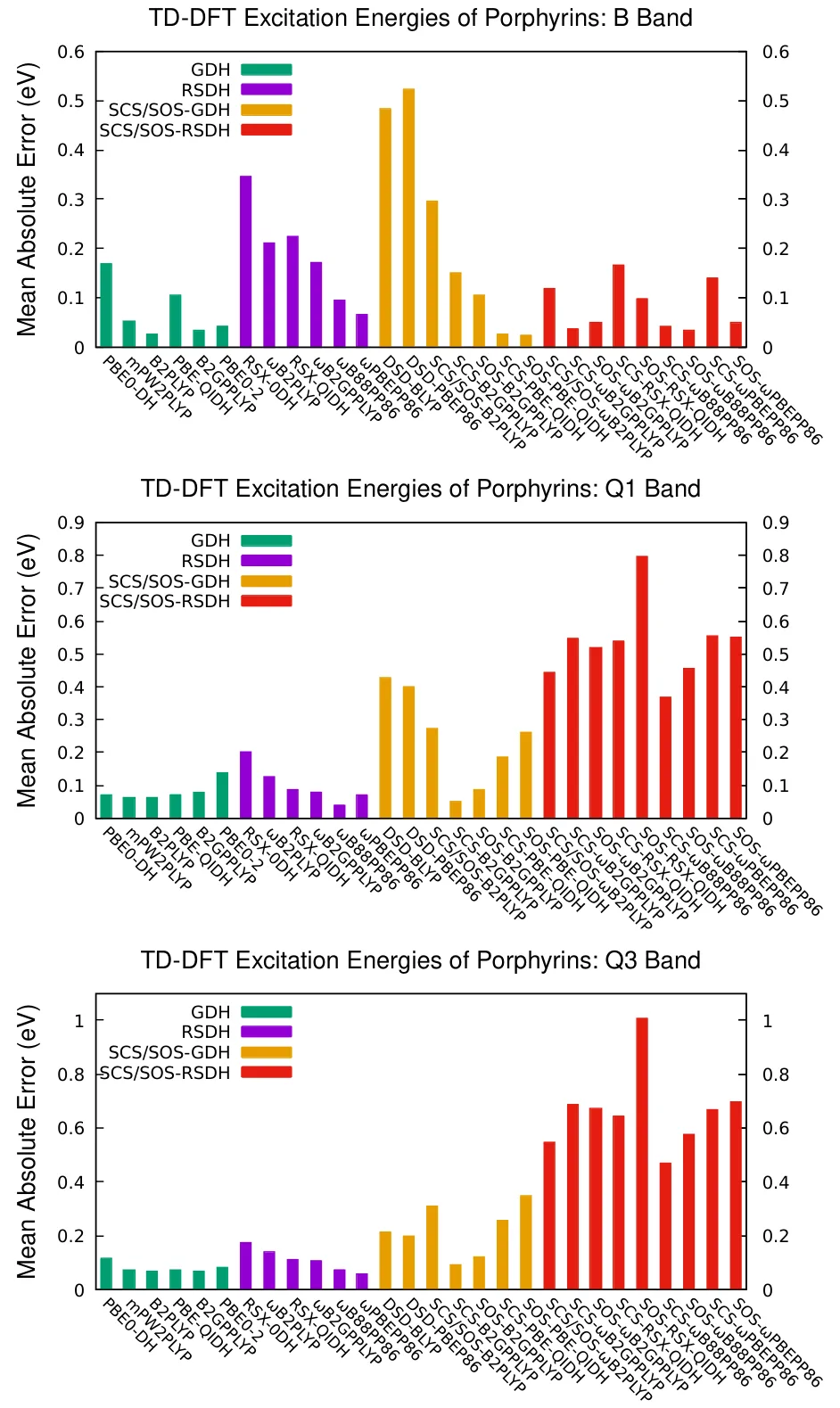
Histograms of MAE of TD-DFT excitation energies in eV of the B band (**top**), Q1 band (**center**), and Q3 (**bottom**).

Statistical error measures for all bands are provided in Tables S36–S47 of the Supplementary Materials and summarized more compactly in Tables S48–S50. These measures include the mean absolute error (MAE), mean signed error (MSE), maximum error (Max), minimum error (Min), error range ($\Delta_{\mathrm{err}}$), standard deviation of the absolute errors (SDAE), squared Pearson correlation coefficient ($R^{2}$), and the leave-one-molecule-out analysis on the MAE values (${\mathrm{MAE}}_{\mathrm{LOO}}$). Their definitions are given in Section S1 of the Supplementary Materials. In the main text below, the MAE, $\Delta_{\mathrm{err}}$, and SDAE values for the excitation energies of the three bands are summarized in Table 2, while histograms of MAE values are presented in Figure 3. For convenience, we use 0.1 eV as a practical threshold for identifying methods that reproduce the experimental absorption maxima closely within the present vertical-approximation protocol [76,123]. The performance of the different double-hybrid functionals for each electronic band is analyzed in detail in the following subsections.

### 2.4. TD-DFT Excitation Energies: The B Band

The calculated B-band excitation energies show that several of the tested DH functionals reproduce the experimental B-band maxima rather closely; see Figure 3 and Table 2. In particular, several GDH and SCS/SOS-RSDH functionals yield the comparatively low B-band MAE values, mostly below 0.2 eV, whereas a number of RSDH and SCS/SOS-GDH functionals show larger deviations. The largest deviations from the experimental B-band maxima are observed for the two empirical dispersion-corrected SCS double hybrids, DSD-BLYP and DSD-PBEP86, whose MAE values approach 0.5 eV. By contrast, the favorable B-band performance of several SCS/SOS-RSDH functionals is broadly consistent with previous benchmark results for cyanine and BODIPY chromophores, for which these approaches were also found to be particularly effective for selected classes of excitations [89,91]. In addition, some functional classes show a relationship between the B-band MAE values and the fractions of HF exchange ($a_{x}$) and perturbative correlation ($a_{c}$). This tendency is most evident within the RSDH and SCS/SOS-GDH groups, where increasing fractions of these contributions are generally accompanied by lower B-band errors, while the same behavior is not observed systematically for the GDH and SCS/SOS-RSDH functionals. To facilitate visualization of these trends, the functionals within each group in Table 2, and in Figure 3, are arranged in ascending order of their HF exchange and MP2 correlation fractions.

A considerable number of DH functionals reproduce the experimental B-band maxima with MAEs below 0.1 eV. These include mPW2PLYP, B2PLYP, B2GPPLYP, and PBE0-2 from the GDH group; ωB88PP86 and ωPBEPP86 from the RSDH group; SCS-PBE-QIDH and SOS-PBE-QIDH from the SCS/SOS-GDH group; and several members of the SCS/SOS-RSDH family. The lowest MAEs are obtained with SOS-PBE-QIDH (0.025 eV), SCS-PBE-QIDH (0.026 eV), and B2PLYP (0.026 eV). A leave-one-molecule-out analysis on the MAE values (${\mathrm{MAE}}_{\mathrm{LOO}}$) was performed to assess the robustness of this apparent ranking; Table 3 summarizes the eight functionals with the lowest full-set MAEs (${\mathrm{MAE}}_{\mathrm{Full}}$), while the complete analysis for all DH functionals is provided in Table S51 of the Supplementary Materials.

**Figure 4:**
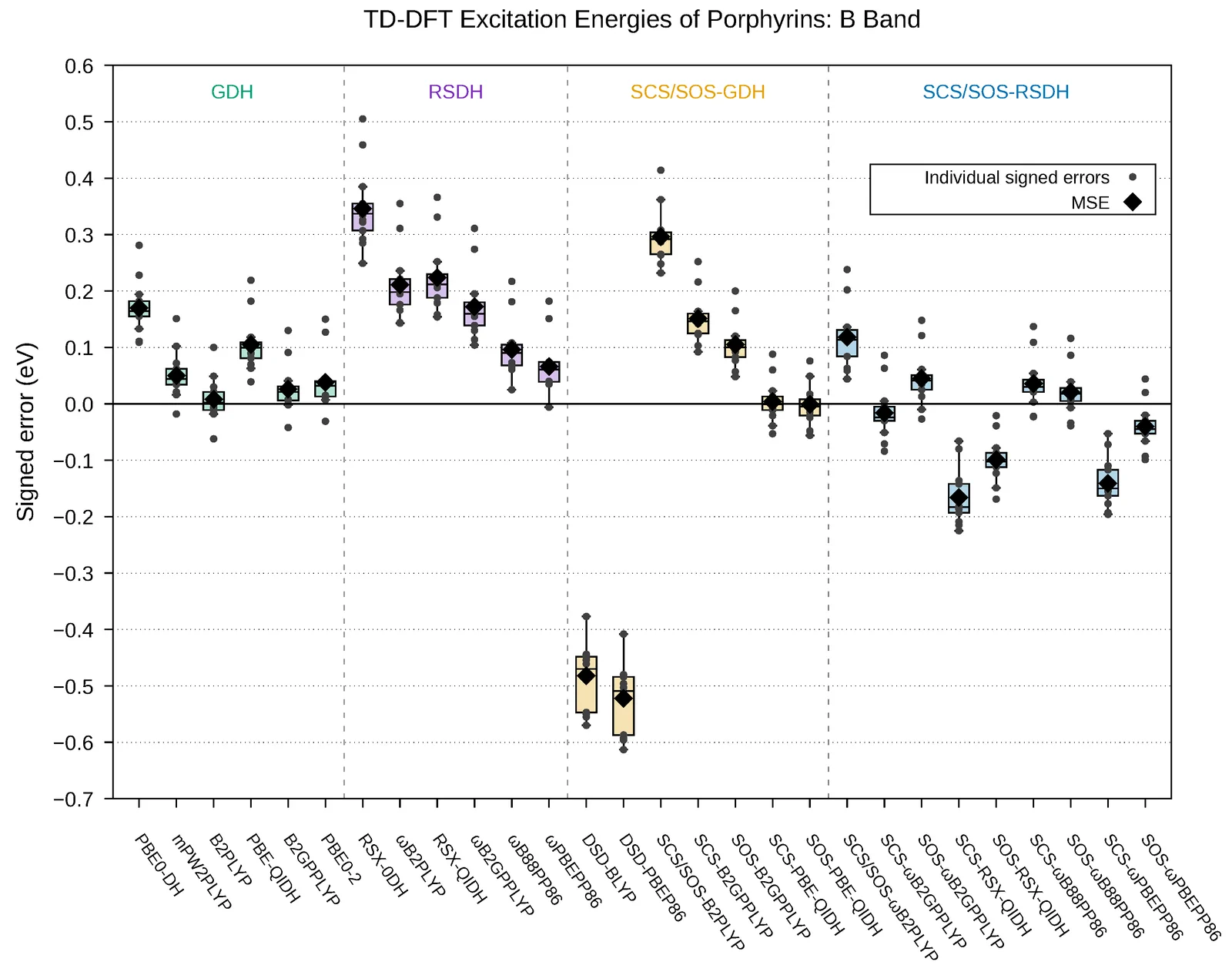
Box plots of the signed errors, $E_{i}^{\mathrm{calc}}-E_{i}^{\exp}$, for the TD-DFT B-band excitation energies. Each box represents the 14 B-band transitions included in the benchmark; the central line denotes the median, the box spans the interquartile range, and the whiskers extend to 1.5× the interquartile range. Individual signed errors are shown as points and the black diamonds denote the mean signed error (MSE).

The corresponding ${\mathrm{MAE}}_{\mathrm{LOO}}$ ranges are 0.021–0.027 eV for SOS-PBE-QIDH, 0.021–0.027 eV for SCS-PBE-QIDH, and 0.021–0.028 eV for B2PLYP, with rank ranges of 1–3, 2–3, and 1–3, respectively. Thus, although these three functionals consistently constitute the leading group, their precise ordering is sensitive to the composition of the dataset and should not be interpreted as a meaningful numerical ranking. More broadly, the favorable B-band performance is robust to removal of individual molecules, with most of the low-MAE functionals retaining similarly small deviations throughout the LOO analysis. These results support treating the leading methods as a top-performing group rather than assigning significance to differences of only a few thousandths of an eV. More importantly, the signed-error distributions shown in Figure 4 provide a more detailed picture of the B-band performance. The errors obtained with B2PLYP, SCS-PBE-QIDH, and SOS-PBE-QIDH are centered close to zero, consistent with their very small MAEs, with no pronounced systematic over- or underestimation across the benchmark set. In general, however, clear directional biases are apparent for several functional families. For instance, most RSDH functionals show predominantly positive errors, corresponding to an overestimation of the B-band excitation energies, whereas DSD-BLYP and DSD-PBEP86 show a pronounced systematic underestimation. Several SCS/SOS-RSDH functionals, by contrast, exhibit error distributions centered much closer to zero, consistent with their comparatively favorable B-band MAEs. Thus, the low MAEs of the best-performing methods do not arise simply from cancellation between large positive and negative deviations, although appreciable molecule-to-molecule variation remains.

**Figure 5:**
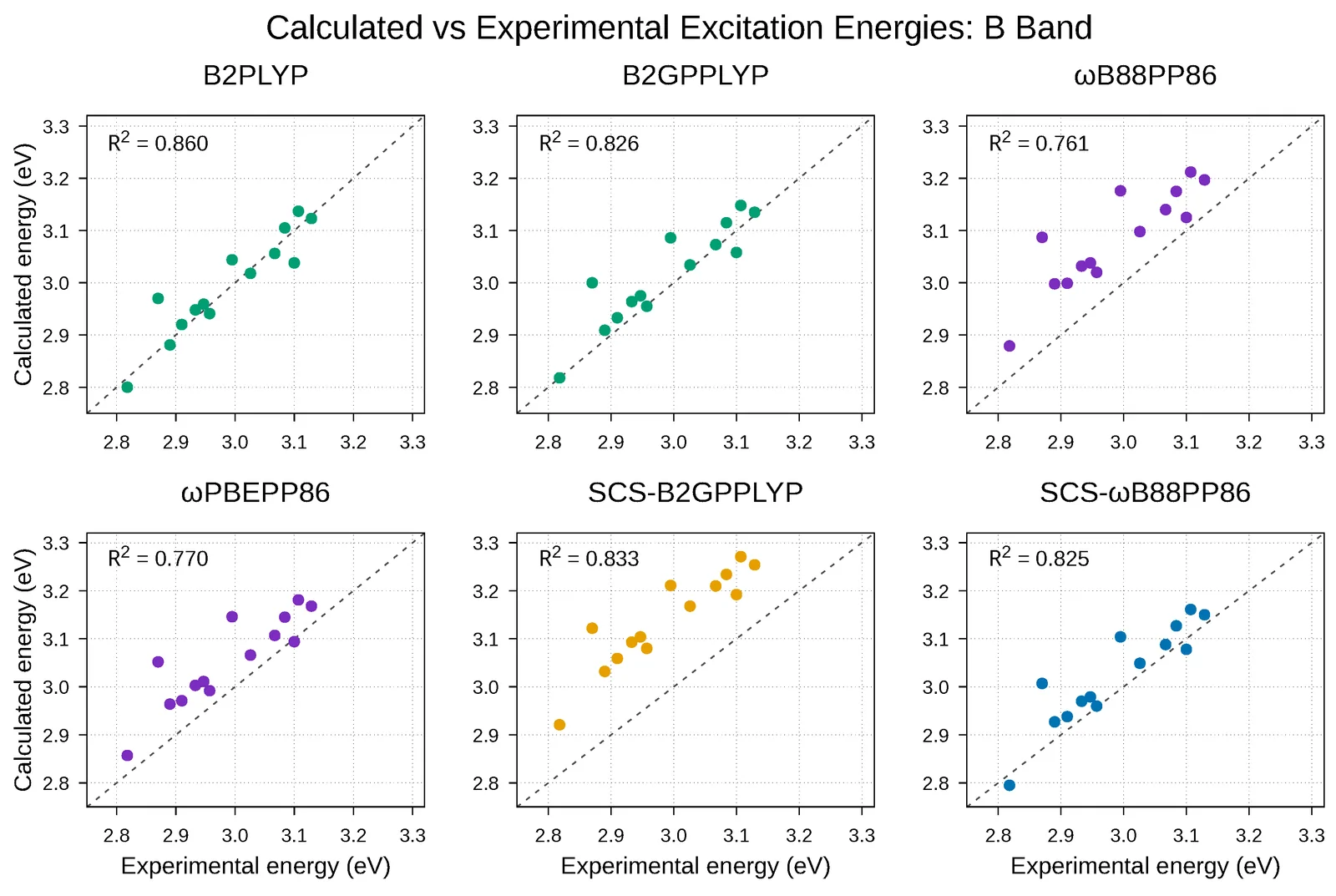
Calculated versus experimental B-band excitation energies for selected representative DH functionals. The dashed diagonal line corresponds to perfect agreement ($E_{i}^{\mathrm{calc}}=E_{i}^{\exp}$); the corresponding coefficients of determination ($R^{2}$) are shown in each panel.

The calculated-versus-experimental parity plots for selected representative functionals (Figure 5) further illustrate these trends. B2PLYP and B2GPPLYP show points lying relatively close to the ideal $E_{i}^{\mathrm{calc}}=E_{i}^{\exp}$ line, whereas ωB88PP86 and ωPBEPP86 exhibit a more evident positive displacement, consistent with their predominantly positive signed errors. Particularly instructive is SCS-B2GPPLYP, which retains a relatively high $R^{2}$ value of 0.833 despite a systematic upward displacement from the parity line and a substantially larger MAE of 0.151 eV. This illustrates that a high $R^{2}$ reflects the ability to reproduce relative variations across the molecular series, but does not by itself imply close absolute agreement with experiment. By comparison, SCS-ωB88PP86 shows both a smaller systematic displacement and a low MAE of 0.042 eV. Overall, the parity analysis corroborates the favorable B-band performance of several DH approaches within the vertical approximation, while emphasizing the complementary information provided by measures of absolute error, signed bias, and correlation.

The free-base tetrabenzoporphine (molecule **12**) is the only member of the present set that exhibits two experimentally resolved bands in the B-transition region. This behavior likely reflects the stronger perturbation of the porphyrinic π system induced by benzannulation, which increases the energetic separation between the two closely lying B-type excited states. Indeed, some studies on fused porphyrins have explicitly reported split Soret bands in similarly perturbed π-extended systems [124,125,126]. For most of the other porphyrins, these states remain unresolved experimentally and appear as a single intense Soret band because of their small energy separation and substantial spectral broadening. The fact that two B-region transitions are also obtained in the calculated spectrum of compound **12** further supports this interpretation. Accordingly, both experimentally resolved B-band transitions of molecule **12**, together with their corresponding calculated states, were included as separate data points in the B-band statistical analysis, giving a total of 14 B-band transitions for the 13 molecules.

It is also instructive to distinguish between the two subclasses represented in the benchmark set, namely free-base porphyrins (**1**, **4**, **7**, **10**, **11**, and **12**) and metalloporphyrins (**2**, **3**, **5**, **6**, **8**, **9**, and **13**). For the free-base porphyrins, the MAE values obtained for the three functionals yielding the lowest MAEs relative to the experimental maxima are 0.025 eV for B2PLYP, 0.029 eV for SCS-PBE-QIDH, and 0.031 eV for SOS-PBE-QIDH. For the metalloporphyrins, the corresponding MAE values are 0.028, 0.022, and 0.019 eV, respectively. These values are relatively close to the overall MAEs obtained for the full benchmark set, indicating similar levels of agreement with the experimental B-band maxima for the free-base and Mg/Zn-containing subsets. This suggests that their agreement with the experimental B-band maxima is not strongly dependent on whether the porphyrin is free-base or Mg/Zn-containing within the present dataset.

### 2.5. TD-DFT Excitation Energies: The Q1 and Q3 Bands

The Q1 and Q3 bands show considerably larger deviations from the experimental absorption maxima than the B band for many of the tested DH functionals. Most notably, the SCS/SOS-RSDH group, which performs rather well for the B band, shows the largest deviations from the experimental Q-band maxima, with several members exhibiting MAE values above 0.5 eV (Figure 3 and Table 2). This behavior is particularly noteworthy because it contrasts sharply with previous benchmark results for closely related cyanine and BODIPY chromophores, as well as with the generally good performance of these functionals for other classes of excited states [88,89,91,96]. By contrast, the spin-unscaled GDH and RSDH functionals provide, on average, substantially closer agreement with the experimental Q-band maxima. However, unlike the B band calculated using GDH and RSDH functionals, there is no clear correlation between the MAE values of these functionals and the fractions of the HF exchange and MP2 correlation components; see Figure 3.

For the Q1 band, most GDH functionals, together with ωB88PP86 and ωPBEPP86 from the RSDH group and SCS-B2GPPLYP from the SCS/SOS-GDH group, yield MAEs below 0.1 eV relative to the experimental absorption maxima. The lowest MAEs are obtained with ωB88PP86 (∼0.04 eV) and SCS-B2GPPLYP (∼0.05 eV), followed by B2PLYP and mPW2PLYP (both ∼0.06 eV). A leave-one-molecule-out analysis of the MAE values was also performed to assess the robustness of these results; the leading results are summarized in Table 3, while the complete analysis is reported in Table S52 of the Supplementary Materials. Notably, ωB88PP86 remains ranked first in all 13 leave-one-out subsets, with ${\mathrm{MAE}}_{\mathrm{LOO}}$ values ranging from 0.031 to 0.045 eV, while SCS-B2GPPLYP remains consistently ranked second, with a corresponding range of 0.040–0.053 eV. B2PLYP and mPW2PLYP show similarly stable performance, with ${\mathrm{MAE}}_{\mathrm{LOO}}$ ranges of 0.051–0.066 and 0.051–0.067 eV, respectively, and interchange only between ranks 3 and 4. Thus, unlike the Q3 subset, for which the ordering of the leading functionals is more sensitive to dataset composition (see below), the Q1 results show a comparatively robust leading hierarchy.

**Figure 6:**
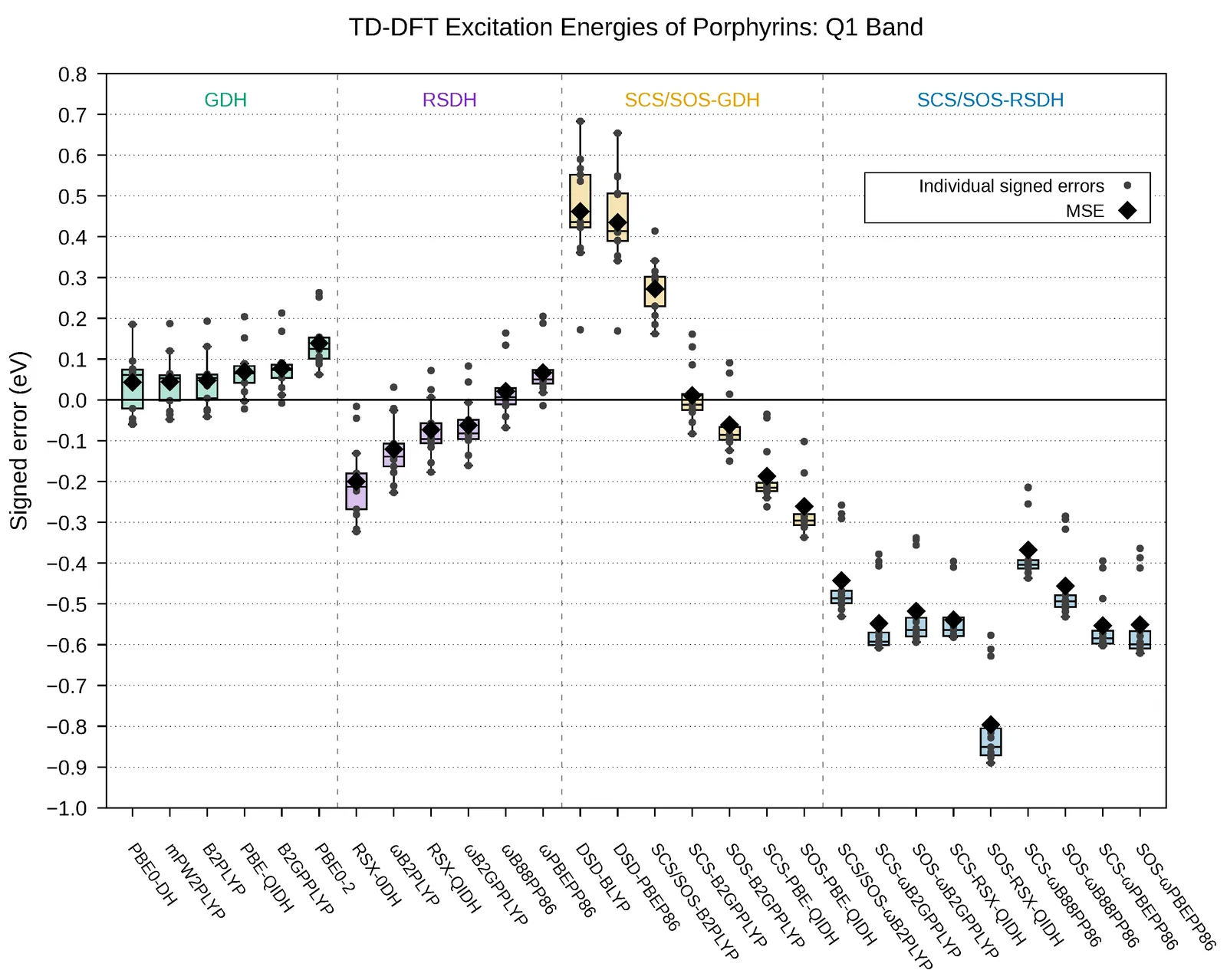
Box plots of the signed errors, $E_{i}^{\mathrm{calc}}-E_{i}^{\exp}$, for the TD-DFT Q1-band excitation energies. Each box represents the 13 Q1-band transitions included in the benchmark; the central line denotes the median, the box spans the interquartile range, and the whiskers extend to 1.5× the interquartile range. Individual signed errors are shown as points and the black diamonds denote the mean signed error (MSE).

The signed-error distributions in Figure 6 provide a more detailed view of the Q1-band performance. Although the leading functionals exhibit relatively small MAEs, their individual errors show appreciable molecule-to-molecule variation, and therefore the low average deviations should not be interpreted as indicating uniformly small errors across the entire benchmark set. Among the best-performing methods, ωB88PP86 and SCS-B2GPPLYP show distributions centered close to zero, with only modest overall signed biases. B2PLYP, B2GPPLYP, and ωPBEPP86 display somewhat more pronounced positive biases, whereas several RSDH functionals predominantly underestimate the Q1 excitation energies. Particularly striking is the behavior of the SCS/SOS-RSDH family, for which the signed errors are almost exclusively negative and substantially displaced from zero. Thus, the relatively large Q1 MAEs of these functionals reflect a systematic underestimation of the experimental absorption maxima rather than merely a broad or irregular distribution of positive and negative errors.

**Figure 7:**
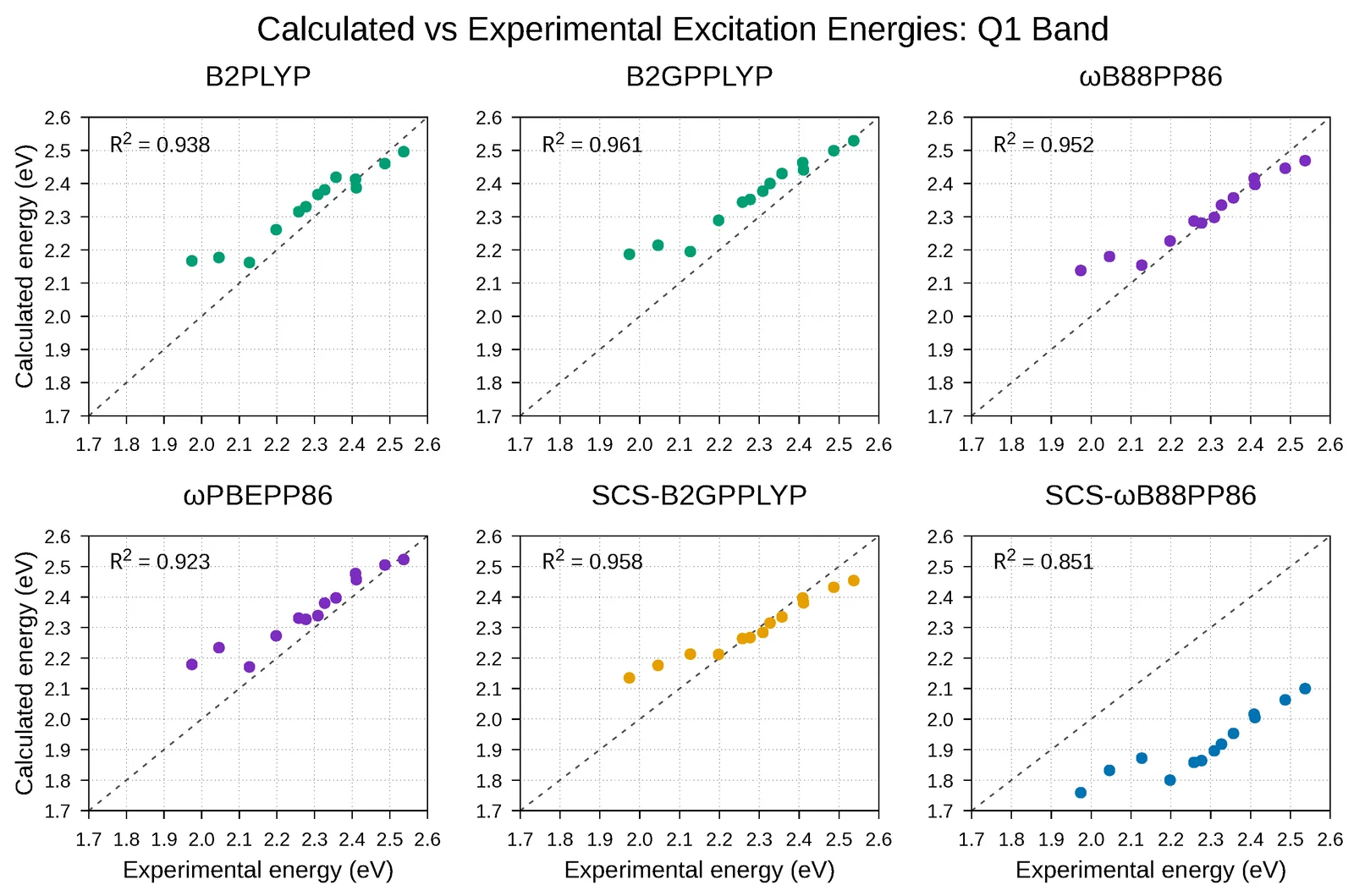
Calculated versus experimental Q1-band excitation energies for selected representative DH functionals. The dashed diagonal line corresponds to perfect agreement ($E_{i}^{\mathrm{calc}}=E_{i}^{\exp}$); the corresponding coefficients of determination ($R^{2}$) are shown in each panel.

The parity plots in Figure 7 further emphasize these trends. ωB88PP86 and SCS-B2GPPLYP show particularly favorable agreement, combining low MAEs with high $R^{2}$ values of 0.952 and 0.958, respectively, and data points distributed relatively close to the ideal $E_{i}^{\mathrm{calc}}=E_{i}^{\exp}$ line. B2PLYP, B2GPPLYP, and ωPBEPP86 also reproduce the relative variation across the molecular series well, with $R^{2}$ values of 0.938, 0.961, and 0.923, respectively, although their points show a modest systematic displacement toward higher calculated energies. In marked contrast, SCS-ωB88PP86 exhibits a pronounced downward displacement from the parity line, consistent with its systematic negative signed errors and substantially larger MAE. Nevertheless, its $R^{2}$ value remains relatively high (0.851), again illustrating that correlation with experimental trends does not necessarily imply close absolute agreement with the experimental absorption maxima. Taken together, the signed-error and parity analyses reinforce the favorable Q1 performance of selected GDH, RSDH, and SCS/SOS-GDH functionals while revealing a clear systematic underestimation by the SCS/SOS-RSDH family.

A similar picture emerges for the Q3 band, which is specific to the lower-symmetry free-base porphyrins. Here again, most GDH functionals, along with ωB88PP86 and ωPBEPP86, reproduce the experimental Q3-band maxima with MAEs below 0.1 eV. The lowest MAE values are obtained with ωPBEPP86 (∼0.06 eV), B2GPPLYP (∼0.07 eV), and B2PLYP (∼0.07 eV). Given the small size of the Q3 subset (N=6), a leave-one-molecule-out analysis on the MAE values was performed to assess the robustness of these results; the leading results are summarized in Table 3 (see also Table S53 of the Supplementary Materials for all DH functionals). The corresponding ${\mathrm{MAE}}_{\mathrm{LOO}}$ ranges are 0.043–0.071 eV for ωPBEPP86, 0.042–0.080 eV for B2GPPLYP, and 0.043–0.079 eV for B2PLYP, with rank ranges of 1–3, 1–4, and 2–4, respectively. Thus, the precise ordering of these leading functionals is sensitive to the composition of the small dataset and should not be interpreted as a rigorous ranking. Nevertheless, the broader performance trends remain stable: the functionals yielding the lowest overall MAEs remain close to the experimental Q3-band maxima upon removal of any single molecule, whereas the SCS/SOS-RSDH variants continue to exhibit substantially larger deviations (see Table S53 in the Supplementary Materials). Taken together, the results for the Q1 and Q3 bands indicate that unscaled GDH functionals and selected RSDHs generally provide closer agreement with the experimental Q-band maxima than the more recent SCS/SOS-RSDH variants, despite the latter showing excellent performance for the B band.

**Figure 8:**
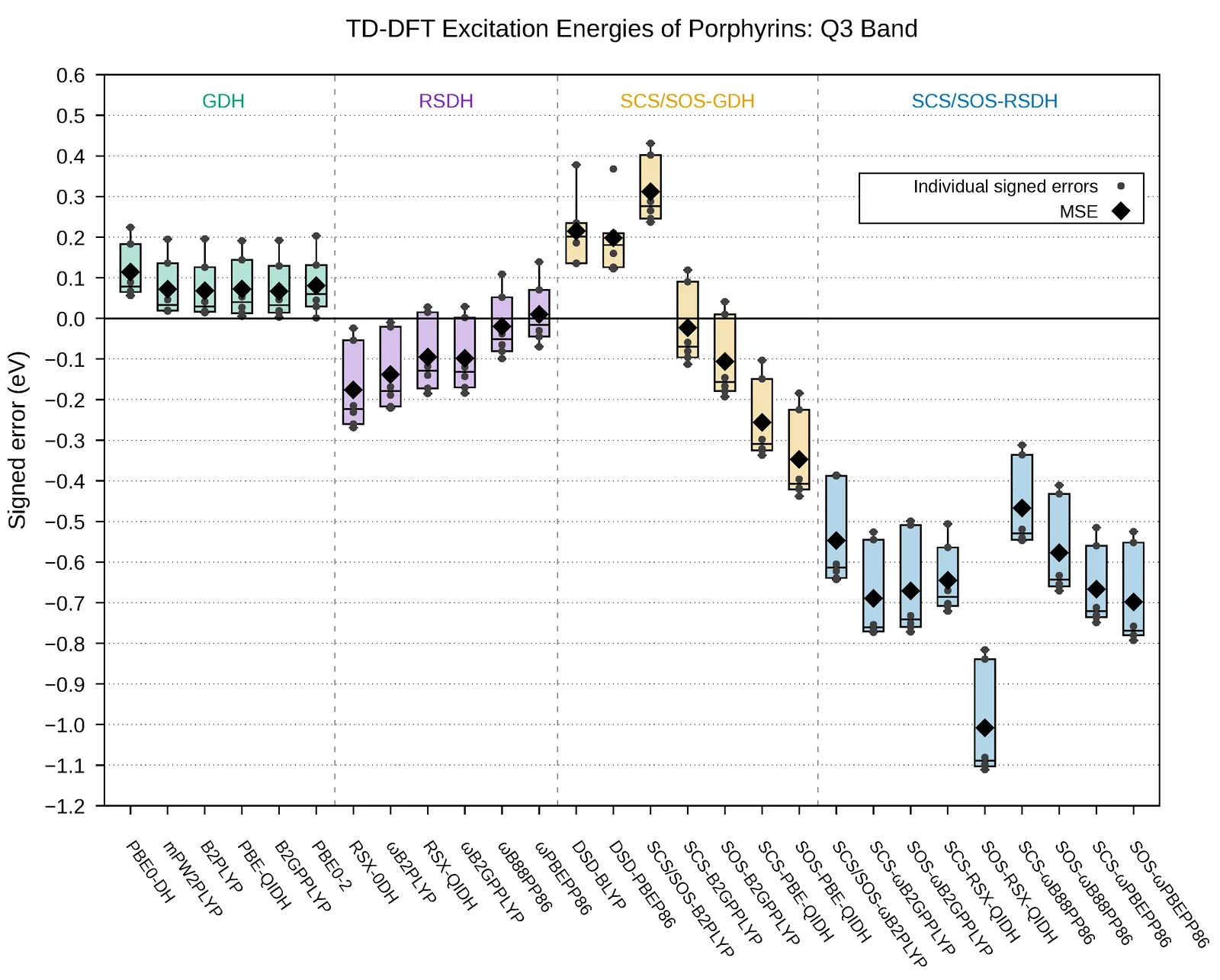
Box plots of the signed errors, $E_{i}^{\mathrm{calc}}-E_{i}^{\exp}$, for the TD-DFT Q3-band excitation energies. Each box represents the six Q3-band transitions included in the benchmark; the central line denotes the median, the box spans the interquartile range, and the whiskers extend to 1.5× the interquartile range. Individual signed errors are shown as points and the black diamonds denote the mean signed error (MSE).

The signed-error distributions shown in Figure 8 provide a more direct view of the Q3-band behavior. Given the small size of this subset (N=6), these distributions should be interpreted cautiously, with particular attention to the individual data points rather than to the box statistics alone. The better-performing GDH and selected RSDH functionals generally exhibit errors relatively close to zero, although modest systematic positive or negative biases remain for several methods. SCS-B2GPPLYP likewise maintains comparatively small deviations, consistent with its Q3-band MAE of 0.093 eV. In marked contrast, the SCS/SOS-RSDH functionals display predominantly negative signed errors that are substantially displaced from zero. Their large Q3-band MAEs therefore reflect a pronounced systematic underestimation of the experimental absorption maxima rather than being driven by only one or two anomalous molecules. This family-level behavior remains evident despite the limited size of the Q3 dataset and is consistent with the robustness trends obtained from the LOO analysis.

**Figure 9:**
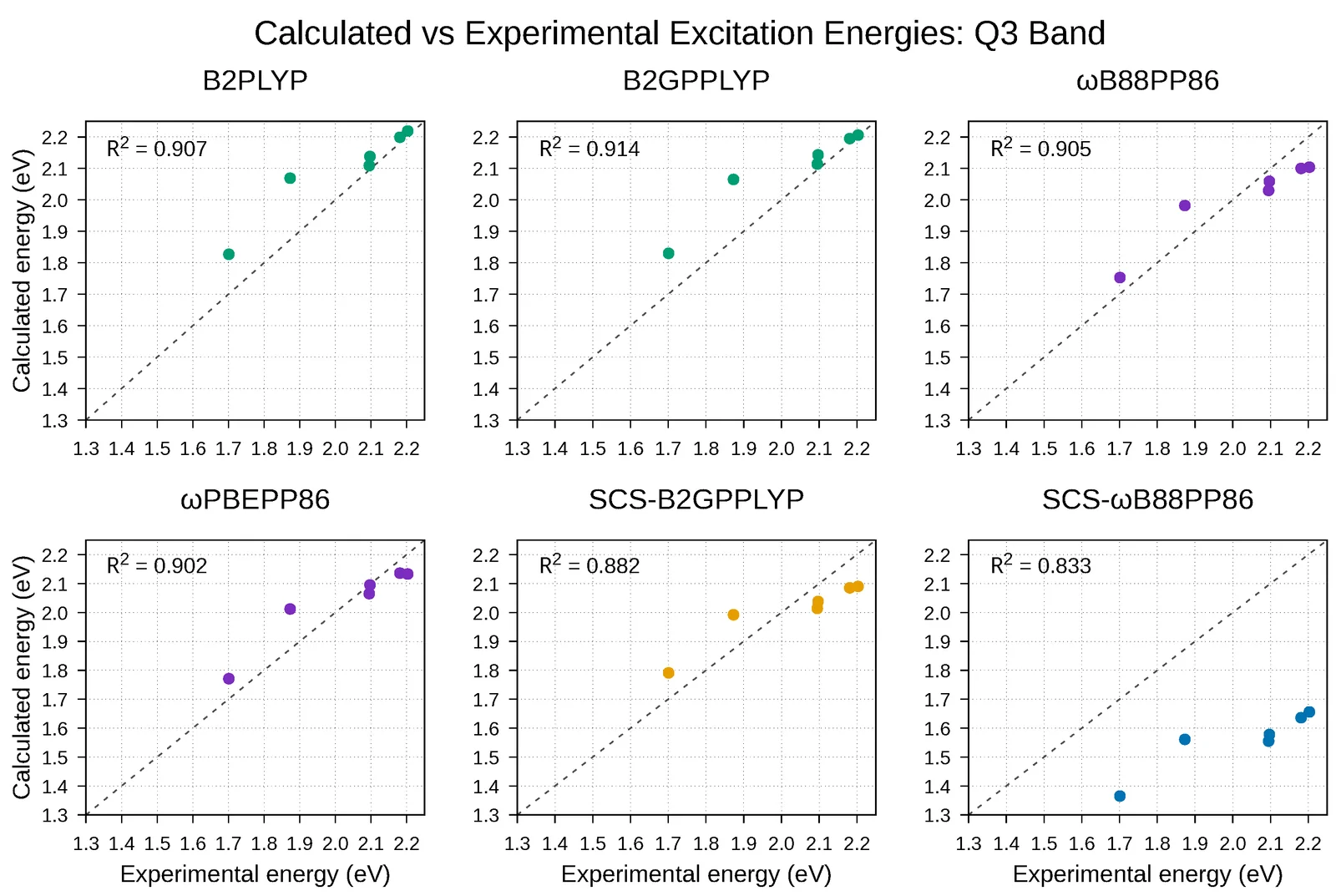
Calculated versus experimental Q3-band excitation energies for selected representative DH functionals. The dashed diagonal line corresponds to perfect agreement ($E_{i}^{\mathrm{calc}}=E_{i}^{\exp}$); the corresponding coefficients of determination ($R^{2}$) are shown in each panel.

The parity plots in Figure 9 further illustrate these differences. B2PLYP, B2GPPLYP, ωB88PP86, and ωPBEPP86 all reproduce the relative variation across the six Q3 transitions reasonably well, with $R^{2}$ values between 0.902 and 0.914, while their calculated energies remain comparatively close to the ideal parity line. Among these methods, ωPBEPP86 provides the lowest Q3-band MAE (0.059 eV), whereas B2GPPLYP gives the highest $R^{2}$ value (0.914). SCS-B2GPPLYP also retains a relatively high correlation ($R^{2}=0.882$) together with a moderate MAE of 0.093 eV. By contrast, SCS-ωB88PP86 shows a pronounced downward displacement from the parity line despite retaining an $R^{2}$ value of 0.833, demonstrating once more that reproduction of relative trends does not necessarily imply close absolute agreement with the experimental absorption maxima. Because only six Q3 transitions are available, however, small differences among the individual $R^{2}$ values should not be overinterpreted. Taken together, the Q3 signed-error and parity analyses reinforce the broader conclusion that unscaled GDH and selected RSDH functionals generally reproduce the experimental Q-band maxima more closely than the SCS/SOS-RSDH variants, despite the excellent B-band performance of several members of the latter family.

An overall assessment of the three bands shows that B2PLYP provides one of the most balanced agreements with the experimental absorption maxima within the present vertical-approximation protocol, yielding MAEs of ∼0.03, ∼0.06, and ∼0.07 eV for the B, Q1, and Q3 bands, respectively, while remaining among the leading methods in the LOO analyses. B2GPPLYP, ωB88PP86, and ωPBEPP86 likewise provide consistently close overall agreement, with MAEs below 0.1 eV for all three bands. Particularly noteworthy is the pronounced band dependence of several recent SCS/SOS-RSDH functionals: although some of these methods yield very small deviations for the B band, their Q1- and Q3-band errors show strong systematic negative biases and substantially larger MAEs. This behavior highlights the importance of assessing different classes of porphyrinic excitations separately and demonstrates that favorable performance for the intense B band does not necessarily transfer to the lower-energy Q bands. Representative computational timings obtained for molecule 1 under identical hardware and computational settings show that the different DH functionals have broadly comparable computational costs, with wall times ranging from approximately 22 to 27 min; thus, no substantial computational-cost penalty is associated with the better-performing functionals considered here (Table S54 of the Supplementary Materials).

In contrast to Batra et al. [71], who reported comparatively large errors for the three DH functionals considered in their porphyrinoid benchmark (B2PLYP, B2GPPLYP, and mPW2PLYP), the present calculations yield substantially smaller deviations from the experimental Q-band maxima for these methods. In particular, all three functionals provide Q1- and Q3-band MAEs below 0.1 eV in the present dataset. The origin of this difference cannot be assigned unambiguously to a single computational factor, since the two studies differ in several aspects of their computational protocols, including the basis set and the treatment of the excited states: Batra et al. employed the smaller double-zeta def2-SVP basis set in conjunction with simplified TD-DFT/TDA-based approaches, whereas the present calculations use triple-zeta def2-TZVP within the full TD-DFT framework. The consistently small deviations obtained with B2PLYP across the three experimental band sets are particularly noteworthy, given that it is one of the earliest DH functionals, originally proposed by Grimme in 2006 [73].

### 2.6. Comparison with High-Level Theoretical Vertical Excitation Energies

As discussed in Section 2.3, direct comparison of calculated vertical excitation energies with experimental absorption maxima is subject to a significant limitation, because the latter include vibronic and structural-relaxation effects, as well as environmental contributions that are not fully represented within the vertical approximation. Therefore, to provide a more direct assessment of the calculated vertical excitation energies, we additionally compare selected DH results with published high-level theoretical vertical excitation energies for the parent free-base porphin (molecule **1**), Mg-porphin (molecule **2**), and Zn-porphin (molecule **3**). These systems were selected because multireference perturbation-theory results are available for the corresponding electronic states. Table 4 summarizes the published MRMP [33], improved-virtual-orbital MRMP (IVO-MRMP) [34], and CASPT2 [31,32] vertical excitation energies, together with representative TD-DHDFT values obtained in the present work. The DH functionals were selected to include the “best-performing” representatives from each functional family. For completeness, the corresponding experimental absorption maxima are also included for reference, but are not treated here as equivalent to theoretical vertical excitation energies.

Before comparing the DH results with these high-level data, it is important to note that the degree of agreement among the published multireference vertical excitation energies is strongly state-dependent. It should also be emphasized that the three multireference approaches were based on different active spaces, namely CAS(8e,8o) for MRMP [33], CAS(16e,12o) for IVO-MRMP [34], and CAS(14e,16o) for CASPT2 [31,32], which may contribute to the differences among the reported excitation energies. For free-base porphin (**1**), the three methods agree remarkably well for the B transition, yielding values between 3.06 and 3.12 eV. In contrast, the corresponding Q1 energies span a much wider interval, from 2.11 eV with CASPT2 to 2.55 and 2.64 eV with MRMP and IVO-MRMP, respectively. A smaller but still appreciable spread is also found for Q3, for which MRMP and CASPT2 both give 1.63 eV, whereas IVO-MRMP yields 1.86 eV. An analogous situation is observed for Mg-porphin (**2**): the predicted B-band energies range from 2.66 eV with CASPT2 to 3.07 and 3.15 eV with MRMP and IVO-MRMP, while the corresponding Q1 values range from 1.66 to 2.14 eV. For Zn-porphin (**3**), for which corresponding CASPT2 values are not available, MRMP and IVO-MRMP differ by 0.16 eV for the B state and 0.17 eV for Q1. These differences indicate that even high-level theoretical vertical excitation energies cannot always be regarded as unambiguous reference values for porphyrinic excited states. In addition to the different active spaces noted above, part of the observed spread may arise from differences in the underlying perturbative treatments, basis sets, and ground-state geometries employed in the original studies. In this context, Chaudhuri et al. explicitly noted that the available literature calculations employed different geometries and theoretical protocols, and their comparison likewise revealed non-negligible differences among multireference treatments [34].

For free-base porphin (**1**), the B-state results show a particularly consistent picture. B2PLYP and B2GPPLYP predict excitation energies of 3.12 and 3.14 eV, respectively, which lie very close to the 3.06–3.12 eV range spanned by the high-level MRMP, IVO-MRMP, and CASPT2 calculations. The range-separated ωB88PP86 and ωPBEPP86 functionals also remain reasonably close, although they yield slightly higher energies. Thus, for the B state, the favorable performance inferred from comparison with the experimental absorption maximum is largely corroborated by the high-level vertical-excitation calculations. The situation is considerably more complex for the Q states. For Q1, B2PLYP, B2GPPLYP, ωB88PP86, and ωPBEPP86 yield values of 2.47–2.53 eV, in excellent agreement with the MRMP value of 2.55 eV and reasonably close to the IVO-MRMP result of 2.64 eV, but substantially above the CASPT2 value of 2.11 eV. Conversely, SCS-ωB88PP86 and SOS-ωB88PP86 yield 2.10 and 2.01 eV, respectively, and therefore agree much more closely with CASPT2. This dependence on the choice of high-level reference becomes even more pronounced for Q3. Whereas the spin-unscaled DH values lie between 2.10 and 2.22 eV and closely reproduce the experimental absorption maximum of 2.20 eV, the MRMP and CASPT2 vertical excitation energies are both substantially lower, at 1.63 eV, while IVO-MRMP gives 1.86 eV. Interestingly, SCS-ωB88PP86 yields 1.66 eV, in very close agreement with the MRMP and CASPT2 values. Thus, for the Q states of compound **1**, close agreement with the experimental absorption maximum does not necessarily translate into equally close agreement with high-level theoretical vertical excitation energies.

The Mg- and Zn-containing porphyrins (**2** and **3**) show a broadly similar pattern. For Mg-porphin (**2**), most of the selected DH functionals predict B-band excitation energies in the range 3.11–3.23 eV, in close agreement with the MRMP (3.07 eV) and IVO-MRMP (3.15 eV) results, whereas the CASPT2 value is substantially lower, at 2.66 eV. The experimental absorption maximum of 3.08 eV is also very close to the MRMP value, indicating that the favorable DH performance for this particular B transition is independently supported by at least two of the available high-level vertical-excitation estimates and is therefore not solely a consequence of comparison with $\lambda_{\max}$. For Q1, however, the spin-unscaled DH values of approximately 2.30–2.38 eV lie close to the experimental maximum of 2.31 eV but above the published multireference vertical excitation energies, which span 1.66–2.14 eV. In this case, the lower excitation energies obtained with the spin-scaled variants lie considerably closer to the multireference results; for example, SCS-ωB88PP86 privides 1.90 eV. For Zn-porphin (**3**), the DH B-state energies generally fall within or close to the MRMP/IVO-MRMP range of 3.21–3.37 eV, whereas the experimental absorption maximum is somewhat lower, at 3.11 eV. For Q1, the available MRMP and IVO-MRMP values are 2.11 and 2.28 eV, respectively, while the selected DH functionals span values both above and below this range. For example, SOS-B2GPPLYP predicts 2.26 eV, in very close agreement with IVO-MRMP, whereas the spin-unscaled functionals generally yield somewhat higher excitation energies.

Taken together, this comparison provides a more nuanced interpretation of the benchmark based on experimental absorption maxima. For the B transitions, particularly for molecules **1** and **2**, the favorable performance of several DH functionals is largely corroborated by the available high-level multireference vertical excitation energies. For the Q states, however, the situation is less straightforward: the high-level theoretical values themselves exhibit substantial method dependence, and in several cases the DH functionals that most closely reproduce the experimental $\lambda_{\max}$ values are not those that lie closest to the published multireference vertical excitation energies. This observation underscores that the statistical analysis based on experimental absorption maxima should primarily be interpreted as an assessment of how well the different DH functionals reproduce the observed spectral positions within the vertical approximation, rather than as an unambiguous ranking of their intrinsic accuracy for vertical excitation energies. It also indicates that the comparatively large deviations of some SCS/SOS functionals from the experimental Q-band maxima should not automatically be interpreted as correspondingly large errors in the underlying vertical excitation energies. Nevertheless, the present comparison demonstrates that several DH approaches yield vertical excitation energies of the same general quality as much more computationally demanding multireference methods, while also emphasizing the strong state dependence of their relative performance.

### 2.7. Effect of Geometry on Excitation Energies

The ground-state geometries of all molecules were optimized using the hybrid PBE0 functional. PBE0 has previously been shown to provide accurate ground-state geometries for closely related BODIPY and cyanine dyes [127]. To assess the sensitivity of the calculated vertical excitation energies to the choice of geometry-optimization functional, additional geometry optimizations were performed using B3LYP and M06-2X for a representative subset comprising molecules **1**, **2**, and **7**. The optimized ground-state Cartesian coordinates of all molecules (**1**–**13**) obtained with PBE0 are reported in Section S2, Table S1 of the Supplementary Materials, whereas the corresponding geometries of molecules **1**, **2**, and **7** obtained with B3LYP and M06-2X are provided in Tables S2 and S3 of Section S2 of the Supplementary Materials, respectively.

Table 5 summarizes selected structural parameters, including key bond lengths and dihedral angles of molecules **1**, **2**, and **7** optimized with PBE0, B3LYP and M06-2X. In this Table, N⋯N denotes the distance between the two opposing inner nitrogen atoms of the porphyrin macrocycle, whereas H⋯H denotes the distance between the two inner hydrogen atoms bonded to the pyrrolic nitrogens in the free-base porphyrins. The phenyl dihedral angle denotes the angle between the mean plane of the porphyrin macrocycle and the plane of the rotatable phenyl ring in molecule **7**.

As shown in Table 5, the three functionals provide broadly similar geometries for the porphyrin core, although PBE0 and M06-2X generally show the closest agreement. For molecule **1**, the N⋯N distance obtained with PBE0 (4.032 Å) differs by only 0.011 Å from the M06-2X value (4.021 Å), whereas B3LYP predicts a slightly larger value of 4.063 Å. Similar trends are observed for molecule **7**, for which the corresponding N⋯N distances are 4.049, 4.076, and 4.044 Å with PBE0, B3LYP, and M06-2X, respectively. The H⋯H distances also show only modest variations among the three functionals. For the Mg-containing molecule **2**, the Mg–N bond length is particularly insensitive to the optimization functional, varying by only 0.013 Å across the three methods. A substantially larger functional dependence is observed, however, for the orientation of the peripheral phenyl groups in molecule **7**. PBE0 and M06-2X predict very similar phenyl dihedral angles of 73.0° and 71.6°, respectively, whereas B3LYP yields a considerably larger value of 89.7°, corresponding to an almost perpendicular orientation of the phenyl ring relative to the porphyrin macrocycle. Overall, these results indicate that the optimized porphyrin-core geometries are relatively insensitive to the choice among the three functionals, whereas more flexible torsional degrees of freedom may exhibit a substantially stronger functional dependence.

To determine whether the structural differences discussed above significantly affect the calculated excitation energies, TD-DHDFT calculations were repeated on the PBE0-, B3LYP-, and M06-2X-optimized geometries of molecules **1**, **2**, and **7**. Four representative DH functionals were selected for this analysis: B2PLYP and ωB88PP86, which provide comparatively close overall agreement with the experimental absorption maxima, and DSD-BLYP and SCS-ωB2GPPLYP, which exhibit substantially larger deviations for selected bands. This selection therefore allows the geometry sensitivity to be assessed for functionals displaying markedly different performances in the main benchmark. The resulting B-, Q1-, and Q3-band excitation energies are summarized in Table 6.

Overall, the calculated excitation energies show only a weak dependence on the functional used for the ground-state geometry optimization. For molecules **1** and **2**, the results obtained from the PBE0 and M06-2X geometries are generally very similar, whereas the B3LYP geometries tend to produce slightly lower excitation energies. Nevertheless, the differences remain small: across all molecules, bands, and DH functionals examined, the total variation associated with the choice of optimized geometry does not exceed approximately 0.04 eV. For the B band of molecules **1** and **2**, for example, the variation is only about 0.03 eV, while similarly small differences are observed for the corresponding Q1 transitions. The Q3 transition of molecule **1** exhibits the largest sensitivity, with a maximum variation of approximately 0.04 eV. Interestingly, molecule **7** shows particularly small changes in its excitation energies despite the pronounced difference in the phenyl dihedral angle predicted by B3LYP relative to PBE0 and M06-2X. This indicates that, for the electronic transitions considered here, the orientation of the peripheral phenyl rings has only a limited influence on the relevant excitation energies. Most importantly, changing the ground-state optimization functional does not alter the qualitative performance of the tested DH methods: functionals showing small deviations at the PBE0 geometry remain comparatively close to experiment, whereas the functionals exhibiting large errors retain those large deviations when B3LYP- or M06-2X-optimized geometries are employed. These results therefore indicate that the principal performance trends observed in the present benchmark arise predominantly from the excited-state electronic-structure treatment rather than from the choice of PBE0 for the ground-state geometry optimization.

## 3. Materials and Methods

All quantum chemical calculations have been performed with ORCA 5.0.2 code [128,129], and cube files were generated using ORCA 5.0.2 utility program orca_plot [128]. Molecular orbital isosurface densities have been visualized using Gabedit 2.4.8 [130]. The ground state equilibrium geometries have been fully optimized for all 13 molecules without any symmetry restriction with DFT using PBE0 functional [131] and the Ahlrichs def2-TZVP [132] basis set. Equilibrium geometries have also been optimized for molecules **1**, **2**, and **7** with B3LYP [133] and M06-2X [134] functionals with the same basis set. Frequency calculations were performed for all ground state optimized geometries at the same level of theory. We have obtained real frequency values for all optimized structures.

The lowest 20 singlet-singlet vertical electronic excitation on the optimized ground state geometries were calculated by means of TD-DFT and the def2-TZVP basis set. Tamm-Dancoff approximation (TDA) [101], which is set as default in ORCA, was not used and therefore all our excited state calculations are performed within the so called full-TD-DFT regime. We have assessed the impact of solvent effects in all geometry optimizations and excited state calculations using the non-equilibrium regime of the linear-response conductor-like polarizable continuum model (LR-CPCM) [135], and the same solvents used in the corresponding experimental procedures; see Table 1. All DFT and TD-DFT calculations were sped up with the resolution-of-the-identity (RI) approximation via the RIJCOSX procedure [136], which is a standard setting in ORCA that uses the RI for Coulomb integrals (RI-J) and the chain-of-spheres approximation for exchange integrals (COSX) [137]. The RI-MP2 [138] was used for all DHs, since they incorporate significant factions of second-order perturbative correlation terms. The auxiliary basis sets def2/J [139] and def2-TZVP/C [140] were used for corresponding RIJCOSX and RI-MP2 procedures, respectively. Converged self-consistent field orbitals were obtained using the “TightSCF” setting in ORCA (energy change $=10^{-8}\;E_{h}$). Finally, a multi-grid approach [141] is automatically selected in ORCA code using machine learning techniques.

In Table 7, the DH functionals tested and their composition are reported. The parameters defining each functional follow the conventional decomposition of exchange (x) and correlation (c) contributions. The coefficient $a_{x}$ denotes the fraction of exact HF exchange mixed with the semilocal exchange component, while $a_{c}$ represents the fraction of the second-order perturbative correlation-energy term. The terms $c_{c}$, $c_{o}$, and $c_{s}$, special for DSD type functionals, are scalling factors for the opposite- and same-spin contributions to the MP2 energy. In SCS/SOS framework, the correlation contribution to the excitation energy is decomposed into same-spin (SS) and opposite-spin (OS) components, which may be scaled independently. The scaling can be introduced for both the direct (U) and indirect (T) terms of the CIS(D) correction, and were optimized against a large benchmark set of excitation energies [96]. In the spin-component-scaled (SCS) variant, all four scaling parameters are fitted, whereas in the spin-opposite-scaled (SOS) variant only the opposite-spin contributions are retained. When all scaling parameters are equal, the original unscaled double-hybrid functional is recovered. For the range-separated functionals, the parameter ω controls the distance-dependent partitioning between the short- and long-range exchange contributions.

The DH functionals considered in the present work are grouped into four categories: global double hybrids (GDH), range-separated double hybrids (RSDH), spin-component- and spin-opposite-scaled global double hybrids (SCS/SOS-GDH), and spin-component- and spin-opposite-scaled range-separated double hybrids (SCS/SOS-RSDH), following classifications adopted in recent related studies [89,91]. This pragmatic grouping is introduced solely to facilitate analysis of the influence of range separation and spin-component scaling on the performance of DH functionals for the excited-state properties of porphyrins investigated herein. Nevertheless, all these functionals actually belong to the same rung of Jacob’s ladder of density functional approximations [150,151], namely the fifth rung, which incorporates a perturbative treatment of electron correlation and is generally regarded as the most advanced level within the DFT hierarchy.

## 4. Conclusions

In the present work, the vertical excitation energies of 13 porphyrins and metalloporphyrins were benchmarked using 28 double-hybrid density functionals within the full TD-DFT framework. The results reveal a pronounced dependence of functional performance on the type of porphyrinic excitation considered. For the B band, several functionals from different DH families provide close agreement with the experimental absorption maxima, with SOS-PBE-QIDH, SCS-PBE-QIDH, and B2PLYP forming the leading group. The leave-one-molecule-out analysis shows that this group remains robust upon removal of individual molecules, although the precise numerical ordering among the best-performing methods is not significant. For the Q bands, the best overall agreement is obtained mainly with unscaled GDH and selected RSDH functionals. In particular, B2PLYP provides one of the most balanced performances across all three bands, with MAEs of approximately 0.03, 0.06, and 0.07 eV for the B, Q1, and Q3 bands, respectively, while B2GPPLYP, ωB88PP86, and ωPBEPP86 also yield MAEs below 0.1 eV for all three bands.

A particularly important outcome is the strong band dependence exhibited by several SCS/SOS-RSDH functionals. Although some members of this family perform very well for the B band, their Q1- and Q3-band errors show pronounced systematic negative biases, leading to substantially larger MAEs. The signed-error distributions and calculated-versus-experimental parity analyses confirm that these deviations reflect systematic underestimation rather than merely isolated outliers or broad random scatter. These findings demonstrate that favorable performance for one class of porphyrinic excitation does not necessarily transfer to another and emphasize the importance of evaluating B and Q states separately when assessing DH functionals for porphyrinic systems.

Comparison with published high-level multireference (MRMP, IVO-MRMP, and CASPT2) vertical excitation energies provides a more nuanced interpretation of the benchmark. For several B-band transitions, the favorable DH results obtained relative to experiment are also supported by the available multireference calculations. For the Q states, however, the published high-level theoretical values themselves exhibit appreciable method dependence, and the DH functionals that most closely reproduce the experimental absorption maxima are not always those that lie closest to the multireference vertical excitation energies. Accordingly, the present statistical results should primarily be interpreted as an assessment of agreement with experimental spectral positions within the vertical approximation, rather than as an unambiguous ranking of intrinsic vertical-excitation-energy accuracy. Nevertheless, several DH approaches yield vertical excitation energies comparable to published multireference estimates at substantially lower computational cost.

Finally, the geometry-sensitivity analysis shows that changing the ground-state optimization functional from PBE0 to B3LYP or M06-2X alters the calculated excitation energies by no more than approximately 0.04 eV for the representative systems examined and does not modify the qualitative predictions of the tested DH approaches. Thus, the principal performance trends identified in the present benchmark arise predominantly from the excited-state electronic-structure treatment rather than from the choice of ground-state geometry. Future work may extend this analysis to emission and fluorescence properties, and larger and more strongly conjugated porphyrinic architectures relevant to chemical biology and materials science. The excited states of non-covalently bound systems also represent an interesting direction for future investigation [152,153].

## Figures and Tables

**Table 1:** Experimental maximum absorptions of B, Q1 and Q3 bands (eV) of porphyrins used as reference herein, and the corresponding solvents used in the experiments [105,106].

Molecule	B Band	Q1 Band	Q3 Band	Solvent
**1**	3.129	2.537	2.203	Toluene
**2**	3.084	2.309		Toluene
**3**	3.107	2.357		Toluene
**4**	3.100	2.487	2.181	Benzene
**5**	3.026	2.277		Toluene
**6**	3.067	2.327		Toluene
**7**	2.957	2.411	2.095	Toluene
**8**	2.910	2.198		Toluene
**9**	2.933	2.258		Toluene
**10**	2.947	2.409	2.097	Toluene
**11**	2.818	2.127	1.701	${\mathrm{CHCl}}_{3}$
**12**	2.890, 2.995	2.046	1.873	DMF
**13**	2.870	1.974		Pyridine

**Table 2:** The mean absolute error (MAE), error range ($\Delta_{\mathrm{err}}$) and standard deviation of the absolute errors (SDAE) of porphyrin’s vertical excitation B, Q1, and Q3 bands in eV, with def2-TZVP/CPCM.

	B Band			Q1 Band			Q3 Band		
Functional	MAE	$\Delta_{\mathrm{err}}$	SDAE	MAE	$\Delta_{\mathrm{err}}$	SDAE	MAE	$\Delta_{\mathrm{err}}$	SDAE
**GDH**									
PBE0-DH	0.170	0.172	0.044	0.071	0.245	0.038	0.114	0.168	0.071
mPW2PLYP	0.053	0.170	0.036	0.062	0.235	0.046	0.072	0.180	0.075
B2PLYP	0.026	0.160	0.027	0.062	0.230	0.049	0.068	0.180	0.076
PBE-QIDH	0.105	0.180	0.046	0.073	0.226	0.054	0.072	0.186	0.077
B2GPPLYP	0.033	0.170	0.037	0.078	0.220	0.057	0.067	0.190	0.076
PBE0-2	0.043	0.181	0.043	0.139	0.201	0.059	0.081	0.202	0.075
**RSDH**									
RSX-0DH	0.346	0.256	0.067	0.200	0.307	0.093	0.176	0.245	0.108
ωB2PLYP	0.211	0.210	0.059	0.126	0.258	0.067	0.138	0.210	0.097
RSX-QIDH	0.224	0.212	0.059	0.089	0.249	0.047	0.110	0.213	0.072
ωB2GPPLYP	0.172	0.210	0.057	0.081	0.240	0.040	0.108	0.210	0.075
ωB88PP86	0.096	0.192	0.049	0.041	0.232	0.052	0.074	0.208	0.027
ωPBEPP86	0.067	0.188	0.047	0.070	0.219	0.059	0.059	0.209	0.047
**SCS/SOS-GDH**									
DSD-BLYP	0.482	0.190	0.055	0.462	0.420	0.128	0.215	0.240	0.090
DSD-PBEP86	0.522	0.210	0.057	0.435	0.380	0.118	0.198	0.250	0.091
SCS/SOS-B2PLYP	0.296	0.182	0.046	0.272	0.252	0.067	0.312	0.194	0.084
SCS-B2GPPLYP	0.151	0.160	0.041	0.050	0.244	0.051	0.093	0.232	0.022
SOS-B2GPPLYP	0.105	0.152	0.039	0.088	0.241	0.032	0.123	0.234	0.077
SCS-PBE-QIDH	0.026	0.141	0.025	0.187	0.227	0.072	0.256	0.234	0.102
SOS-PBE-QIDH	0.025	0.132	0.023	0.261	0.235	0.080	0.347	0.254	0.112
**SCS/SOS-RSDH**									
SCS/SOS-ωB2PLYP	0.118	0.194	0.052	0.443	0.273	0.097	0.547	0.257	0.125
SCS-ωB2GPPLYP	0.038	0.170	0.028	0.548	0.230	0.089	0.689	0.247	0.119
SOS-ωB2GPPLYP	0.050	0.175	0.039	0.518	0.256	0.100	0.671	0.273	0.130
SCS-RSX-QIDH	0.166	0.159	0.047	0.539	0.186	0.063	0.645	0.215	0.089
SOS-RSX-QIDH	0.099	0.148	0.038	0.796	0.313	0.112	1.008	0.295	0.140
SCS-ωB88PP86	0.042	0.160	0.037	0.368	0.223	0.081	0.467	0.235	0.111
SOS-ωB88PP86	0.033	0.155	0.032	0.456	0.247	0.091	0.577	0.260	0.121
SCS-ωPBEPP86	0.141	0.143	0.041	0.553	0.208	0.073	0.667	0.234	0.102
SOS-ωPBEPP86	0.049	0.143	0.024	0.551	0.257	0.095	0.698	0.268	0.124

**Table 3:** Leave-one-molecule-out (LOO) sensitivity analysis of the B-, Q1-, and Q3-band MAE rankings for the eight DH functionals with the lowest full-set MAEs for each band. ${\mathrm{MAE}}_{\mathrm{Full}}$ denotes the MAE obtained using the full dataset for each band, whereas the ${\mathrm{MAE}}_{\mathrm{LOO}}$ range gives the minimum and maximum MAEs obtained upon successive omission of each molecule. For the B band, removal of compound **12** entails simultaneous removal of its two experimentally resolved B-band transitions. Functional ranks were determined relative to the complete set of 28 DH functionals. The corresponding full-set and LOO rank ranges are also reported. MAE values are given in eV.

Band	Functional	${\mathrm{MAE}}_{\mathrm{Full}}$	${\mathrm{MAE}}_{\mathrm{LOO}}$ Range	Full Set Rank	LOO Rank Range
**B band**	SOS-PBE-QIDH	0.025	0.021–0.027	1	1–3
	SCS-PBE-QIDH	0.026	0.021–0.027	2	2–3
	B2PLYP	0.026	0.021–0.028	3	1–3
	B2GPPLYP	0.033	0.025–0.035	4	4–6
	SOS-ωB88PP86	0.033	0.026–0.035	5	4–5
	SCS-ωB2GPPLYP	0.038	0.034–0.040	6	5–8
	SCS-ωB88PP86	0.042	0.035–0.045	7	7–8
	PBE0-2	0.043	0.034–0.045	8	6–8
**Q1 band**	ωB88PP86	0.041	0.031–0.045	1	1–1
	SCS-B2GPPLYP	0.050	0.040–0.053	2	2–2
	B2PLYP	0.062	0.051–0.066	3	3–4
	mPW2PLYP	0.062	0.051–0.067	4	3–4
	ωPBEPP86	0.070	0.058–0.074	5	5–6
	PBE0-DH	0.071	0.062–0.076	6	5–7
	PBE-QIDH	0.073	0.062–0.079	7	6–8
	B2GPPLYP	0.078	0.067–0.084	8	8–11
**Q3 band**	ωPBEPP86	0.059	0.043–0.071	1	1–3
	B2GPPLYP	0.067	0.042–0.080	2	1–4
	B2PLYP	0.068	0.043–0.079	3	2–4
	PBE-QIDH	0.072	0.048–0.086	4	4–6
	mPW2PLYP	0.072	0.048–0.083	5	4–6
	ωB88PP86	0.074	0.067–0.081	6	2–7
	PBE0-2	0.081	0.056–0.097	7	6–10
	SCS-B2GPPLYP	0.093	0.088–0.100	8	7–8

**Table 4:** Published high-level theoretical vertical excitation energies (eV) obtained with MRMP, IVO-MRMP, and CASPT2 for free-base porphin (**1**), Mg-porphin (**2**), and Zn-porphin (**3**), compared with selected DH TD-DFT vertical excitation energies obtained in the present work. Experimental absorption maxima are included for reference.

		Molecule 1				Molecule 2			Molecule 3	
	Band:	B	Q1	Q3		B	Q1		B	Q1
	State:	2 ^1^ $B_{3u}$	1 ^1^ $B_{2u}$	1 ^1^ $B_{3u}$		2 ^1^ $E_{1u}$	1 ^1^ $E_{1u}$		2 ^1^ $E_{1u}$	1 ^1^ $E_{1u}$
Method										
MRMP ^*a*^		3.10	2.55	1.63		3.07	2.00		3.21	2.11
IVO-MRMP ^*b*^		3.06	2.64	1.86		3.15	2.14		3.37	2.28
CASPT2 ^*c,d*^		3.12	2.11	1.63		2.66	1.66			
B2PLYP		3.12	2.50	2.22		3.11	2.37		3.14	2.42
B2GPPLYP		3.14	2.53	2.21		3.12	2.38		3.15	2.43
ωB88PP86		3.20	2.47	2.10		3.18	2.30		3.21	2.36
ωPBEPP86		3.17	2.52	2.13		3.15	2.34		3.18	2.40
SCS-B2GPPLYP		3.25	2.45	2.09		3.23	2.28		3.27	2.34
SOS-B2GPPLYP		3.21	2.39	2.01		3.19	2.21		3.23	2.26
SCS-ωB88PP86		3.15	2.10	1.66		3.13	1.90		3.16	1.95
SOS-ωB88PP86		3.13	2.01	1.53		3.11	1.79		3.15	1.85
Exp ^*e*^		3.13	2.54	2.20		3.08	2.31		3.11	2.36

*a*: Ref. [33], active space (8e,8o); *b*: Ref. [34], active space (16e,12o) *c*: ref. [31] for free-base porphyrin **1**, active space (14e,16o); *d*: ref. [32] for Mg porphyrin **2**, active space (14e,16o); *e*: Experimental $\lambda_{\max}$ from PhotochemCAD database refs. [105,106].

**Table 5:** Selected geometry parameters, for molecules **1**, **2**, and **7**, optimized at PBE0/def2-TZVP/CPCM, B3LYP/def2-TZVP/CPCM, and M06-2X/def2-TZVP/CPCM levels of theory. The definition of N⋯N, H⋯H, and phenyl dihedral angle are described in the text. Distances in Å and dihedral angles in degree °.

Geometry	PBE0	B3LYP	M06-2X
**1**			
N⋯N	4.032	4.063	4.021
H⋯H	2.190	2.205	2.174
**2**			
Mg–N	2.050	2.058	2.045
**7**			
N⋯N	4.049	4.076	4.044
H⋯H	2.170	2.185	2.156
Phenyl dihedral angle	73.0	89.7	71.6

**Table 6:** Vertical excitation energies (eV) obtained with selected DH functionals using ground-state geometries optimized at the PBE0/def2-TZVP/CPCM, B3LYP/def2-TZVP/CPCM, and M06-2X/def2-TZVP/CPCM levels for molecules **1**, **2**, and **7**. Experimental absorption maxima are included for reference.

Band	Opt	B2PLYP	wB88PP86	DSD-BLYP	SCS-ωB2GPPLYP	Exp.
Molecule						
**B Band**						
**1**	PBE0	3.123	3.197	2.640	3.102	3.129
	B3LYP	3.099	3.173	2.614	3.079	
	M06-2X	3.126	3.200	2.641	3.107	
**2**	PBE0	3.105	3.175	2.601	3.079	3.084
	B3LYP	3.081	3.151	2.574	3.055	
	M06-2X	3.108	3.179	2.598	3.084	
**7**	PBE0	2.941	3.020	2.496	2.906	2.957
	B3LYP	2.939	3.017	2.481	2.906	
	M06-2X	2.940	3.020	2.493	2.908	
**Q1 Band**						
**1**	PBE0	2.496	2.469	2.997	1.933	2.537
	B3LYP	2.478	2.452	2.991	1.913	
	M06-2X	2.499	2.471	2.999	1.934	
**2**	PBE0	2.367	2.299	2.671	1.708	2.309
	B3LYP	2.347	2.278	2.655	1.683	
	M06-2X	2.370	2.301	2.677	1.710	
**7**	PBE0	2.387	2.397	2.963	1.832	2.411
	B3LYP	2.396	2.401	2.989	1.831	
	M06-2X	2.390	2.403	2.965	1.835	
**Q3 Band**						
**1**	PBE0	2.219	2.104	2.338	1.436	2.203
	B3LYP	2.193	2.076	2.311	1.401	
	M06-2X	2.225	2.109	2.349	1.441	
**7**	PBE0	2.109	2.030	2.281	1.324	2.095
	B3LYP	2.105	2.019	2.280	1.300	
	M06-2X	2.115	2.036	2.290	1.329	

**Table 7:** Composition of DH density functionals tested in the present study.

Functional	$a_{x}$	$a_{c}$	ω	x	c	Year	Ref.
		$c_{c}$/$c_{o}$/$c_{s}$					
		$c_{T}^{\mathrm{SS}}$/$c_{T}^{\mathrm{OS}}$/$c_{U}^{\mathrm{SS}}$/$c_{U}^{\mathrm{OS}}$					
**GDH**							
PBE0-DH	0.50	0.125		PBE	PBE	2011	[142]
mPW2PLYP	0.55	0.25		mPW	LYP	2006	[143]
B2PLYP	0.53	0.27		B88	LYP	2006	[73]
PBE-QIDH	0.693	0.333		PBE	PBE	2014	[144]
B2GPPLYP	0.65	0.36		B88	LYP	2008	[145]
PBE0-2	0.794	0.50		PBE	PBE	2012	[146]
**RSDH**							
RSX-0DH	0.50	0.125	0.33	PBE	PBE	2019	[147]
ωB2PLYP	0.53	0.27	0.30	ωB88	LYP	2019	[79]
RSX-QIDH	0.693	0.333	0.27	PBE	PBE	2019	[147]
ωB2GPPLYP	0.65	0.36	0.27	ωB88	LYP	2019	[79]
ωB88PP86	0.65	0.42	0.20	ωB88	P86	2021	[96]
ωPBEPP86	0.70	0.48	0.18	ωPBE	P86	2021	[96]
**SCS/SOS-GDH**							
DSD-BLYP	0.69	0.54/0.46/0.37		B88	LYP	2010	[148]
DSD-PBEP86	0.70	0.43/0.53/0.25		PBE	P86	2011	[149]
SCS/SOS-B2PLYP ^1^	0.53	0.000/0.334/0.000/0.283		B88	LYP	2021	[96]
SCS-B2GPPLYP	0.65	0.018/0.475/0.000/0.468		B88	LYP	2021	[96]
SOS-B2GPPLYP	0.65	0.000/0.489/0.000/0.484		B88	LYP	2021	[96]
SCS-PBE-QIDH	0.693	0.070/0.515/0.096/0.524		PBE	PBE	2021	[96]
SOS-PBE-QIDH	0.693	0.000/0.547/0.000/0.573		PBE	PBE	2021	[96]
**SCS/SOS-RSDH**							
SCS/SOS-ωB2PLYP ^1^	0.53	0.000/0.433/0.000/0.460	0.30	ωB88	LYP	2021	[96]
SCS-ωB2GPPLYP	0.65	0.000/0.571/0.135/0.558	0.27	ωB88	LYP	2021	[96]
SOS-ωB2GPPLYP	0.65	0.000/0.570/0.000/0.610	0.27	ωB88	LYP	2021	[96]
SCS-RSX-QIDH	0.693	0.174/0.541/0.586/0.424	0.27	ωPBE	PBE	2021	[96]
SOS-RSX-QIDH	0.693	0.000/0.632/0.000/0.714	0.27	ωPBE	PBE	2021	[96]
SCS-ωB88PP86	0.65	0.000/0.557/0.092/0.545	0.20	ωB88	P86	2021	[96]
SOS-ωB88PP86	0.65	0.000/0.559/0.000/0.599	0.20	ωB88	P86	2021	[96]
SCS-ωPBEPP86	0.70	0.000/0.610/0.297/0.540	0.18	ωPBE	P86	2021	[96]
SOS-ωPBEPP86	0.70	0.000/0.613/0.000/0.669	0.18	ωPBE	P86	2021	[96]

List of abbreviations and symbols: x: exchange functional; c: correlation functional; **GDH**: global double hybrid; **RSDH**: range separated double hybrid; **SCS/SOS**: spin-component scaling/spinopposite scaling; *a*_x_, *a*_c_, *c*_c_, *c*_o_, *c*_s_, $c_{U}^{\mathrm{SS}}$, $c_{u}^{\mathrm{OS}}$, $c_{T}^{\mathrm{SS}}$, $c_{T}^{\mathrm{OS}}$, and *ω*: defined in the text; ***Notes***: ^1^: The SCS fitting procedure led directly to the SOS parameters [96].

## Data Availability

The raw data supporting the conclusions of this article will be made available by the corresponding author on request.

## Supplementary Materials

The following supporting information can be downloaded at: https://www.mdpi.com/article/10.3390/molecules31173027/s1.

## Abbreviations

The following abbreviations are used in this manuscript:

CPCM	Conductor-like Polarizable Continuum Model
DFT	Density Functional Theory
DH	Double Hybrid
GDH	Global Double Hybrid
MAE	Mean Absolute Error
RSDH	Range-Separated Double Hybrid
SCS	Spin-Component Scaled
SDAE	Standard Deviation of Absolute Errors
SOS	Spin-Opposite Scaled
TD	Time-Dependent
