# Supplementary material for: Assessment of Double-Hybrid Functionals for the Vertical Excitation Energies of Porphyrins
Source: Molecules. 2026 Aug 28;31(17):3027. doi: 10.3390/molecules31173027 (PMC13567032; doi:10.3390/molecules31173027)
Supplement: Supplementary file 1 [file molecules-31-03027-s001.zip › molecules-4412289-supplementary.pdf]

Supplementary Material for:

Assessment of Double-Hybrid Functionals for the Vertical Excitation Energies of Porphyrins

Wissam Helal,[1,*] Rahma Sarayra,[2] Ahmad S. Barham,[1] Ali Elrashidi[3,4]

[1] *Department of Chemistry, The University of Jordan, Amman 11942, Jordan.*
[2] *International Independent Schools, Khalda Branch, P.O.Box 499, Amman 11732, Jordan.*
[3] *Electrical Engineering Department, College of Engineering, University of Business and Technology, Jeddah 23435, Saudi Arabia.*
[4] *Engineering Mathematics Department, Faculty of Engineering, Alexandria University, Alexandria 21544, Egypt.*

* Correspondence: wissam.helal@ju.edu.jo

Contents

S1 Definitions of Statistical Measures S9
S2 Cartesian Coordinates of Molecules **1–13** S11
S3 Calculated Vertical Excitaion Energies of B Band S38
S4 Calculated Vertical Excitaion Energies of Q1 Band S51
S5 Calculated Vertical Excitaion Energies of Q3 Band S64
S6 Molecular Orbital Plots S70
S7 Comparison of Calculated and Experimnetal Excitaion Energies: The B Band S73
S8 Comparison of Calculated and Experimnetal Excitaion Energies: The Q1 Band S77

S9 Comparison of Calculated and Experimnetal Excitaion Energies: The Q3 Band . . . S81

S10 Summary of Statistical Measures Compared with Experiment . . . S83

S11 Leave-One-molecule-Out (LOO) Sensitivity Analysis . . . S86

S12 Computational Cost of DH Functionals . . . S89

List of Tables

Table S1 Calculated Cartesian coordinates of ground state optimized geometries (in Å) for molecules (**1** – **13**), Determined at the BPE0/def2-TZVP level in solvent with CPCM solvation model. . . . S11

Table S2 Calculated Cartesian coordinates of ground state optimized geometries (in Å) for molecules (**1**, **2**, and **7**), Determined at the B3LYP/def2-TZVP level in solvent with CPCM solvation model. . . . S30

Table S3 Calculated Cartesian coordinates of ground state optimized geometries (in Å) for molecules (**1**, **2**, and **7**), Determined at the M06-2X/def2-TZVP level in solvent with CPCM solvation model. . . . S34

Table S4 Calculated Vertical Excitaion Energies (EE) in nm and eV, Oscillator Strength (f), and their MO Designation and Corresponding Coefficients, of the B Band of Molecule **1**, based on BPE0/def2-TZVP/CPCM optimized geometry. . . . S38

Table S5 Calculated Vertical Excitaion Energies (EE) in nm and eV, Oscillator Strength (f), and their MO Designation and Corresponding Coefficients, of the B Band of Molecule **2**, based on BPE0/def2-TZVP/CPCM optimized geometry. . . . S39

Table S6 Calculated Vertical Excitaion Energies (EE) in nm and eV, Oscillator Strength (f), and their MO Designation and Corresponding Coefficients, of the B Band of Molecule **3**, based on BPE0/def2-TZVP/CPCM optimized geometry. . . . S40

Table S7 Calculated Vertical Excitaion Energies (EE) in nm and eV, Oscillator Strength (f), and their MO Designation and Corresponding Coefficients, of the B Band of Molecule **4**, based on BPE0/def2-TZVP/CPCM optimized geometry. . . . S41

Table S8 Calculated Vertical Excitaion Energies (EE) in nm and eV, Oscillator Strength (f), and their MO Designation and Corresponding Coefficients, of the B Band of Molecule **5**, based on BPE0/def2-TZVP/CPCM optimized geometry. S42

Table S9 Calculated Vertical Excitaion Energies (EE) in nm and eV, Oscillator Strength (f), and their MO Designation and Corresponding Coefficients, of the B Band of Molecule **6**, based on BPE0/def2-TZVP/CPCM optimized geometry. S43

Table S10 Calculated Vertical Excitaion Energies (EE) in nm and eV, Oscillator Strength (f), and their MO Designation and Corresponding Coefficients, of the B Band of Molecule **7**, based on BPE0/def2-TZVP/CPCM optimized geometry. S44

Table S11 Calculated Vertical Excitaion Energies (EE) in nm and eV, Oscillator Strength (f), and their MO Designation and Corresponding Coefficients, of the B Band of Molecule **8**, based on BPE0/def2-TZVP/CPCM optimized geometry. S45

Table S12 Calculated Vertical Excitaion Energies (EE) in nm and eV, Oscillator Strength (f), and their MO Designation and Corresponding Coefficients, of the B Band of Molecule **9**, based on BPE0/def2-TZVP/CPCM optimized geometry. S46

Table S13 Calculated Vertical Excitaion Energies (EE) in nm and eV, Oscillator Strength (f), and their MO Designation and Corresponding Coefficients, of the B Band of Molecule **10**, based on BPE0/def2-TZVP/CPCM optimized geometry. S47

Table S14 Calculated Vertical Excitaion Energies (EE) in nm and eV, Oscillator Strength (f), and their MO Designation and Corresponding Coefficients, of the B Band of Molecule **11**, based on BPE0/def2-TZVP/CPCM optimized geometry. S48

Table S15 Calculated Vertical Excitaion Energies (EE) in nm and eV, Oscillator Strength (f), and their MO Designation and Corresponding Coefficients, of the B Band of Molecule **12**, based on BPE0/def2-TZVP/CPCM optimized geometry. S49

Table S16 Calculated Vertical Excitaion Energies (EE) in nm and eV, Oscillator Strength (f), and their MO Designation and Corresponding Coefficients, of the B Band of Molecule **13**, based on BPE0/def2-TZVP/CPCM optimized geometry. S50

Table S17 Calculated Vertical Excitaion Energies (EE) in nm and eV, Oscillator Strength (f), and their MO Designation and Corresponding Coefficients, of the Q1 Band of Molecule **1**, based on BPE0/def2-TZVP/CPCM optimized geometry. S51

Table S18 Calculated Vertical Excitaion Energies (EE) in nm and eV, Oscillator Strength (f), and their MO Designation and Corresponding Coefficients, of the Q1 Band of Molecule **2**, based on BPE0/def2-TZVP/CPCM optimized geometry. S52

Table S19 Calculated Vertical Excitaion Energies (EE) in nm and eV, Oscillator Strength (f), and their MO Designation and Corresponding Coefficients, of the Q1 Band of Molecule **3**, based on BPE0/def2-TZVP/CPCM optimized geometry. S53

Table S20 Calculated Vertical Excitaion Energies (EE) in nm and eV, Oscillator Strength (f), and their MO Designation and Corresponding Coefficients, of the Q1 Band of Molecule **4**, based on BPE0/def2-TZVP/CPCM optimized geometry. S54

Table S21 Calculated Vertical Excitaion Energies (EE) in nm and eV, Oscillator Strength (f), and their MO Designation and Corresponding Coefficients, of the Q1 Band of Molecule **5**, based on BPE0/def2-TZVP/CPCM optimized geometry. S55

Table S22 Calculated Vertical Excitaion Energies (EE) in nm and eV, Oscillator Strength (f), and their MO Designation and Corresponding Coefficients, of the Q1 Band of Molecule **6**, based on BPE0/def2-TZVP/CPCM optimized geometry. S56

Table S23 Calculated Vertical Excitaion Energies (EE) in nm and eV, Oscillator Strength (f), and their MO Designation and Corresponding Coefficients, of the Q1 Band of Molecule **7**, based on BPE0/def2-TZVP/CPCM optimized geometry. S57

Table S24 Calculated Vertical Excitaion Energies (EE) in nm and eV, Oscillator Strength (f), and their MO Designation and Corresponding Coefficients, of the Q1 Band of Molecule **8**, based on BPE0/def2-TZVP/CPCM optimized geometry. S58

Table S25 Calculated Vertical Excitaion Energies (EE) in nm and eV, Oscillator Strength (f), and their MO Designation and Corresponding Coefficients, of the Q1 Band of Molecule **9**, based on BPE0/def2-TZVP/CPCM optimized geometry. S59

Table S26 Calculated Vertical Excitaion Energies (EE) in nm and eV, Oscillator Strength (f), and their MO Designation and Corresponding Coefficients, of the Q1 Band of Molecule **10**, based on BPE0/def2-TZVP/CPCM optimized geometry. S60

Table S27 Calculated Vertical Excitaion Energies (EE) in nm and eV, Oscillator Strength (f), and their MO Designation and Corresponding Coefficients, of the Q1 Band of Molecule **11**, based on BPE0/def2-TZVP/CPCM optimized geometry. S61

Table S28 Calculated Vertical Excitaion Energies (EE) in nm and eV, Oscillator Strength (f), and their MO Designation and Corresponding Coefficients, of the Q1 Band of Molecule **12**, based on BPE0/def2-TZVP/CPCM optimized geometry. S62

Table S29 Calculated Vertical Excitaion Energies (EE) in nm and eV, Oscillator Strength (f), and their MO Designation and Corresponding Coefficients, of the Q1 Band of Molecule **13**, based on BPE0/def2-TZVP/CPCM optimized geometry. S63

Table S30 Calculated Vertical Excitaion Energies (EE) in nm and eV, Oscillator Strength (f), and their MO Designation and Corresponding Coefficients, of the Q3 Band of Molecule **1**, based on BPE0/def2-TZVP/CPCM optimized geometry. S64

Table S31 Calculated Vertical Excitaion Energies (EE) in nm and eV, Oscillator Strength (f), and their MO Designation and Corresponding Coefficients, of the Q3 Band of Molecule **4**, based on BPE0/def2-TZVP/CPCM optimized geometry. S65

Table S32 Calculated Vertical Excitaion Energies (EE) in nm and eV, Oscillator Strength (f), and their MO Designation and Corresponding Coefficients, of the Q3 Band of Molecule **7**, based on BPE0/def2-TZVP/CPCM optimized geometry. S66

Table S33 Calculated Vertical Excitaion Energies (EE) in nm and eV, Oscillator Strength (f), and their MO Designation and Corresponding Coefficients, of the Q3 Band of Molecule **10**, based on BPE0/def2-TZVP/CPCM optimized geometry. S67

Table S34 Calculated Vertical Excitaion Energies (EE) in nm and eV, Oscillator Strength (f), and their MO Designation and Corresponding Coefficients, of the Q3 Band of Molecule **11**, based on BPE0/def2-TZVP/CPCM optimized geometry. S68

Table S35 Calculated Vertical Excitaion Energies (EE) in nm and eV, Oscillator Strength (f), and their MO Designation and Corresponding Coefficients, of the Q3 Band of Molecule **12**, based on BPE0/def2-TZVP/CPCM optimized geometry. S69

Table S36 Vertical Excitation Energies (eV) of the B Band (Soret) Using GDH Density Functionals with the def2-TZVP Basis Set and CPCM solvation Model. S73

Table S37 Vertical Excitation Energies (eV) of the B Band (Soret) Using RSDH Density Functionals with the def2-TZVP Basis Set and CPCM solvation Model. S74

Table S38 Vertical Excitation Energies (eV) of the B Band (Soret) Using SCS/SOS-GDH Density Functionals with the def2-TZVP Basis Set and CPCM solvation Model. S75

Table S39 Vertical Excitation Energies (eV) of the B Band (Soret) Using SCS/SOS-RSDH Density Functionals with the def2-TZVP Basis Set and CPCM solvation Model. S76

Table S40 Vertical Excitation Energies (eV) of the Q1 Band Using GDH Density Functionals with the def2-TZVP Basis Set and CPCM solvation Model.S77

Table S41 Vertical Excitation Energies (eV) of the Q1 Band Using RSDH Density Functionals with the def2-TZVP Basis Set and CPCM solvation Model.S78

Table S42 Vertical Excitation Energies (eV) of the Q1 Band Using SCS/SOS-GDH Density Functionals with the def2-TZVP Basis Set and CPCM solvation Model. S79

Table S43 Vertical Excitation Energies (eV) of the Q1 Band Using SCS/SOS-RSDH Density Functionals with the def2-TZVP Basis Set and CPCM solvation Model. S80

Table S44 Vertical Excitation Energies (eV) of the Q3 Band Using GDH Density Functionals with the def2-TZVP Basis Set and CPCM solvation Model.S81

Table S45 Vertical Excitation Energies (eV) of the Q3 Band Using RSDH Density Functionals with the def2-TZVP Basis Set and CPCM solvation Model.S81

Table S46 Vertical Excitation Energies (eV) of the Q3 Band Using SCS/SOS-GDH Density Functionals with the def2-TZVP Basis Set and CPCM solvation Model. S82

Table S47 Vertical Excitation Energies (eV) of the Q3 Band Using SCS/SOS-RSDH Density Functionals with the def2-TZVP Basis Set and CPCM solvation Model. S82

Table S48 The Mean Absolute Error (MAE), Mean Signed Error (MSE), Maximum Error (Max), Minimum Error (Min), Error Range (Δ_{err}), Standard Deviation of the Absolute Errors (SDAE), and squared Pearson correlation coefficient (R^2) of the Vertical Excitation Energies in eV of the B band with the def2-TZVP Basis Set of all Molecules **1–13**. S83

Table S49 The Mean Absolute Error (MAE), Mean Signed Error (MSE), Maximum Error (Max), Minimum Error (Min), Error Range (Δ_{err}), Standard Deviation of the Absolute Errors (SDAE), and squared Pearson correlation coefficient (R^2) of the Vertical Excitation Energies in eV of the Q1 band with the def2-TZVP Basis Set of all Molecules **1–13**. S84

Table S50 The Mean Absolute Error (MAE), Mean Signed Error (MSE), Maximum Error (Max), Minimum Error (Min), Error Range (Δ_{err}), Standard Deviation of the Absolute Errors (SDAE), and squared Pearson correlation coefficient (R^2) of the Vertical Excitation Energies in eV of the Q3 band with the def2-TZVP Basis Set of Molecules **1**, **4**, **7**, **10**, **11**, and **12**. . . . S85

Table S51 Leave-one-molecule-out (LOO) sensitivity analysis of the B-band for the full set of DH functionals. MAE_{Full} is denoted by MAE while MAE_{LOO} is denoted by LOO for simplicity. The MAE obtained using all porphyrin transitions ($N = 14$), MAE and LOO values values are given in eV. . . . S86

Table S52 Leave-one-molecule-out (LOO) sensitivity analysis of the Q1-band for the full set of DH functionals. MAE_{Full} is denoted by MAE while MAE_{LOO} is denoted by LOO for simplicity. The MAE obtained using all porphyrin molecules ($N = 13$), MAE and LOO values values are given in eV. S87

Table S53 Leave-one-molecule-out (LOO) sensitivity analysis of the Q3-band for the full set of DH functionals. MAE_{Full} is denoted by MAE while MAE_{LOO} is denoted by LOO for simplicity. The MAE obtained using all six free-base porphyrins ($N = 6$), MAE and LOO values values are given in eV. S88

Table S54 Computation times (in minutes) for TD-DFT excited-state calculations, with def2-TZVP basis set, of molecule **1** (38 atoms, 828 basis functions), using various DH functionals. All calculations were performed using a CPU with 8 cores in parallel, 4.3 GHz clock speed and 64 GB of RAM. S89

List of Figures

Figure S1 Frontier MOs (HOMO-1, HOMO, LUOM and LUMO+1) involved in the main transitions of porphyrins **1–5**. Isosurface value 0.02. . . . . . S70

Figure S2 Frontier MOs (HOMO-1, HOMO, LUOM and LUMO+1) involved in the main transitions of porphyrins **6–9**. Isosurface value 0.02. . . . . . S71

Figure S3 Frontier MOs (HOMO-1, HOMO, LUOM and LUMO+1) involved in the main transitions of porphyrins **10–13**. Isosurface value 0.02. . . . S72

S1 Definitions of Statistical Measures

The statistical measures used in the present work are defined below. For each transition, the signed error is defined as

$$e_i = E_i^{\mathrm{calc}} - E_i^{\mathrm{exp}}, \tag{S1}$$

where E_i^{calc} and E_i^{exp} are the calculated excitation energy and corresponding experimental absorption maximum, respectively, and N is the number of transitions included in the dataset.

1. The mean absolute error (MAE) measures the average magnitude of the deviations from experiment, irrespective of their sign:

$$\mathrm{MAE} = \frac{1}{N}\sum_{i=1}^{N} |e_i|. \tag{S2}$$

2. The mean signed error (MSE) measures the average signed deviation and therefore provides an indication of systematic overestimation or underestimation:

$$\mathrm{MSE} = \frac{1}{N}\sum_{i=1}^{N} e_i. \tag{S3}$$

   Positive MSE values indicate an overall overestimation of the experimental excitation energies, whereas negative values indicate an overall underestimation.

3. The standard deviation of the absolute errors (SDAE) measures the dispersion of the individual absolute errors around their mean value (MAE):

$$\mathrm{SDAE} = \sqrt{\frac{1}{N-1}\sum_{i=1}^{N} (|e_i| - \mathrm{MAE})^2}. \tag{S4}$$

   A smaller SDAE therefore indicates less variation in the magnitudes of the individual errors across the benchmark set.

4. The coefficient of determination (R^2) values reported herein correspond to the square of the Pearson linear correlation coefficient between the calculated and experimental excitation energies:

$$R^2 = \frac{\left[\sum_{i=1}^{N}\left(E_i^{\mathrm{calc}} - \overline{E}^{\mathrm{calc}}\right)\left(E_i^{\mathrm{exp}} - \overline{E}^{\mathrm{exp}}\right)\right]^2}{\left[\sum_{i=1}^{N}\left(E_i^{\mathrm{calc}} - \overline{E}^{\mathrm{calc}}\right)^2\right]\left[\sum_{i=1}^{N}\left(E_i^{\mathrm{exp}} - \overline{E}^{\mathrm{exp}}\right)^2\right]}. \tag{S5}$$

Here, $\overline{E}^{\text{calc}}$ and $\overline{E}^{\text{exp}}$ denote the mean calculated and experimental excitation energies, respectively. The R^2 value measures how well the variations in the calculated excitation energies correlate with the experimental trends; it does not by itself measure proximity to the ideal $E^{\text{calc}} = E^{\text{exp}}$ relationship.

5. The maximum error (Max) is defined as the largest signed error in the dataset:

$$\text{Max} = \max_i(e_i). \tag{S6}$$

6. The minimum error (Min) is defined as the smallest signed error in the dataset:

$$\text{Min} = \min_i(e_i). \tag{S7}$$

7. The error range, also referred to as the error span (Δ_{err}), is defined as the difference between the maximum and minimum signed errors:

$$\Delta_{\text{err}} = \text{Max} - \text{Min}. \tag{S8}$$

It therefore represents the total span of the signed-error distribution.

8. To assess the sensitivity of the MAE values to the composition of the benchmark set, a leave-one-molecule-out analysis (MAE_{LOO}) was performed. The MAE was recalculated after successively removing each molecule:

$$\text{MAE}_{(-m)} = \frac{1}{N_{(-m)}} \sum_{j \in D \setminus m} |e_j|, \tag{S9}$$

where m denotes the omitted molecule, $D \setminus m$ represents the dataset after removal of all transitions belonging to that molecule, and $N_{(-m)}$ is the number of remaining transitions. For the B band, removal of molecule **12** therefore entails simultaneous removal of its two experimentally resolved B-band transitions.

S2 Cartesian Coordinates of Molecules 1–13

Table S1: Calculated Cartesian coordinates of ground state optimized geometries (in Å) for molecules (**1** – **13**), Determined at the BPE0/def2-TZVP level in solvent with CPCM solvation model.

**Molecule 1**	X	Y	Z
N	0.23696	3.05895	-0.02711
N	2.38260	1.08423	0.01807
N	-1.64161	0.83107	0.02268
N	0.50402	-1.14358	0.06798
C	1.30610	3.90140	-0.04076
C	-0.93045	3.75954	-0.03861
H	0.30081	2.04979	-0.01361
C	-0.57919	5.14268	-0.06202
C	0.78293	5.22905	-0.06329
C	2.63753	3.51531	-0.03201
C	3.13075	2.21285	-0.00484
C	4.54728	1.89941	0.00284
C	4.63370	0.55208	0.03046
C	3.26871	0.06093	0.04005
H	3.36582	4.31796	-0.04651
C	2.94402	-1.29364	0.06824
C	1.67151	-1.84365	0.07942
H	3.76722	-1.99858	0.08152
C	-0.56526	-1.98555	0.08147
H	0.44013	-0.13440	0.05435
C	1.32016	-3.22690	0.10241
C	-0.04195	-3.31328	0.10420
C	-1.89682	-1.59971	0.07268
C	-2.39005	-0.29716	0.04548
C	-2.52747	1.85480	0.00068
C	-2.20279	3.20928	-0.02746
C	-3.89240	1.36365	0.00988
C	-3.80664	0.01629	0.03803
H	-2.62461	-2.40279	0.08725
H	-3.02645	3.91372	-0.04083
H	-4.77927	1.98148	-0.00377
H	-4.60746	-0.70955	0.05240
H	-0.64764	-4.20698	0.11849
H	2.03405	-4.03682	0.11504
H	5.52051	-0.06576	0.04385
H	1.39005	6.12177	-0.07743
H	5.34814	2.62526	-0.01140
H	-1.29435	5.95147	-0.07491

**Molecule 2**	X	Y	Z
N	0.93896	3.28692	0.36899
N	2.80576	1.07032	0.32414

N	-1.27591	1.42970	0.16467
N	0.59069	-0.78845	0.11954
Mg	0.76500	1.24848	0.24432
C	2.09813	3.99505	0.46274
C	-0.08180	4.18772	0.37558
C	0.44979	5.52087	0.47770
C	1.80169	5.40141	0.53165
C	3.37608	3.44089	0.48902
C	3.70398	2.08837	0.42530
C	3.51685	-0.09005	0.29141
C	4.92326	0.20248	0.37472
C	5.03920	1.55313	0.45776
C	2.96473	-1.36535	0.19315
C	1.61195	-1.68874	0.11324
C	-0.56882	-1.49601	0.02577
C	1.07989	-3.02168	0.01101
C	-0.27187	-2.90226	-0.04278
C	-1.84637	-0.94098	-0.00060
C	-2.17378	0.41167	0.06317
C	-1.98732	2.59012	0.19728
C	-1.43482	3.86522	0.29569
C	-3.50919	0.94625	0.03039
C	-3.39367	2.29691	0.11357
H	2.53857	6.18786	0.61071
H	-0.14209	6.42460	0.50392
H	-4.41228	0.35785	-0.04695
H	-4.18363	3.03429	0.11760
H	5.71279	-0.53534	0.37050
H	5.94267	2.14103	0.53479
H	1.66969	-3.92680	-0.01538
H	-1.00694	-3.69045	-0.12177
H	-2.13234	4.69529	0.31128
H	4.20402	4.13667	0.56788
H	3.66247	-2.19522	0.17764
H	-2.67453	-1.63647	-0.07961

**Molecule 3**	X	Y	Z
N	0.17892	2.03527	-0.00005
N	-2.03562	0.17900	-0.00030
N	2.03569	-0.17872	0.00037
N	-0.17883	-2.03514	0.00012
C	-0.84017	2.93698	-0.00032
C	1.34075	2.74417	0.00007
C	1.04743	4.15278	-0.00003
C	-0.30447	4.27227	-0.00043
C	-2.19308	2.61700	-0.00054
C	-2.74445	1.34082	-0.00056
C	-2.93728	-0.84009	-0.00045
C	-4.27256	-0.30439	-0.00084
C	-4.15305	1.04754	-0.00089

C	-2.61700	-2.19293	-0.00029
C	-1.34069	-2.74403	-0.00001
C	0.84027	-2.93685	0.00036
C	-1.04737	-4.15259	-0.00003
C	0.30457	-4.27209	0.00057
C	2.19317	-2.61679	0.00059
C	2.74449	-1.34058	0.00062
C	2.93738	0.84031	0.00053
C	2.61708	2.19315	0.00036
C	4.15311	-1.04734	0.00098
C	4.27266	0.30459	0.00088
H	-0.89523	5.17711	-0.00069
H	1.78767	4.94004	0.00006
H	4.94042	-1.78753	0.00125
H	5.17757	0.89524	0.00107
H	-5.17746	-0.89506	-0.00104
H	-4.94038	1.78771	-0.00114
H	-1.78751	-4.93994	-0.00022
H	0.89522	-5.17700	0.00090
H	3.44773	2.88991	0.00050
H	-2.88954	3.44791	-0.00077
H	-3.44769	-2.88964	-0.00046
H	2.88969	-3.44764	0.00082
Zn	-0.00010	-0.00034	0.00005

**Molecule 4**	X	Y	Z
N	-0.20463	2.10115	-0.02740
N	2.01025	0.20272	-0.02416
N	-2.01244	-0.19190	0.00090
N	0.20235	-2.09048	0.00518
C	0.83219	2.98149	-0.03852
C	-1.39432	2.76066	-0.01786
H	-0.10426	1.09515	-0.02930
C	-1.09986	4.16496	-0.02329
C	0.26794	4.30088	-0.03694
C	2.17450	2.63645	-0.03894
C	2.72075	1.35481	-0.02959
C	4.15682	1.09620	-0.03296
C	4.28965	-0.25631	-0.03265
C	2.93102	-0.78905	-0.02420
H	2.87269	3.46482	-0.04602
C	2.64225	-2.15196	-0.00562
C	1.39214	-2.74994	0.00924
H	3.48645	-2.83105	-0.00063
C	-0.83443	-2.97103	0.01511
H	0.10175	-1.08452	0.00070
C	1.09782	-4.15427	0.02239
C	-0.26997	-4.29031	0.02498
C	-2.17653	-2.62582	0.02374
C	-2.72261	-1.34396	0.02032

C	-2.93350	0.79968	-0.00447
C	-2.64458	2.16267	-0.01013
C	-4.29190	0.26683	0.01011
C	-4.15864	-1.08556	0.02910
H	-2.87523	-3.45372	0.03495
H	-3.48866	2.84190	-0.00501
C	5.23387	2.12872	-0.00243
C	5.51704	2.66204	1.40171
H	4.96668	2.96308	-0.65921
H	6.15197	1.70311	-0.41686
H	6.30464	3.41953	1.38041
H	4.62417	3.11332	1.84047
H	5.83875	1.85587	2.06516
C	5.54742	-1.05945	-0.06637
C	5.91780	-1.54504	-1.46748
H	5.45309	-1.92131	0.60216
H	6.36824	-0.45811	0.33417
H	6.84121	-2.12917	-1.44655
H	5.12881	-2.17154	-1.88976
H	6.06468	-0.69946	-2.14352
C	1.05354	5.56922	-0.07815
C	1.46669	5.97409	-1.49240
H	1.94643	5.46729	0.54587
H	0.46153	6.37132	0.37027
H	2.03568	6.90670	-1.47871
H	2.08684	5.20351	-1.95578
H	0.58935	6.11969	-2.12688
C	-2.12058	5.25242	0.02206
C	-2.58076	5.58134	1.44151
H	-2.98658	4.96842	-0.58340
H	-1.70820	6.15135	-0.44352
H	-3.33093	6.37574	1.43272
H	-3.01876	4.70504	1.92451
H	-1.74098	5.91343	2.05629
C	2.11907	-5.24121	0.06684
C	2.61079	-5.54260	1.48193
H	2.97135	-4.97014	-0.56366
H	1.69587	-6.14890	-0.37108
H	3.36055	-6.33737	1.47177
H	3.05964	-4.65757	1.93832
H	1.78500	-5.86254	2.12159
C	-1.05619	-5.55878	0.00544
C	-1.48135	-5.98035	-1.40032
H	-1.94364	-5.44872	0.63577
H	-0.46080	-6.35578	0.45842
H	-2.05156	-6.91185	-1.37046
H	-2.10419	-5.21459	-1.86805
H	-0.60934	-6.13505	-2.03996
C	-5.23545	-2.11712	0.08767

C	-5.50322	-2.62897	1.50281
H	-4.97613	-2.96161	-0.55928
H	-6.15795	-1.69735	-0.32295
H	-6.29172	-3.38581	1.50155
H	-4.60593	-3.07471	1.93819
H	-5.81676	-1.81268	2.15774
C	-5.55050	1.06869	-0.02284
C	-5.93871	1.52678	-1.42833
H	-5.44835	1.94347	0.62742
H	-6.36572	0.47478	0.39969
H	-6.86365	2.10850	-1.40790
H	-5.15641	2.14750	-1.87113
H	-6.09054	0.66812	-2.08655

**Molecule 5**	X	Y	Z
N	0.89485	-1.81759	0.04182
N	1.82007	0.93126	0.01653
N	-1.85439	-0.89259	0.02280
N	-0.92913	1.85631	-0.01048
C	2.23498	-2.05619	0.04397
C	0.27552	-3.02912	0.05595
C	1.26026	-4.08909	0.06592
C	2.48489	-3.48122	0.05831
C	3.21720	-1.06836	0.04041
C	3.03170	0.31217	0.02713
C	4.09163	1.29696	0.02746
C	3.48363	2.52149	0.01607
C	2.05854	2.27143	0.00859
H	4.24483	-1.41256	0.05366
C	1.07072	3.25339	-0.01158
C	-0.30985	3.06786	-0.01968
H	1.41506	4.28100	-0.02656
C	-2.26918	2.09484	-0.02603
C	-1.29414	4.12768	-0.04363
C	-2.51896	3.51989	-0.04778
C	-3.25138	1.10703	-0.01426
C	-3.06593	-0.27341	0.00725
C	-2.09300	-2.23256	0.04410
C	-1.10505	-3.21466	0.05604
C	-3.51820	-2.48245	0.04400
C	-4.12599	-1.25797	0.02079
H	-4.27907	1.45132	-0.01865
H	-1.44924	-4.24237	0.06194
Mg	-0.01689	0.01893	0.01772
C	4.13267	3.86562	0.04459
C	4.21323	4.46590	1.44781
H	3.58755	4.55214	-0.61098
H	5.14075	3.78850	-0.37199
H	4.68543	5.45116	1.42367
H	3.21848	4.57659	1.88561

H	4.79763	3.82456	2.11186
C	5.55488	1.00241	0.00291
C	6.09003	0.73150	-1.40273
H	5.76830	0.14069	0.64316
H	6.09973	1.84413	0.43916
H	7.16172	0.51885	-1.37785
H	5.58491	-0.12363	-1.85802
H	5.92917	1.59483	-2.05269
C	3.82889	-4.13053	0.03044
C	4.42210	-4.22748	-1.37477
H	3.75415	-5.13359	0.45931
H	4.51874	-3.57751	0.67588
H	5.40735	-4.69968	-1.35006
H	3.77734	-4.81926	-2.02889
H	4.53098	-3.23789	-1.82449
C	0.96599	-5.55173	0.11714
C	0.70865	-6.06377	1.53392
H	1.80351	-6.10378	-0.31818
H	0.09821	-5.77552	-0.51125
H	0.49598	-7.13572	1.52865
H	1.57822	-5.89214	2.17268
H	-0.14217	-5.55125	1.98897
C	-4.16755	-3.82564	0.09774
C	-4.25456	-4.39655	1.51265
H	-5.17362	-3.75716	-0.32505
H	-3.61931	-4.52582	-0.54067
H	-4.72761	-5.38168	1.50699
H	-3.26183	-4.49899	1.95698
H	-4.84120	-3.74124	2.16095
C	-5.58884	-0.96393	-0.02534
C	-6.10981	-0.72848	-1.44269
H	-5.80804	-0.08615	0.59066
H	-6.13834	-1.79420	0.42677
H	-7.18149	-0.51452	-1.43392
H	-5.59931	0.11446	-1.91437
H	-5.94316	-1.60830	-2.06861
C	-3.86238	4.16873	-0.10190
C	-4.44140	4.23877	-1.51457
H	-3.79148	5.17968	0.30871
H	-4.55894	3.62835	0.54697
H	-5.42657	4.71170	-1.50897
H	-3.78983	4.81737	-2.17371
H	-4.54620	3.24052	-1.94583
C	-1.00010	5.59113	-0.02481
C	-0.74856	6.13555	1.38078
H	-1.83589	6.13260	-0.47626
H	-0.12956	5.80070	-0.65439
H	-0.53473	7.20693	1.35150
H	-1.62108	5.97978	2.01963

H	0.09976	5.63320	1.85165

**Molecule 6**	X	Y	Z
N	0.89003	-1.80800	0.04351
N	1.81011	0.93286	0.01736
N	-1.85102	-0.88800	0.02467
N	-0.93090	1.85269	-0.01172
C	2.22962	-2.04617	0.04557
C	0.27192	-3.01959	0.05722
C	1.25706	-4.07892	0.06671
C	2.48035	-3.47092	0.05945
C	3.21113	-1.06200	0.04211
C	3.02180	0.31493	0.02831
C	4.08116	1.30005	0.02793
C	3.47308	2.52325	0.01547
C	2.04828	2.27242	0.00819
H	4.23883	-1.40529	0.05549
C	1.06417	3.25374	-0.01290
C	-0.31281	3.06427	-0.02086
H	1.40756	4.28139	-0.02845
C	-2.27036	2.09090	-0.02609
C	-1.29756	4.12358	-0.04405
C	-2.52100	3.51568	-0.04741
C	-3.25192	1.10680	-0.01330
C	-3.06261	-0.27004	0.00880
C	-2.08925	-2.22742	0.04572
C	-1.10507	-3.20891	0.05737
C	-3.51417	-2.47813	0.04539
C	-4.12208	-1.25500	0.02218
H	-4.27967	1.45022	-0.01731
H	-1.44846	-4.23666	0.06292
C	4.12013	3.86832	0.04301
C	4.19944	4.46996	1.44572
H	3.57483	4.55359	-0.61371
H	5.12841	3.79159	-0.37310
H	4.67060	5.45568	1.42079
H	3.20447	4.58004	1.88314
H	4.78425	3.82983	2.11055
C	5.54418	1.00446	0.00410
C	6.07917	0.72790	-1.40049
H	5.75755	0.14545	0.64803
H	6.08909	1.84784	0.43699
H	7.15067	0.51449	-1.37457
H	5.57355	-0.12844	-1.85290
H	5.91914	1.58898	-2.05360
C	3.82528	-4.11819	0.03113
C	4.41878	-4.21321	-1.37408
H	3.75125	-5.12169	0.45904
H	4.51436	-3.56533	0.67751
H	5.40449	-4.68440	-1.34954

H	3.77472	-4.80512	-2.02874
H	4.52671	-3.22321	-1.82310
C	0.96177	-5.54138	0.11654
C	0.69927	-6.05412	1.53210
H	1.80081	-6.09318	-0.31608
H	0.09643	-5.76480	-0.51537
H	0.48590	-7.12591	1.52523
H	1.56682	-5.88356	2.17386
H	-0.15269	-5.54148	1.98486
C	-4.16145	-3.82227	0.09840
C	-4.24578	-4.39569	1.51253
H	-5.16835	-3.75389	-0.32244
H	-3.61438	-4.52075	-0.54280
H	-4.71870	-5.38086	1.50577
H	-3.25245	-4.49873	1.95533
H	-4.83165	-3.74146	2.16253
C	-5.58469	-0.95983	-0.02371
C	-6.10522	-0.71907	-1.44032
H	-5.80392	-0.08449	0.59576
H	-6.13441	-1.79165	0.42520
H	-7.17672	-0.50431	-1.43079
H	-5.59416	0.12504	-1.90927
H	-5.93922	-1.59688	-2.06919
C	-3.86541	4.16253	-0.09994
C	-4.44613	4.23267	-1.51186
H	-3.79469	5.17348	0.31071
H	-4.56054	3.62166	0.55005
H	-5.43186	4.70439	-1.50462
H	-3.79603	4.81278	-2.17110
H	-4.55025	3.23482	-1.94420
C	-1.00190	5.58668	-0.02542
C	-0.74253	6.13001	1.37923
H	-1.83969	6.12903	-0.47206
H	-0.13477	5.79604	-0.65977
H	-0.52810	7.20121	1.34956
H	-1.61182	5.97440	2.02248
H	0.10777	5.62670	1.84544
Zn	-0.02006	0.02168	0.01848

**Molecule 7**	X	Y	Z
N	0.00275	2.09630	0.00216
N	-2.02429	0.00434	-0.00022
N	2.02470	-0.00294	0.00089
N	-0.00239	-2.09490	-0.00285
C	-1.11800	2.87447	-0.00210
C	1.12631	2.87037	0.00277
H	0.00012	1.08544	0.01081
C	0.68782	4.22751	-0.00631
C	-0.67470	4.23003	-0.00076
C	-2.44060	2.42965	0.00355

C	-2.84332	1.08598	0.00564
C	-4.23580	0.68147	-0.00193
C	-4.23757	-0.66689	-0.00129
C	-2.84618	-1.07511	-0.00714
C	-2.44712	-2.41986	-0.00499
C	-1.12600	-2.86891	0.00113
C	1.11833	-2.87311	-0.00240
H	0.00031	-1.08403	-0.01021
C	-0.68755	-4.22611	0.00022
C	0.67494	-4.22863	0.00658
C	2.44095	-2.42832	0.00404
C	2.84373	-1.08459	0.00681
C	2.84656	1.07658	-0.00482
C	2.44754	2.42138	-0.00252
C	4.23793	0.66833	0.00191
C	4.23616	-0.68004	0.00042
H	5.09063	1.32932	0.00728
H	5.08709	-1.34331	-0.00493
H	1.32769	-5.08658	0.01639
H	-1.34356	-5.08163	-0.00308
H	-5.09009	-1.32815	0.00267
H	-1.32758	5.08792	0.00223
H	-5.08665	1.34485	-0.00720
H	1.34379	5.08300	-0.01572
C	-3.49554	3.48088	0.00766
C	-4.23780	3.73672	1.15906
C	-3.75739	4.22897	-1.13851
C	-5.21941	4.71724	1.16428
H	-4.03915	3.16257	2.05744
C	-4.74054	5.20821	-1.13485
H	-3.18859	4.03456	-2.04132
C	-5.47417	5.45557	0.01693
H	-5.78545	4.90580	2.06967
H	-4.93525	5.77733	-2.03700
H	-6.24197	6.22092	0.02040
C	3.50542	3.46963	-0.00784
C	3.76771	4.21971	1.13695
C	4.24991	3.72086	-1.15883
C	4.75352	5.19626	1.13237
H	3.19714	4.02893	2.03940
C	5.23406	4.69881	-1.16503
H	4.05089	3.14522	-2.05618
C	5.48931	5.43904	-0.01900
H	4.94858	5.76691	2.03348
H	5.80173	4.88385	-2.07011
H	6.25915	6.20233	-0.02321
C	3.49564	-3.47969	0.01005
C	4.24000	-3.73181	1.16095
C	3.75502	-4.23198	-1.13397

C	5.22119	-4.71273	1.16777
H	4.04316	-3.15455	2.05773
C	4.73791	-5.21145	-1.12878
H	3.18456	-4.04055	-2.03636
C	5.47360	-5.45507	0.02248
H	5.78876	-4.89844	2.07279
H	4.93077	-5.78377	-2.02931
H	6.24113	-6.22068	0.02718
C	-3.50528	-3.46800	-0.00968
C	-3.77161	-4.21346	1.13715
C	-4.24602	-3.72334	-1.16213
C	-4.75762	-5.18981	1.13304
H	-3.20394	-4.01936	2.04073
C	-5.23058	-4.70091	-1.16776
H	-4.04392	-3.15111	-2.06098
C	-5.48975	-5.43673	-0.01979
H	-4.95580	-5.75702	2.03564
H	-5.79547	-4.88913	-2.07394
H	-6.25983	-6.19978	-0.02360

**Molecule 8**	X	Y	Z
N	0.05654	-2.04230	-0.00736
N	2.04709	0.06259	-0.00660
N	-2.04850	-0.05173	-0.00673
N	-0.05786	2.05331	-0.00589
C	1.17622	-2.82202	-0.01045
C	-1.01707	-2.88387	-0.00214
C	-0.55939	-4.24719	-0.00686
C	0.79603	-4.20905	-0.00315
C	2.50013	-2.35866	-0.00556
C	2.88872	-1.01093	-0.00148
C	4.25202	-0.55318	-0.00533
C	4.21380	0.80223	-0.00102
C	2.82673	1.18235	-0.00881
C	2.36340	2.50626	-0.00373
C	1.01569	2.89492	-0.00021
C	-1.17758	2.83301	-0.00845
C	0.55800	4.25820	-0.00406
C	-0.79745	4.22002	-0.00033
C	-2.50150	2.36958	-0.00391
C	-2.89008	1.02190	-0.00088
C	-2.82823	-1.17142	-0.00971
C	-2.36479	-2.49533	-0.00541
C	-4.21524	-0.79122	-0.00168
C	-4.25338	0.56421	-0.00504
H	-5.05182	-1.47230	0.00569
H	-5.12681	1.19735	-0.00926
H	-1.47849	5.05663	0.00729
H	1.19107	5.13169	-0.00835
H	5.05036	1.48333	0.00681

H	1.47714	-5.04561	0.00395
H	5.12546	-1.18631	-0.01001
H	-1.19256	-5.12060	-0.01166
C	3.58235	-3.38178	-0.00232
C	4.33903	-3.61232	1.14524
C	3.85929	-4.12813	-1.14638
C	5.34630	-4.56662	1.15004
H	4.13012	-3.04008	2.04259
C	4.86803	-5.08092	-1.14378
H	3.27985	-3.95430	-2.04659
C	5.61439	-5.30362	0.00493
H	5.92187	-4.73583	2.05329
H	5.07270	-5.64911	-2.04436
H	6.40207	-6.04851	0.00767
C	-3.38793	-3.57753	-0.00187
C	-3.61786	-4.33441	1.14567
C	-4.13491	-3.85424	-1.14558
C	-4.57220	-5.34165	1.15082
H	-3.04510	-4.12569	2.04274
C	-5.08773	-4.86295	-1.14263
H	-3.96156	-3.27464	-2.04578
C	-5.30982	-5.60951	0.00608
H	-4.74092	-5.91737	2.05407
H	-5.65642	-5.06746	-2.04293
H	-6.05474	-6.39716	0.00908
C	-3.58371	3.39274	0.00013
C	-4.33994	3.62281	1.14806
C	-3.86102	4.13953	-1.14354
C	-5.34719	4.57713	1.15361
H	-4.13073	3.05017	2.04508
C	-4.86974	5.09234	-1.14018
H	-3.28190	3.96606	-2.04403
C	-5.61567	5.31459	0.00890
H	-5.92244	4.74599	2.05713
H	-5.07473	5.66091	-2.04045
H	-6.40334	6.05950	0.01222
C	3.38655	3.58846	0.00034
C	3.61651	4.34475	1.14827
C	4.13342	3.86579	-1.14328
C	4.57078	5.35204	1.15387
H	3.04382	4.13553	2.04527
C	5.08618	4.87456	-1.13988
H	3.96002	3.28664	-2.04375
C	5.30830	5.62053	0.00920
H	4.73955	5.92731	2.05739
H	5.65479	5.07956	-2.04011
H	6.05317	6.40824	0.01254
Mg	-0.00116	0.00542	-0.00763

Molecule 9	X	Y	Z
N	0.06525	-2.02888	-0.00203
N	2.03487	0.08348	-0.00122
N	-2.04762	-0.05975	-0.00140
N	-0.07810	2.05250	-0.00096
C	1.18992	-2.80056	-0.00524
C	-1.00178	-2.87802	0.00318
C	-0.53442	-4.23782	-0.00144
C	0.81965	-4.19009	0.00199
C	2.50873	-2.33189	-0.00040
C	2.88395	-0.98351	0.00382
C	4.24380	-0.51613	0.00039
C	4.19603	0.83791	0.00469
C	2.80645	1.20814	-0.00325
C	2.33742	2.52680	0.00156
C	0.98892	2.90163	0.00468
C	-1.20272	2.82424	-0.00374
C	0.52163	4.26144	0.00077
C	-0.83247	4.21373	0.00417
C	-2.52157	2.35560	0.00075
C	-2.89672	1.00723	0.00413
C	-2.81921	-1.18441	-0.00406
C	-2.35023	-2.50313	0.00018
C	-4.20878	-0.81420	0.00397
C	-4.25655	0.53987	0.00036
H	-5.03935	-1.50249	0.01154
H	-5.13340	1.16816	-0.00381
H	-1.52066	5.04439	0.01150
H	1.14989	5.13831	-0.00336
H	5.02660	1.52620	0.01265
H	1.50793	-5.02067	0.00892
H	5.12062	-1.14448	-0.00406
H	-1.16274	-5.11465	-0.00601
C	3.59799	-3.34747	0.00264
C	4.35156	-3.57744	1.15220
C	3.88388	-4.08688	-1.14357
C	5.36502	-4.52516	1.15685
H	4.13551	-3.01013	2.05099
C	4.89876	-5.03312	-1.14095
H	3.30650	-3.91318	-2.04513
C	5.64214	-5.25564	0.00972
H	5.93834	-4.69430	2.06153
H	5.11053	-5.59631	-2.04301
H	6.43462	-5.99543	0.01241
C	-3.36565	-3.59255	0.00393
C	-3.59454	-4.34614	1.15369
C	-4.10598	-3.87851	-1.14167
C	-4.54213	-5.35973	1.15914

H	-3.02648	-4.13004	2.05199
C	-5.05207	-4.89353	-1.13825
H	-3.93310	-3.30110	-2.04336
C	-5.27353	-5.63694	0.01262
H	-4.71044	-5.93307	2.06396
H	-5.61599	-5.10538	-2.03983
H	-6.01320	-6.42952	0.01591
C	-3.61082	3.37119	0.00438
C	-4.36409	3.60078	1.15419
C	-3.89694	4.11096	-1.14155
C	-5.37756	4.54851	1.15937
H	-4.14786	3.03315	2.05273
C	-4.91181	5.05721	-1.13838
H	-3.31974	3.93756	-2.04328
C	-5.65492	5.27936	0.01254
H	-5.95068	4.71736	2.06423
H	-5.12378	5.62070	-2.04021
H	-6.44740	6.01915	0.01565
C	3.35280	3.61626	0.00582
C	3.58166	4.36938	1.15589
C	4.09313	3.90269	-1.13965
C	4.52923	5.38299	1.16176
H	3.01359	4.15291	2.05410
C	5.03921	4.91773	-1.13580
H	3.92028	3.32563	-2.04157
C	5.26063	5.66067	0.01536
H	4.69752	5.95597	2.06681
H	5.60314	5.12995	-2.03729
H	6.00030	6.45326	0.01898
Zn	-0.00653	0.01155	-0.00142

**Molecule 10**	X	Y	Z
N	0.03114	-2.11503	-0.03352
N	2.00699	0.01591	0.10419
N	-2.02790	-0.05852	-0.10426
N	-0.05234	2.07108	0.03215
C	1.16477	-2.87063	0.03576
C	-1.07340	-2.91277	-0.09658
H	0.01268	-1.10448	-0.04679
C	-0.60868	-4.26138	-0.06708
C	0.75107	-4.23574	0.01271
C	2.47543	-2.40138	0.12392
C	2.84657	-1.04871	0.15661
C	4.22745	-0.61646	0.25731
C	4.20126	0.73223	0.26666
C	2.80453	1.11168	0.17145
C	2.38136	2.44937	0.15738
C	1.05314	2.86886	0.08253
C	-1.18656	2.82637	-0.03896
H	-0.03505	1.06053	0.05241

C	0.58907	4.21711	0.04080
C	-0.77130	4.19157	-0.03410
C	-2.49869	2.35712	-0.11084
C	-2.86950	1.00465	-0.13953
C	-2.82528	-1.15482	-0.16995
C	-2.40176	-2.49233	-0.16790
C	-4.22365	-0.77588	-0.24694
C	-4.25123	0.57210	-0.22329
H	-5.05636	-1.45948	-0.31504
H	-5.11105	1.22320	-0.26342
H	-1.44433	5.03257	-0.09044
H	1.23230	5.08257	0.05943
H	5.03371	1.41550	0.34101
H	1.42509	-5.07652	0.05639
H	5.08582	-1.26785	0.32120
H	-1.25103	-5.12697	-0.09987
C	3.55285	-3.43075	0.18643
C	3.97841	-3.92872	1.41301
C	4.15109	-3.90484	-0.99378
C	4.98160	-4.88171	1.50333
H	3.50729	-3.55047	2.31425
C	5.16332	-4.86584	-0.89593
C	5.57191	-5.34583	0.33487
H	5.29802	-5.25383	2.47018
H	5.62758	-5.23352	-1.80568
H	6.36048	-6.08903	0.38030
C	-3.43812	-3.56234	-0.23896
C	-4.08120	-4.00975	0.92828
C	-3.77619	-4.12978	-1.46283
C	-5.04881	-5.01465	0.81953
C	-4.73613	-5.12525	-1.56369
C	-5.37112	-5.56264	-0.40843
H	-5.54617	-5.36361	1.71907
H	-4.98522	-5.55037	-2.52840
H	-6.12690	-6.33865	-0.46204
C	-3.57470	3.38835	-0.16416
C	-4.10390	3.92661	1.02151
C	-4.06239	3.83043	-1.38926
C	-5.10800	4.89684	0.93037
C	-5.05875	4.79097	-1.47275
H	-3.64216	3.40538	-2.29480
C	-5.57739	5.32206	-0.29880
H	-5.52022	5.31253	1.84448
H	-5.42256	5.12008	-2.43854
H	-6.35584	6.07616	-0.33853
C	3.41822	3.51947	0.21945
C	3.78991	4.06676	1.44258
C	4.02985	3.98548	-0.95735
C	4.75327	5.06000	1.53340

H	3.30953	3.69405	2.34126
C	5.00099	4.98805	-0.85901
C	5.35701	5.51579	0.36857
H	5.02889	5.46933	2.49772
H	5.47439	5.35094	-1.76593
H	6.11466	6.29047	0.41432
N	3.71502	-3.47577	-2.23165
H	3.24240	-2.58566	-2.24921
H	4.36937	-3.59024	-2.98860
N	-3.68897	3.46957	2.25707
H	-2.78338	3.02793	2.28357
H	-3.84283	4.09851	3.02852
N	3.64304	3.50923	-2.19407
H	3.20356	2.60221	-2.20101
H	4.31118	3.63077	-2.93771
N	-3.72826	-3.51459	2.16806
H	-3.29421	-2.60481	2.17281
H	-4.41886	-3.62394	2.89305
H	-3.27126	-3.77155	-2.35389

**Molecule 11**	X	Y	Z
N	0.33097	-2.15104	-0.03399
N	-1.95906	-0.31891	0.05100
N	-0.32602	2.13842	0.00414
C	1.55694	-2.74153	-0.21026
C	-0.67084	-3.06768	-0.10476
H	0.14931	-1.18561	0.19510
C	-0.05311	-4.32159	-0.38469
C	1.29597	-4.12182	-0.44902
C	2.79257	-2.09111	-0.14378
C	2.90950	-0.68739	-0.07188
C	4.05758	0.03129	0.45696
C	2.58155	1.53623	-0.00979
C	2.04284	2.83481	-0.05620
C	0.66425	3.07142	-0.16196
C	-1.55733	2.69841	-0.13316
H	-0.20509	1.17525	0.27677
C	0.00393	4.29670	-0.45384
C	-1.34495	4.07160	-0.44091
C	-2.77807	2.02174	0.01318
C	-2.92828	0.63391	0.16440
C	-2.59754	-1.51282	0.19273
C	-2.03467	-2.79566	0.06592
C	-4.00814	-1.30914	0.46693
C	-4.21359	0.02208	0.44576
H	-4.73155	-2.08476	0.66395
H	-5.13777	0.54993	0.62229
H	-2.12616	4.78619	-0.64442
H	0.50568	5.22352	-0.68147
H	2.05072	-4.85853	-0.67309

H	4.96191	-0.42473	0.84288
H	-0.58214	-5.24870	-0.53623
C	4.02059	-2.90953	-0.10777
C	4.15545	-3.98595	0.77364
C	5.09110	-2.60345	-0.95277
C	5.32026	-4.73661	0.80200
H	3.34843	-4.21738	1.45926
C	6.25344	-3.35843	-0.92773
H	4.99755	-1.77711	-1.64847
C	6.37187	-4.42849	-0.05105
H	5.41029	-5.56093	1.50055
H	7.06812	-3.11154	-1.59918
H	7.28193	-5.01726	-0.02919
C	-2.94038	-3.97351	0.09499
C	-2.82170	-4.93662	1.09763
C	-3.92040	-4.14692	-0.88311
C	-3.66512	-6.03774	1.12721
H	-2.06852	-4.81185	1.86799
C	-4.76119	-5.24998	-0.85716
H	-4.01614	-3.41232	-1.67515
C	-4.63722	-6.19855	0.14910
H	-3.56420	-6.77069	1.91993
H	-5.51311	-5.37023	-1.62912
H	-5.29528	-7.05992	0.17043
C	-3.99925	2.86844	-0.01263
C	-4.20840	3.84485	0.96199
C	-4.95317	2.71522	-1.01938
C	-5.34363	4.64186	0.93548
H	-3.47841	3.97029	1.75426
C	-6.08542	3.51624	-1.05044
H	-4.79775	1.96718	-1.78898
C	-6.28519	4.48096	-0.07203
H	-5.49410	5.38888	1.70687
H	-6.81247	3.38775	-1.84459
H	-7.17140	5.10513	-0.09479
C	2.94609	3.99715	0.03652
C	2.69874	5.03825	0.93407
C	4.08010	4.07154	-0.77604
C	3.55790	6.12383	1.01431
H	1.83868	4.98205	1.59154
C	4.93193	5.16120	-0.70328
H	4.28213	3.27114	-1.47754
C	4.67502	6.19096	0.19329
H	3.35614	6.91648	1.72611
H	5.80167	5.20719	-1.34906
H	5.34563	7.04076	0.25409
C	1.98291	0.31840	-0.36526
H	1.06469	0.20143	-0.91894
N	3.87370	1.31552	0.49574

Molecule 12	X	Y	Z
N	0.00088	-2.06030	0.00014
N	2.13901	-0.00095	0.00007
N	-2.13901	-0.00278	0.00021
N	-0.00088	2.05589	0.00014
C	1.09425	-2.85990	0.00011
C	-1.09179	-2.86085	0.00017
C	-0.69823	-4.26367	0.00015
C	0.70190	-4.26306	0.00012
C	2.41910	-2.43226	0.00008
C	2.89420	-1.13576	0.00006
C	2.89384	1.13423	0.00006
C	4.26821	0.70347	0.00004
C	4.26848	-0.70428	0.00005
C	2.41712	2.43012	0.00007
C	1.09186	2.85646	0.00011
C	-1.09433	2.85550	0.00017
C	0.69821	4.25923	0.00011
C	-0.70189	4.25862	0.00015
C	-2.41923	2.42803	0.00021
C	-2.89484	1.13174	0.00022
C	-2.89321	-1.13825	0.00021
C	-2.41701	-2.43434	0.00020
C	-4.26883	0.69977	0.00023
C	-4.26786	-0.70798	0.00023
H	-3.17531	-3.20889	0.00021
H	3.17806	-3.20617	0.00007
H	3.17485	3.20527	0.00005
H	-3.17760	3.20255	0.00023
C	-1.41163	-5.45776	0.00017
C	-0.69687	-6.64521	0.00015
C	0.70262	-6.64461	0.00012
C	1.41634	-5.45653	0.00010
C	-5.46709	-1.42121	0.00023
C	-6.64866	-0.70796	0.00024
C	-6.64962	0.69648	0.00025
C	-5.46904	1.41138	0.00024
C	-1.41631	5.45213	0.00016
C	-0.70265	6.64018	0.00014
C	0.69689	6.64079	0.00010
C	1.41159	5.45336	0.00009
C	5.46778	1.41615	0.00003
C	6.64899	0.70231	0.00002
C	6.64927	-0.70214	0.00003
C	5.46835	-1.41644	0.00004
H	-2.50100	5.46310	0.00019
H	-1.23371	7.58554	0.00015
H	1.22713	7.58661	0.00009

H	2.49626	5.46528	0.00006
H	-7.59614	1.22493	0.00025
H	-5.47937	2.49552	0.00024
H	-5.47587	-2.50537	0.00022
H	-7.59449	-1.23763	0.00024
H	-2.49629	-5.47010	0.00020
H	-1.22739	-7.59087	0.00016
H	2.50101	-5.46792	0.00007
H	1.23396	-7.58980	0.00010
H	7.59558	-1.23097	0.00002
H	5.47811	-2.50059	0.00004
H	5.47712	2.50030	0.00003
H	7.59504	1.23159	0.00001
H	-1.12723	-0.00133	0.00023
H	1.12723	-0.00038	0.00005

**Molecule 13**	X	Y	Z
N	0.00046	2.07537	0.04184
N	-2.06943	0.00591	0.04528
N	2.06932	0.00541	0.04523
N	-0.00017	-2.06440	0.04646
C	-1.10589	2.86874	0.03901
C	1.10690	2.86861	0.03892
C	0.70162	4.25929	0.03408
C	-0.70042	4.25939	0.03414
C	-2.41837	2.42477	0.04100
C	-2.86265	1.11247	0.04429
C	-2.86290	-1.10052	0.04698
C	-4.25345	-0.69488	0.04790
C	-4.25329	0.70714	0.04622
C	-2.41908	-2.41301	0.04713
C	-1.10682	-2.85754	0.04649
C	1.10621	-2.85791	0.04643
C	-0.70156	-4.24815	0.04653
C	0.70050	-4.24838	0.04650
C	2.41852	-2.41367	0.04707
C	2.86258	-1.10120	0.04688
C	2.86296	1.11170	0.04415
C	2.41921	2.42424	0.04089
C	4.25321	-0.69601	0.04770
C	4.25345	0.70603	0.04599
H	3.18601	3.19049	0.03923
H	-3.18478	3.19143	0.03941
H	-3.18580	-3.17935	0.04774
H	3.18522	-3.18003	0.04764
C	1.41518	5.45628	0.02949
C	0.70184	6.64109	0.02513
C	-0.70039	6.64119	0.02521
C	-1.41385	5.45645	0.02964
C	5.45064	1.41933	0.04626

C	6.63527	0.70576	0.04819
C	6.63504	-0.69650	0.04990
C	5.45017	-1.40970	0.04970
C	1.41376	-5.44558	0.04642
C	0.70015	-6.63020	0.04639
C	-0.70210	-6.62997	0.04643
C	-1.41527	-5.44509	0.04650
C	-5.45059	-1.40824	0.05009
C	-6.63526	-0.69469	0.05050
C	-6.63510	0.70755	0.04882
C	-5.45024	1.42080	0.04672
H	2.49828	-5.45679	0.04630
H	1.23015	-7.57609	0.04628
H	-1.23236	-7.57571	0.04637
H	-2.49980	-5.45579	0.04646
H	7.58076	-1.22677	0.05145
H	5.46100	-2.49422	0.05108
H	5.46178	2.50385	0.04500
H	7.58117	1.23572	0.04843
H	2.49970	5.46724	0.02920
H	1.23205	7.58684	0.02144
H	-2.49838	5.46745	0.02948
H	-1.23044	7.58703	0.02159
H	-7.58083	1.23780	0.04924
H	-5.46108	2.50532	0.04551
H	-5.46172	2.49276	0.05144
H	-7.58113	-1.22471	0.05219
Zn	-0.00042	0.00341	0.04638

Table S2: Calculated Cartesian coordinates of ground state optimized geometries (in Å) for molecules (**1**, **2**, and **7**), Determined at the B3LYP/def2-TZVP level in solvent with CPCM solvation model.

**Molecule 1**	X	Y	Z
N	0.23647922913400	3.06641305908687	-0.02704759936445
N	2.39778522993855	1.08532657674614	0.01823727009398
N	-1.65680502086227	0.83016258303982	0.02245277333273
N	0.50449472152383	-1.15088453449379	0.06778242451500
C	1.31218839160172	3.91214896779309	-0.04089197522445
C	-0.93780284959624	3.76945431933073	-0.03879491641587
H	0.30033379826172	2.05748619378901	-0.01392744988981
C	-0.58169939213223	5.15590820818532	-0.06230785449492
C	0.78375368447735	5.24252036699239	-0.06362942437488
C	2.64716326027721	3.52467432724855	-0.03205576707366
C	3.14622708773992	2.22047267898059	-0.00485368966499
C	4.56714582239724	1.90202026100531	0.00256665368769
C	4.65347307965607	0.55187101194856	0.03063814492674
C	3.28475109641759	0.05516250885899	0.04018787305586
H	3.37332050514548	4.32779718606885	-0.04675606161963
C	2.95446859748487	-1.30173813559298	0.06830877869495
C	1.67879973371279	-1.85387140226958	0.07951046289264
H	3.77597903895940	-2.00702219116400	0.08177315805724
C	-0.57124058152221	-1.99657920371303	0.08157106068247
H	0.44062484871772	-0.14195859367963	0.05460815950849
C	1.32268240356775	-3.24033310493021	0.10314028362993
C	-0.04277972119875	-3.32695789601118	0.10413177908430
C	-1.90621428770706	-1.60914464244401	0.07271293307363
C	-2.40527672743343	-0.30491821190122	0.04553935610876
C	-2.54374873775116	1.86039639488588	0.00049654282779
C	-2.21346520911732	3.21727567056706	-0.02759043825241
C	-3.91246325238696	1.36372197778285	0.01027019502265
C	-3.82620640405405	0.01355837602381	0.03786476770243
H	-2.63225542714720	-2.41237122942984	0.08730162375810
H	-3.03508254827329	3.92244094169757	-0.04097051775012
H	-4.79972278290091	1.97890802049992	-0.00293702489568
H	-4.62778237411614	-0.70971863441434	0.05209976877336
H	-0.64641598965638	-4.22060823441920	0.11824238178701
H	2.03451274591924	-4.05047179621847	0.11620649102427
H	5.54070167551145	-0.06335203827979	0.04402071155970
H	1.38783920405586	6.13586651009127	-0.07787444407853
H	5.36875341168099	2.62526171614613	-0.01186056939120
H	-1.29392626032515	5.96570199219256	-0.07525586130911

**Molecule 2**	X	Y	Z
N	0.94005807881948	3.29611821279538	0.36872628676236
N	2.81384698086768	1.06901400042217	0.32431091261673
N	-1.28406736228273	1.43027856249912	0.16426188511394
N	0.58970124606833	-0.79691260746334	0.11985171537424
Mg	0.76492775441794	1.24931082785349	0.24426028541787

C	2.10576512310744	4.00648767474733	0.46336252550236
C	-0.08712275414050	4.20008995852664	0.37552360118172
C	0.44973178715738	5.53604266421758	0.47831530937473
C	1.80434390355723	5.41648133601687	0.53279752914005
C	3.38571051521836	3.44869715115967	0.48966648161263
C	3.71543866750739	2.09320794719620	0.42603130625345
C	3.52770062871312	-0.09785642651411	0.29144362462173
C	4.93776506423375	0.19971626052975	0.37529794428711
C	5.05363396417284	1.55318557715396	0.45855909979995
C	2.97258530228105	-1.37542239969776	0.19302011036857
C	1.61689777755118	-1.70090369506208	0.11303448526458
C	-0.57601394910448	-1.50730854194825	0.02522610784023
C	1.08004723231333	-3.03682714339006	0.01038823671517
C	-0.27458269661515	-2.91725253663323	-0.04433839597551
C	-1.85594157947223	-0.94946329948912	-0.00109229874520
C	-2.18562759183058	0.40603146211808	0.06252377996200
C	-1.99795354325940	2.59709601768348	0.19708589605586
C	-1.44281380816650	3.87465748250600	0.29551765098259
C	-3.52383980058716	0.94603326857470	0.02990307610099
C	-3.40801031248504	2.29950105718395	0.11321347905973
H	2.53795840185454	6.20438138371561	0.61235388261182
H	-0.13898973382286	6.44048476155403	0.50502117985247
H	-4.42724128714780	0.36015493524692	-0.04718360882542
H	-4.19847306978108	3.03465289553048	0.11721013241296
H	5.72821579866152	-0.53544971011073	0.37128456920135
H	5.95705192756305	2.13904372980867	0.53559266675416
H	1.66868623790155	-3.94132428745757	-0.01623299722660
H	-1.00812103740495	-3.70521272963998	-0.12398599860809
H	-2.13983890407293	4.70375456688107	0.31129561723188
H	4.21311303085452	4.14343021331039	0.56879107144168
H	3.66965699869689	-2.20448057885761	0.17725916231519
H	-2.68338899134517	-1.64413799096771	-0.08025631184927

**Molecule 7**	X	Y	Z
N	0.00343693083033	2.10348298250342	0.00419499698856
N	-2.03794424834391	0.00359284122662	-0.00023229360666
N	2.03795386042103	-0.00218844901225	0.00051460504621
N	-0.00343792208681	-2.10207838358805	-0.00437822165338
C	-1.12475303029239	2.88437875408689	0.00228155119676
C	1.13389122251410	2.88116072919032	0.00287621208479
H	0.00239834747414	1.09293670413629	0.00575580173329
C	0.68920920873238	4.24121884335312	0.00072101356485
C	-0.67612079365989	4.24316373429967	0.00033805894963
C	-2.45020456344729	2.43821655432091	0.00048884678056
C	-2.85737481143403	1.09251086762109	-0.00063783626355
C	-4.25420936475025	0.68246555795309	-0.00184673096983
C	-4.25640209666214	-0.66807460272951	-0.00220966928888
C	-2.86092472290086	-1.08264076206832	-0.00106299813467
C	-2.45798883287375	-2.42962185378845	-0.00173387373959
C	-1.13388322588505	-2.87976535678908	-0.00263048011935

C	1.12476193865183	-2.88296381850344	-0.00209287030657
H	-0.00241117889986	-1.09153278262038	-0.00648185298199
C	-0.68918793844880	-4.23981870272181	0.00047094758792
C	0.67614178343159	-4.24174882966827	0.00073617737886
C	2.45021468642194	-2.43679771976070	-0.00163359695634
C	2.85738787574024	-1.09110198718470	-0.00059263896469
C	2.86093118346314	1.08404408543597	0.00163448240103
C	2.45799034972298	2.43101357813729	0.00281499015778
C	4.25641505466273	0.66948564513494	0.00121473631680
C	4.25422518301335	-0.68105361848551	-0.00035599020284
H	5.11022918096566	1.32688953481652	0.00186840015025
H	5.10592557988632	-1.34119289151730	-0.00124450377440
H	1.32524551634812	-5.10076083155263	0.00320970273390
H	-1.34071635831092	-5.09699681601476	0.00306834381391
H	-5.11023606147926	-1.32545149682441	-0.00358820545621
H	-1.32521557989558	5.10218394129765	-0.00181098008908
H	-5.10591127530479	1.34260261262651	-0.00217647729357
H	1.34073690008654	5.09839988901765	-0.00072312528841
C	-3.51211738694274	3.49732943539076	-0.00104831798659
C	-4.01046036964883	4.00699502434730	1.19986348505113
C	-4.02018491859546	3.99356510647619	-1.20352769013974
C	-4.99599883389772	4.98930828772326	1.19917099162046
H	-3.62436647377752	3.62954748445437	2.13907105524179
C	-5.00556377681680	4.97601259988734	-1.20586434214638
H	-3.64148047891013	3.60575699447563	-2.14151924255899
C	-5.49635227938037	5.47656236701480	-0.00410346389767
H	-5.37229096407608	5.37277115795497	2.13973060582425
H	-5.38925453955790	5.34915781731939	-2.14758524880564
H	-6.26360685067602	6.24095009726459	-0.00527424774933
C	3.52311426569745	3.48689235825608	0.00313340133080
C	4.02324661789228	3.99241234137138	1.20506666385195
C	4.03244402047046	3.98421679288816	-1.19834285186222
C	5.01168867664750	4.97178994170366	1.20634177301024
H	3.63613437111348	3.61409716430649	2.14350449128596
C	5.02076472333803	4.96372236947186	-1.19871151414244
H	3.65250941136935	3.59955928898180	-2.13713533132489
C	5.51326385075143	5.46019017908904	0.00403967713187
H	5.38931077052886	5.35204679677080	2.14767048300271
H	5.40542704939708	5.33773166478717	-2.13969221175044
H	6.28281600266028	6.22226494620010	0.00438260771859
C	3.51212163789880	-3.49593384471874	-0.00124920949625
C	4.02159382630644	-3.99225677655127	1.20058449285289
C	4.00904994961947	-4.00552434389230	-1.20278363997432
C	5.00694505217995	-4.97474539181387	1.20168421463389
H	3.64408358394953	-3.60448994286139	2.13907503628831
C	4.99458717436281	-4.98783049400130	-1.20333184392421
H	3.62179714818113	-3.62803634517327	-2.14149765183958
C	5.49632938701316	-5.47520612539651	-0.00068473380222
H	5.39171447065852	-5.34797664649868	2.14293016183922

H	5.36977561863116	-5.37120843443410	-2.14436745237592
H	6.26357137733028	-6.23960649722216	-0.00046714490176
C	-3.52311248080056	-3.48547793392737	-0.00073817036202
C	-4.03777107203054	-3.97554396011092	1.20148249855671
C	-4.01791947895197	-3.99825066443383	-1.20178857826146
C	-5.02601394421355	-4.95510615552180	1.20340360214575
H	-3.66184199475912	-3.58528688773927	2.13957245686116
C	-5.00645121111901	-4.97755500111073	-1.20151061240931
H	-3.62676930313510	-3.62552315407862	-2.14078973115064
C	-5.51326872195496	-5.45874952123410	0.00149630761241
H	-5.41475451659530	-5.32353461717367	2.14491084056032
H	-5.38001525572625	-5.36340648918023	-2.14218045173147
H	-6.28282693212233	-6.22081894138939	0.00234231437803

Table S3: Calculated Cartesian coordinates of ground state optimized geometries (in Å) for molecules (**1**, **2**, and **7**), Determined at the M06-2X/def2-TZVP level in solvent with CPCM solvation model.

**Molecule 1**	X	Y	Z
N	0.23498691375317	3.05158083994968	-0.03159253650615
N	2.37702077167939	1.08079584426035	0.01879895725830
N	-1.63604330887576	0.83472776216851	0.02187625279226
N	0.50599635625822	-1.13606147560538	0.07229450504855
C	1.30486979666990	3.89550490846466	-0.04194057753712
C	-0.93226790707657	3.75604772140008	-0.04178475973769
H	0.29644139846282	2.04133112646186	-0.02379296184758
C	-0.57847391164146	5.14231984714016	-0.06097450746511
C	0.78210858335933	5.22659620820961	-0.06089287114353
C	2.63695362819003	3.51418805189021	-0.03275904926615
C	3.12489987545004	2.21069872191913	-0.00581303620548
C	4.54648706405248	1.89775669950725	0.00130132553017
C	4.63376495932901	0.55288238170692	0.03048798249071
C	3.26532802808035	0.05879542137550	0.04168363096679
H	3.36371107464211	4.31646123593802	-0.04660989175024
C	2.94781287967194	-1.29667679681272	0.07193599360078
C	1.67326066509401	-1.84054577005022	0.08248740079591
H	3.77034335927290	-2.00043253349312	0.08526209744435
C	-0.56389857300547	-1.97999492639805	0.08265055585665
H	0.44454396217912	-0.12581411980742	0.06444547379779
C	1.31944823486944	-3.22681564434175	0.10153460624346
C	-0.04112879712307	-3.31108120146318	0.10181196530157
C	-1.89596068613844	-1.59867542721290	0.07344533869669
C	-2.38390689103820	-0.29516745743626	0.04644233436295
C	-2.52436717279766	1.85673410013858	-0.00100366275448
C	-2.20684347883445	3.21218438016519	-0.03126736164242
C	-3.89280675897274	1.36263306852787	0.00981838452702
C	-3.80549056284690	0.01777494969106	0.03964639256765
H	-2.62272178143629	-2.40094419798963	0.08739102031957
H	-3.02936733527468	3.91594549643817	-0.04470447584497
H	-4.77665052768502	1.98246772697227	-0.00305075217864
H	-4.60108229963290	-0.71144001036103	0.05637463931709
H	-0.65130704945847	-4.20043172041969	0.11141522052196
H	2.03455148619637	-4.03387955603712	0.11207194056776
H	5.51759084767275	-0.06698669301724	0.04308607900558
H	1.39236561687263	6.11589416805827	-0.07033059254624
H	5.34209784579799	2.62695281429772	-0.01521107310164
H	-1.29367630571591	5.94929405576466	-0.07162398748613
**Molecule 2**	X	Y	Z
N	0.93752500511153	3.28190750077280	0.37051134348673
N	2.80043230911925	1.07075599463063	0.32287119787333
N	-1.27064304926326	1.42860853038071	0.16582421489029
N	0.59221313540206	-0.78274824512567	0.11821835008896
Mg	0.76494446638151	1.24917821671809	0.24436617924155

C	2.09776705832822	3.99032221198448	0.46556538651989
C	-0.08289159980396	4.18399948207815	0.37421054966099
C	0.45105700183681	5.52083187634949	0.47681119980759
C	1.80051305104806	5.40102367865908	0.53395328758891
C	3.37729970510827	3.44170667256468	0.49036442840105
C	3.69967851986117	2.08878385213340	0.42400476459955
C	3.51259398153342	-0.09060348178052	0.29119017390822
C	4.92317313208126	0.20355399743043	0.37383063314480
C	5.03867083571357	1.55215944257630	0.45601978308039
C	2.96610145396997	-1.36766955151321	0.19597878942074
C	1.61266139827145	-1.68484149609102	0.11443913749782
C	-0.56803689865762	-1.49117702917241	0.02313785293484
C	1.07873065617102	-3.02161880276234	0.01173326105740
C	-0.27077507452904	-2.90182339461384	-0.04524996308134
C	-1.84752940477694	-0.94244861085947	-0.00171676168154
C	-2.16982532932432	0.41049175225735	0.06459005158094
C	-1.98287450996867	2.58988256485616	0.19734388207446
C	-1.43634351517811	3.86693394953137	0.29257817151099
C	-3.50884961170764	0.94707322751101	0.03237212058320
C	-3.39342713134916	2.29568609686051	0.11448696102098
H	2.53951151237305	6.18324603608213	0.61640717194316
H	-0.14311213744145	6.42172183527438	0.49930953738452
H	-4.40858724252658	0.35543950242980	-0.04086290330700
H	-4.17906098481605	3.03545435050373	0.11909753637356
H	5.70878162270254	-0.53624222820203	0.36903210186662
H	5.93844338277820	2.14375162641525	0.52914255078510
H	1.67278257751461	-3.92258125066991	-0.01089362937318
H	-1.00965009715002	-3.68416324174073	-0.12767864908821
H	-2.13258460840235	4.69648136541795	0.30562352788457
H	4.20414638954949	4.13681446386712	0.56964835835064
H	3.66243362202855	-2.19713807159103	0.18281145923729
H	-2.67445962198885	-1.63745282316285	-0.08103205726779

**Molecule 7**	X	Y	Z
N	0.00306929203157	2.09025038939385	0.00280637734693
N	-2.02217701402833	0.00367524665081	0.00199071579780
N	2.02222764449151	-0.00235012189873	0.00311343780935
N	-0.00302514720916	-2.08892809680629	0.00130379139784
C	-1.11825453647958	2.87050259645016	0.00145142832907
C	1.12680296387394	2.86702222720281	0.00109213376991
H	0.00120006578671	1.07864620129983	0.00934927825736
C	0.68708141874084	4.22778263249457	-0.00643100900445
C	-0.67436693509620	4.22989560314264	0.00272205439625
C	-2.44002841611649	2.42813682773948	0.00983915695293
C	-2.84107539962996	1.08570483059574	0.01059961802454
C	-4.23921249316304	0.68028627583166	0.00287026102398
C	-4.24120444867964	-0.66626953935596	-0.00101852547719
C	-2.84429222033477	-1.07584293381617	-0.00763101175232
C	-2.44721552868200	-2.41946126305322	-0.00771213392199
C	-1.12675218478268	-2.86572787227499	0.00115997144442

C	1.11831464544172	-2.86914921605166	0.00281608683779
H	-0.00115366074884	-1.07731535426185	-0.00388720034447
C	-0.68696883514829	-4.22650541507757	-0.00136599562075
C	0.67446889386732	-4.22853590497143	0.00861753365142
C	2.44008854074853	-2.42680463934112	0.01165639546804
C	2.84113072888243	-1.08436166442364	0.01228902103977
C	2.84434720589735	1.07717140803578	-0.00622947455907
C	2.44728147686258	2.42078070989654	-0.00677223526928
C	4.24123695013448	0.66762008777896	0.00106062624935
C	4.23925535104704	-0.67893679646340	0.00521015702972
H	5.09225127015025	1.32918605505726	0.00469432900260
H	5.08831128578596	-1.34300322772316	0.00151621957039
H	1.32802440371805	-5.08489538021350	0.01852050789344
H	-1.34305661505106	-5.08093431576111	-0.00875635767935
H	-5.09214708028830	-1.32792421671142	0.00220601680277
H	-1.32789366434545	5.08630776533362	0.00870952295629
H	-5.08820496460140	1.34443077408419	-0.00114823080884
H	1.34323895038456	5.08213243976899	-0.01698778967142
C	-3.49687315161061	3.48248340968376	0.01944671847913
C	-4.25471276884167	3.71202747352452	1.16590066638243
C	-3.73911502156493	4.25202985081993	-1.11647673852723
C	-5.23695620187455	4.69308077863062	1.17668410983518
H	-4.06782973443855	3.11725497258100	2.05192596240685
C	-4.72359712465100	5.23118460199917	-1.10777523432276
H	-3.15462373434607	4.07458054087165	-2.01130069244300
C	-5.47428883612080	5.45423925832327	0.03924722209487
H	-5.81558868854778	4.86415127058852	2.07527936209419
H	-4.90636686309054	5.81748277227697	-1.99908442516288
H	-6.24108742756642	6.21786141951443	0.04687604686046
C	3.50720633950402	3.47196779088813	-0.02015075863742
C	3.75255137413916	4.24442565075954	1.11314233932546
C	4.26495278698812	3.69552996044240	-1.16784051200052
C	4.74007154852479	5.22049807640632	1.10058900916027
H	3.16810106962667	4.07176124628361	2.00892387335435
C	5.25014434617170	4.67355306826243	-1.18251699004659
H	4.07561966025464	3.09845069636815	-2.05179949792543
C	5.49067748459121	5.43753408569527	-0.04764367586526
H	4.92537107478962	5.80904966202027	1.98989323905847
H	5.82867381392441	4.83985664897826	-2.08207192840365
H	6.25987748948953	6.19870363591574	-0.05826312620336
C	3.49684824399215	-3.48116565929454	0.02311512003437
C	4.25485095509833	-3.70861470601657	1.16988598252202
C	3.73896152495152	-4.25279820806055	-1.11143342335254
C	5.23715808879263	-4.68957507599827	1.18232860041316
H	4.06805057418075	-3.11223909736553	2.05485473824547
C	4.72357077707417	-5.23181234238264	-1.10110833700449
H	3.15429022187389	-4.07709561078290	-2.00648178320761
C	5.47446828728200	-5.45269341275497	0.04619687131005
H	5.81589237377080	-4.85896311295786	2.08117623137477

H	4.90629333327271	-5.81969465260333	-1.99138578357649
H	6.24135549346659	-6.21621425835526	0.05511722240542
C	-3.50726459706697	-3.47058506862116	-0.02073411231027
C	-3.75398793619868	-4.24140881725679	1.11336851085766
C	-4.26377006158693	-3.69560529250371	-1.16894623276452
C	-4.74155121786693	-5.21743843757659	1.10102879167875
H	-3.17058730111664	-4.06747341237761	2.00958886180113
C	-5.24907241390051	-4.67352812768251	-1.18336589334812
H	-4.07334591289115	-3.09978475050295	-2.05351668798731
C	-5.49090009941160	-5.43597943913002	-0.04774443725072
H	-4.92791950090297	-5.80476025745435	1.99092102652918
H	-5.82665583159921	-4.84099805602617	-2.08331190434409
H	-6.26015438002447	-6.19709518768131	-0.05817300848221

S3 Calculated Vertical Excitaion Energies of B Band

Table S4: Calculated Vertical Excitaion Energies (EE) in nm and eV, Oscillator Strength (f), and their MO Designation and Corresponding Coefficients, of the B Band of Molecule **1**, based on BPE0/def2-TZVP/CPCM optimized geometry.

Functional	Electronic transition	EE (nm)	EE (eV)	f	MO designation (coefficient)
PBE0-DH	$S_0 \rightarrow S_4$	377.5	3.284	1.57	H-1→L (0.53), H→L+1 (0.45)
mPW2PLYP	$S_0 \rightarrow S_4$	391.8	3.164	1.54	H-1→L (0.55), H→L+1 (0.43)
B2PLYP	$S_0 \rightarrow S_4$	397.0	3.123	1.51	H-1→L (0.54), H→L+1 (0.43)
PBE-QIDH	$S_0 \rightarrow S_4$	386.2	3.210	1.58	H-1→L (0.58), H→L+1 (0.41)
B2GPPLYP	$S_0 \rightarrow S_4$	395.5	3.135	1.54	H-1→L (0.57), H→L+1 (0.41)
PBE0-2	$S_0 \rightarrow S_4$	394.7	3.141	2.00	H-1→L (0.59), H→L+1 (0.39)
RSX-0DH	$S_0 \rightarrow S_4$	360.8	3.436	1.76	H-1→L (0.57), H→L+1 (0.41)
ωB2PLYP	$S_0 \rightarrow S_4$	375.1	3.305	1.63	H-1→L (0.57), H→L+1 (0.41)
RSX-QIDH	$S_0 \rightarrow S_4$	373.8	3.317	1.65	H-1→L (0.58), H→L+1 (0.40)
ωB2GPPLYP	$S_0 \rightarrow S_4$	379.4	3.268	1.62	H-1→L (0.58), H→L+1 (0.40)
ωB88PP86	$S_0 \rightarrow S_4$	387.8	3.197	1.58	H-1→L (0.57), H→L+1 (0.40)
ωPBEPP86	$S_0 \rightarrow S_4$	391.3	3.168	1.57	H-1→L (0.58), H→L+1 (0.40)
DSD-BLYP	$S_0 \rightarrow S_4$	469.7	2.640	1.31	H-1→L (0.59), H→L+1 (0.39)
DSD-PBEP86	$S_0 \rightarrow S_4$	476.2	2.604	1.55	H-1→L (0.58), H→L+1 (0.40)
SCS/SOS-B2PLYP	$S_0 \rightarrow S_4$	365.3	3.394	1.64	H-1→L (0.54), H→L+1 (0.43)
SCS-B2GPPLYP	$S_0 \rightarrow S_4$	381.0	3.254	1.60	H-1→L (0.57), H→L+1 (0.41)
SOS-B2GPPLYP	$S_0 \rightarrow S_4$	386.0	3.212	1.58	H-1→L (0.57), H→L+1 (0.41)
SCS-PBE-QIDH	$S_0 \rightarrow S_4$	397.4	3.120	1.54	H-1→L (0.58), H→L+1 (0.41)
SOS-PBE-QIDH	$S_0 \rightarrow S_4$	398.1	3.115	1.53	H-1→L (0.58), H→L+1 (0.41)
SCS/SOS-ωB2PLYP	$S_0 \rightarrow S_4$	384.4	3.225	1.59	H-1→L (0.57), H→L+1 (0.41)
SCS-ωB2GPPLYP	$S_0 \rightarrow S_4$	399.6	3.102	1.54	H-1→L (0.58), H→L+1 (0.40)
SOS-ωB2GPPLYP	$S_0 \rightarrow S_4$	393.1	3.154	1.57	H-1→L (0.58), H→L+1 (0.40)
SCS-RSX-QIDH	$S_0 \rightarrow S_4$	417.4	2.970	1.47	H-1→L (0.58), H→L+1 (0.40)
SOS-RSX-QIDH	$S_0 \rightarrow S_4$	410.2	3.023	1.50	H-1→L (0.58), H→L+1 (0.40)
SCS-ωB88PP86	$S_0 \rightarrow S_4$	393.6	3.150	1.56	H-1→L (0.57), H→L+1 (0.41)
SOS-ωB88PP86	$S_0 \rightarrow S_4$	395.6	3.134	1.55	H-1→L (0.57), H→L+1 (0.41)
SCS-ωPBEPP86	$S_0 \rightarrow S_4$	414.1	2.994	1.49	H-1→L (0.58), H→L+1 (0.40)
SOS-ωPBEPP86	$S_0 \rightarrow S_4$	402.8	3.078	1.53	H-1→L (0.58), H→L+1 (0.40)

Table S5: Calculated Vertical Excitaion Energies (EE) in nm and eV, Oscillator Strength (f), and their MO Designation and Corresponding Coefficients, of the B Band of Molecule **2**, based on BPE0/def2-TZVP/CPCM optimized geometry.

Functional	Electronic transition	EE (nm)	EE (eV)	f	MO designation (coefficient)
PBE0-DH	$S_0 \to S_4$	379.6	3.266	1.55	H-1→L+1 (0.41), H→L (0.34)
mPW2PLYP	$S_0 \to S_4$	394.1	3.146	1.51	H-1→L+1 (0.43), H→L (0.32)
B2PLYP	$S_0 \to S_4$	399.3	3.105	1.49	H-1→L+1 (0.43), H→L (0.33)
PBE-QIDH	$S_0 \to S_4$	388.7	3.189	1.59	H-1→L+1 (0.45), H→L (0.30)
B2GPPLYP	$S_0 \to S_4$	398.1	3.115	1.52	H-1→L+1 (0.45), H→L (0.31)
PBE0-2	$S_0 \to S_4$	397.8	3.117	1.54	H-1→L+1 (0.46), H→L (0.29)
RSX-0DH	$S_0 \to S_4$	362.6	3.419	1.66	H-1→L+1 (0.44), H→L (0.31)
ωB2PLYP	$S_0 \to S_4$	377.2	3.287	1.61	H-1→L+1 (0.43), H→L (0.30)
RSX-QIDH	$S_0 \to S_4$	376.0	3.297	1.62	H-1→L+1 (0.45), H→L (0.29)
ωB2GPPLYP	$S_0 \to S_4$	381.7	3.248	1.60	H-1→L+1 (0.44), H→L (0.29)
ωB88PP86	$S_0 \to S_4$	390.5	3.175	1.56	H-1→L+1 (0.44), H→L (0.30)
ωPBEPP86	$S_0 \to S_4$	394.2	3.145	1.55	H-1→L+1 (0.44), H→L (0.30)
DSD-BLYP	$S_0 \to S_4$	476.7	2.601	1.29	H-1→L+1 (0.46), H→L (0.29)
DSD-PBEP86	$S_0 \to S_4$	483.1	2.567	1.26	H-1→L+1 (0.45), H→L (0.30)
SCS/SOS-B2PLYP	$S_0 \to S_4$	367.2	3.376	1.62	H-1→L+1 (0.43), H→L (0.33)
SCS-B2GPPLYP	$S_0 \to S_4$	383.3	3.234	1.58	H-1→L+1 (0.45), H→L (0.31)
SOS-B2GPPLYP	$S_0 \to S_4$	388.4	3.192	1.56	H-1→L+1 (0.45), H→L (0.31)
SCS-PBE-QIDH	$S_0 \to S_4$	400.3	3.097	1.51	H-1→L+1 (0.45), H→L (0.30)
SOS-PBE-QIDH	$S_0 \to S_4$	401.0	3.092	1.51	H-1→L+1 (0.45), H→L (0.30)
SCS/SOS-ωB2PLYP	$S_0 \to S_4$	386.9	3.205	1.57	H-1→L+1 (0.43), H→L (0.30)
SCS-ωB2GPPLYP	$S_0 \to S_4$	402.7	3.079	1.52	H-1→L+1 (0.44), H→L (0.29)
SOS-ωB2GPPLYP	$S_0 \to S_4$	395.8	3.132	1.54	H-1→L+1 (0.44), H→L (0.29)
SCS-RSX-QIDH	$S_0 \to S_4$	421.5	2.942	1.45	H-1→L+1 (0.45), H→L (0.29)
SOS-RSX-QIDH	$S_0 \to S_4$	413.8	2.997	1.48	H-1→L+1 (0.45), H→L (0.29)
SCS-ωB88PP86	$S_0 \to S_4$	396.5	3.127	1.54	H-1→L+1 (0.44), H→L (0.30)
SOS-ωB88PP86	$S_0 \to S_4$	398.5	3.112	1.53	H-1→L+1 (0.44), H→L (0.30)
SCS-ωPBEPP86	$S_0 \to S_4$	417.8	2.967	1.46	H-1→L+1 (0.44), H→L (0.30)
SOS-ωPBEPP86	$S_0 \to S_4$	405.9	3.054	1.51	H-1→L+1 (0.44), H→L (0.30)

Table S6: Calculated Vertical Excitaion Energies (EE) in nm and eV, Oscillator Strength (f), and their MO Designation and Corresponding Coefficients, of the B Band of Molecule **3**, based on BPE0/def2-TZVP/CPCM optimized geometry.

Functional	Electronic transition	EE (nm)	EE (eV)	f	MO designation (coefficient)
PBE0-DH	$S_0 \rightarrow S_4$	375.6	3.301	1.52	H-1→L+1 (0.56), H→L (0.42)
mPW2PLYP	$S_0 \rightarrow S_4$	390.0	3.179	1.49	H-1→L+1 (0.57), H→L (0.40)
B2PLYP	$S_0 \rightarrow S_4$	395.2	3.137	1.46	H-1→L+1 (0.57), H→L (0.40)
PBE-QIDH	$S_0 \rightarrow S_4$	384.5	3.225	1.54	H-1→L+1 (0.60), H→L (0.38)
B2GPPLYP	$S_0 \rightarrow S_4$	393.8	3.148	1.50	H-1→L+1 (0.60), H→L (0.38)
PBE0-2	$S_0 \rightarrow S_4$	393.4	3.152	1.52	H-1→L+1 (0.61), H→L (0.36)
RSX-0DH	$S_0 \rightarrow S_4$	358.1	3.462	1.64	H-1→L+1 (0.58), H→L (0.39)
ωB2PLYP	$S_0 \rightarrow S_4$	372.6	3.328	1.58	H-1→L+1 (0.59), H→L (0.38)
RSX-QIDH	$S_0 \rightarrow S_4$	371.5	3.337	1.60	H-1→L+1 (0.60), H→L (0.37)
ωB2GPPLYP	$S_0 \rightarrow S_4$	377.2	3.287	1.58	H-1→L+1 (0.60), H→L (0.37)
ωB88PP86	$S_0 \rightarrow S_4$	386.0	3.212	1.54	H-1→L+1 (0.59), H→L (0.38)
ωPBEPP86	$S_0 \rightarrow S_4$	389.7	3.181	1.53	H-1→L+1 (0.60), H→L (0.38)
DSD-BLYP	$S_0 \rightarrow S_4$	471.9	2.628	1.27	H-1→L+1 (0.61), H→L (0.27)
DSD-PBEP86	$S_0 \rightarrow S_4$	478.3	2.592	1.24	H-1→L+1 (0.61), H→L (0.37)
SCS/SOS-B2PLYP	$S_0 \rightarrow S_4$	363.1	3.415	1.59	H-1→L+1 (0.57), H→L (0.40)
SCS-B2GPPLYP	$S_0 \rightarrow S_4$	379.1	3.271	1.55	H-1→L+1 (0.59), H→L (0.38)
SOS-B2GPPLYP	$S_0 \rightarrow S_4$	384.2	3.227	1.53	H-1→L+1 (0.59), H→L (0.38)
SCS-PBE-QIDH	$S_0 \rightarrow S_4$	396.1	3.130	1.49	H-1→L+1 (0.60), H→L (0.38)
SOS-PBE-QIDH	$S_0 \rightarrow S_4$	396.8	3.124	1.49	H-1→L+1 (0.60), H→L (0.38)
SCS/SOS-ωB2PLYP	$S_0 \rightarrow S_4$	382.3	3.243	1.54	H-1→L+1 (0.59), H→L (0.38)
SCS-ωB2GPPLYP	$S_0 \rightarrow S_4$	398.4	3.112	1.50	H-1→L+1 (0.60), H→L (0.37)
SOS-ωB2GPPLYP	$S_0 \rightarrow S_4$	391.4	3.168	1.52	H-1→L+1 (0.60), H→L (0.37)
SCS-RSX-QIDH	$S_0 \rightarrow S_4$	417.3	2.971	1.43	H-1→L+1 (0.60), H→L (0.37)
SOS-RSX-QIDH	$S_0 \rightarrow S_4$	409.3	3.029	1.46	H-1→L+1 (0.60), H→L (0.37)
SCS-ωB88PP86	$S_0 \rightarrow S_4$	392.2	3.161	1.51	H-1→L+1 (0.59), H→L (0.38)
SOS-ωB88PP86	$S_0 \rightarrow S_4$	394.1	3.146	1.51	H-1→L+1 (0.59), H→L (0.38)
SCS-ωPBEPP86	$S_0 \rightarrow S_4$	413.8	2.997	1.44	H-1→L+1 (0.60), H→L (0.38)
SOS-ωPBEPP86	$S_0 \rightarrow S_4$	401.6	3.087	1.48	H-1→L+1 (0.60), H→L (0.38)

Table S7: Calculated Vertical Excitaion Energies (EE) in nm and eV, Oscillator Strength (f), and their MO Designation and Corresponding Coefficients, of the B Band of Molecule **4**, based on BPE0/def2-TZVP/CPCM optimized geometry.

Functional	Electronic transition	EE (nm)	EE (eV)	f	MO designation (coefficient)
PBE0-DH	$S_0 \rightarrow S_4$	386.4	3.209	1.68	H-1→L (0.62), H→L+1 (0.36)
mPW2PLYP	$S_0 \rightarrow S_4$	402.2	3.082	1.64	H-1→L (0.64), H→L+1 (0.35)
B2PLYP	$S_0 \rightarrow S_4$	408.1	3.038	1.61	H-1→L (0.63), H→L+1 (0.35)
PBE-QIDH	$S_0 \rightarrow S_4$	395.0	3.139	1.69	H-1→L (0.65), H→L+1 (0.33)
B2GPPLYP	$S_0 \rightarrow S_4$	405.5	3.058	1.64	H-1→L (0.65), H→L+1 (0.33)
PBE0-2	$S_0 \rightarrow S_4$	403.9	3.069	1.67	H-1→L (0.66), H→L+1 (0.32)
RSX-0DH	$S_0 \rightarrow S_4$	366.3	3.385	1.82	H-1→L (0.64), H→L+1 (0.34)
ωB2PLYP	$S_0 \rightarrow S_4$	382.3	3.243	1.75	H-1→L (0.64), H→L+1 (0.34)
RSX-QIDH	$S_0 \rightarrow S_4$	380.6	3.258	1.77	H-1→L (0.65), H→L+1 (0.33)
ωB2GPPLYP	$S_0 \rightarrow S_4$	386.9	3.204	1.74	H-1→L (0.65), H→L+1 (0.33)
ωB88PP86	$S_0 \rightarrow S_4$	396.8	3.125	1.69	H-1→L (0.65), H→L+1 (0.34)
ωPBEPP86	$S_0 \rightarrow S_4$	400.7	3.094	1.68	H-1→L (0.65), H→L+1 (0.33)
DSD-BLYP	$S_0 \rightarrow S_4$	490.1	2.530	1.37	H-1→L (0.66), H→L+1 (0.32)
DSD-PBEP86	$S_0 \rightarrow S_4$	498.6	2.487	1.34	H-1→L (0.66), H→L+1 (0.33)
SCS/SOS-B2PLYP	$S_0 \rightarrow S_4$	372.1	3.332	1.76	H-1→L (0.63), H→L+1 (0.35)
SCS-B2GPPLYP	$S_0 \rightarrow S_4$	388.4	3.192	1.71	H-1→L (0.65), H→L+1 (0.33)
SOS-B2GPPLYP	$S_0 \rightarrow S_4$	393.9	3.148	1.69	H-1→L (0.65), H→L+1 (0.33)
SCS-PBE-QIDH	$S_0 \rightarrow S_4$	406.8	3.047	1.64	H-1→L (0.65), H→L+1 (0.33)
SOS-PBE-QIDH	$S_0 \rightarrow S_4$	407.3	3.044	1.64	H-1→L (0.65), H→L+1 (0.33)
SCS/SOS-ωB2PLYP	$S_0 \rightarrow S_4$	391.9	3.163	1.71	H-1→L (0.64), H→L+1 (0.34)
SCS-ωB2GPPLYP	$S_0 \rightarrow S_4$	409.3	3.029	1.64	H-1→L (0.65), H→L+1 (0.33)
SOS-ωB2GPPLYP	$S_0 \rightarrow S_4$	401.2	3.090	1.68	H-1→L (0.65), H→L+1 (0.33)
SCS-RSX-QIDH	$S_0 \rightarrow S_4$	431.3	2.875	1.56	H-1→L (0.65), H→L+1 (0.33)
SOS-RSX-QIDH	$S_0 \rightarrow S_4$	420.1	2.951	1.60	H-1→L (0.65), H→L+1 (0.33)
SCS-ωB88PP86	$S_0 \rightarrow S_4$	402.8	3.078	1.67	H-1→L (0.65), H→L+1 (0.34)
SOS-ωB88PP86	$S_0 \rightarrow S_4$	404.4	3.066	1.66	H-1→L (0.65), H→L+1 (0.34)
SCS-ωPBEPP86	$S_0 \rightarrow S_4$	427.0	2.904	1.57	H-1→L (0.65), H→L+1 (0.33)
SOS-ωPBEPP86	$S_0 \rightarrow S_4$	412.3	3.007	1.63	H-1→L (0.65), H→L+1 (0.33)

Table S8: Calculated Vertical Excitaion Energies (EE) in nm and eV, Oscillator Strength (f), and their MO Designation and Corresponding Coefficients, of the B Band of Molecule **5**, based on BPE0/def2-TZVP/CPCM optimized geometry.

Functional	Electronic transition	EE (nm)	EE (eV)	f	MO designation (coefficient)
PBE0-DH	$S_0 \rightarrow S_3$	388.6	3.190	1.66	H-1→L (0.61), H→L+1 (0.34)
mPW2PLYP	$S_0 \rightarrow S_3$	404.9	3.062	1.60	H-1→L (0.64), H→L+1 (0.33)
B2PLYP	$S_0 \rightarrow S_3$	410.8	3.018	1.58	H-1→L (0.63), H→L+1 (0.33)
PBE-QIDH	$S_0 \rightarrow S_3$	397.9	3.116	1.65	H-1→L (0.66), H→L+1 (0.32)
B2GPPLYP	$S_0 \rightarrow S_3$	408.7	3.034	1.61	H-1→L (0.66), H→L+1 (0.32)
PBE0-2	$S_0 \rightarrow S_3$	407.7	3.041	1.63	H-1→L (0.67), H→L+1 (0.30)
RSX-0DH	$S_0 \rightarrow S_3$	368.3	3.366	1.78	H-1→L (0.63), H→L+1 (0.33)
ωB2PLYP	$S_0 \rightarrow S_3$	384.9	3.221	1.71	H-1→L (0.65), H→L+1 (0.33)
RSX-QIDH	$S_0 \rightarrow S_3$	383.2	3.236	1.73	H-1→L (0.65), H→L+1 (0.32)
ωB2GPPLYP	$S_0 \rightarrow S_3$	389.8	3.181	1.70	H-1→L (0.66), H→L+1 (0.32)
ωB88PP86	$S_0 \rightarrow S_3$	400.2	3.098	1.65	H-1→L (0.65), H→L+1 (0.32)
ωPBEPP86	$S_0 \rightarrow S_3$	404.4	3.066	1.64	H-1→L (0.66), H→L+1 (0.32)
DSD-BLYP	$S_0 \rightarrow S_3$	500.2	2.479	1.32	H-1→L (0.67), H→L+1 (0.30)
DSD-PBEP86	$S_0 \rightarrow S_3$	508.4	2.439	1.30	H-1→L (0.67), H→L+1 (0.30)
SCS/SOS-B2PLYP	$S_0 \rightarrow S_3$	374.2	3.313	1.73	H-1→L (0.63), H→L+1 (0.33)
SCS-B2GPPLYP	$S_0 \rightarrow S_3$	391.3	3.168	1.68	H-1→L (0.66), H→L+1 (0.32)
SOS-B2GPPLYP	$S_0 \rightarrow S_3$	397.0	3.123	1.65	H-1→L (0.66), H→L+1 (0.32)
SCS-PBE-QIDH	$S_0 \rightarrow S_3$	410.5	3.020	1.60	H-1→L (0.66), H→L+1 (0.32)
SOS-PBE-QIDH	$S_0 \rightarrow S_3$	411.0	3.017	1.60	H-1→L (0.66), H→L+1 (0.32)
SCS/SOS-ωB2PLYP	$S_0 \rightarrow S_3$	395.2	3.138	1.66	H-1→L (0.65), H→L+1 (0.33)
SCS-ωB2GPPLYP	$S_0 \rightarrow S_3$	413.4	2.999	1.60	H-1→L (0.66), H→L+1 (0.32)
SOS-ωB2GPPLYP	$S_0 \rightarrow S_3$	405.0	3.061	1.63	H-1→L (0.66), H→L+1 (0.32)
SCS-RSX-QIDH	$S_0 \rightarrow S_3$	436.4	2.841	1.52	H-1→L (0.65), H→L+1 (0.32)
SOS-RSX-QIDH	$S_0 \rightarrow S_3$	424.8	2.919	1.56	H-1→L (0.65), H→L+1 (0.32)
SCS-ωB88PP86	$S_0 \rightarrow S_3$	406.6	3.049	1.63	H-1→L (0.65), H→L+1 (0.32)
SOS-ωB88PP86	$S_0 \rightarrow S_3$	408.2	3.037	1.62	H-1→L (0.65), H→L+1 (0.32)
SCS-ωPBEPP86	$S_0 \rightarrow S_3$	431.8	2.871	1.54	H-1→L (0.66), H→L+1 (0.32)
SOS-ωPBEPP86	$S_0 \rightarrow S_3$	416.5	2.977	1.59	H-1→L (0.66), H→L+1 (0.32)

Table S9: Calculated Vertical Excitaion Energies (EE) in nm and eV, Oscillator Strength (f), and their MO Designation and Corresponding Coefficients, of the B Band of Molecule **6**, based on BPE0/def2-TZVP/CPCM optimized geometry.

Functional	Electronic transition	EE (nm)	EE (eV)	f	MO designation (coefficient)
PBE0-DH	$S_0 \rightarrow S_3$	383.8	3.230	1.63	H-1→L (0.63), H→L+1 (0.32)
mPW2PLYP	$S_0 \rightarrow S_3$	399.9	3.101	1.58	H-1→L (0.65), H→L+1 (0.31)
B2PLYP	$S_0 \rightarrow S_3$	405.7	3.056	1.55	H-1→L (0.65), H→L+1 (0.31)
PBE-QIDH	$S_0 \rightarrow S_3$	392.9	3.156	1.63	H-1→L (0.66), H→L+1 (0.29)
B2GPPLYP	$S_0 \rightarrow S_3$	403.5	3.073	1.58	H-1→L (0.66), H→L+1 (0.30)
PBE0-2	$S_0 \rightarrow S_3$	402.4	3.081	1.61	H-1→L (0.67), H→L+1 (0.28)
RSX-0DH	$S_0 \rightarrow S_4$	363.2	3.414	1.76	H-1→L+1 (0.63), H→L (0.30)
ωB2PLYP	$S_0 \rightarrow S_4$	379.5	3.267	1.69	H-1→L+1 (0.65), H→L (0.30)
RSX-QIDH	$S_0 \rightarrow S_4$	378.1	3.279	1.71	H-1→L+1 (0.65), H→L (0.30)
ωB2GPPLYP	$S_0 \rightarrow S_4$	384.5	3.224	1.68	H-1→L+1 (0.66), H→L (0.30)
ωB88PP86	$S_0 \rightarrow S_4$	394.8	3.140	1.63	H-1→L+1 (0.52), H→L (0.24)
ωPBEPP86	$S_0 \rightarrow S_4$	399.0	3.107	1.62	H-1→L+1 (0.53), H→L (0.24)
DSD-BLYP	$S_0 \rightarrow S_3$	493.5	2.512	1.31	H-1→L (0.67), H→L+1 (0.29)
DSD-PBEP86	$S_0 \rightarrow S_3$	501.7	2.471	1.28	H-1→L (0.67), H→L+1 (0.29)
SCS/SOS-B2PLYP	$S_0 \rightarrow S_3$	369.3	3.357	1.70	H-1→L (0.65), H→L+1 (0.31)
SCS-B2GPPLYP	$S_0 \rightarrow S_3$	386.3	3.210	1.65	H-1→L (0.66), H→L+1 (0.29)
SOS-B2GPPLYP	$S_0 \rightarrow S_3$	392.0	3.163	1.63	H-1→L (0.66), H→L+1 (0.29)
SCS-PBE-QIDH	$S_0 \rightarrow S_3$	405.5	3.058	1.58	H-1→L (0.66), H→L+1 (0.29)
SOS-PBE-QIDH	$S_0 \rightarrow S_3$	406.0	3.054	1.58	H-1→L (0.66), H→L+1 (0.29)
SCS/SOS-ωB2PLYP	$S_0 \rightarrow S_4$	389.9	3.180	1.64	H-1→L+1 (0.65), H→L (0.30)
SCS-ωB2GPPLYP	$S_0 \rightarrow S_4$	408.3	3.037	1.58	H-1→L+1 (0.66), H→L (0.30)
SOS-ωB2GPPLYP	$S_0 \rightarrow S_4$	399.7	3.102	1.62	H-1→L+1 (0.66), H→L (0.30)
SCS-RSX-QIDH	$S_0 \rightarrow S_4$	431.4	2.874	1.50	H-1→L+1 (0.65), H→L (0.30)
SOS-RSX-QIDH	$S_0 \rightarrow S_4$	419.6	2.955	1.54	H-1→L+1 (0.65), H→L (0.30)
SCS-ωB88PP86	$S_0 \rightarrow S_4$	401.5	3.088	1.60	H-1→L+1 (0.65), H→L (0.30)
SOS-ωB88PP86	$S_0 \rightarrow S_4$	403.0	3.076	1.60	H-1→L+1 (0.52), H→L (0.24)
SCS-ωPBEPP86	$S_0 \rightarrow S_3$	426.9	2.904	1.51	H-1→L (0.66), H→L+1 (0.30)
SOS-ωPBEPP86	$S_0 \rightarrow S_4$	411.4	3.014	1.57	H-1→L+1 (0.53), H→L (0.24)

Table S10: Calculated Vertical Excitaion Energies (EE) in nm and eV, Oscillator Strength (f), and their MO Designation and Corresponding Coefficients, of the B Band of Molecule **7**, based on BPE0/def2-TZVP/CPCM optimized geometry.

Functional	Electronic transition	EE (nm)	EE (eV)	f	MO designation (coefficient)
PBE0-DH	$S_0 \rightarrow S_4$	401.2	3.090	1.98	H-1→L (0.54), H →L+1(0.44)
mPW2PLYP	$S_0 \rightarrow S_4$	416.3	2.978	1.94	H-1→L (0.51), H →L+1(0.47)
B2PLYP	$S_0 \rightarrow S_4$	421.6	2.941	1.91	H-1→L (0.52), H →L+1(0.46)
PBE-QIDH	$S_0 \rightarrow S_4$	409.6	3.027	2.00	H →L +1 (0.50), H-1→L (0.48)
B2GPPLYP	$S_0 \rightarrow S_4$	419.6	2.955	1.95	H →L +1 (0.50), H-1→L (0.48)
PBE0-2	$S_0 \rightarrow S_4$	418.2	2.964	1.99	H-1→L +1 (0.53), H →L (0.45)
RSX-0DH	$S_0 \rightarrow S_4$	381.6	3.249	2.13	H →L +1 (0.45), H-1→L (0.42)
ωB2PLYP	$S_0 \rightarrow S_4$	397.0	3.123	2.06	H →L +1 (0.50), H-1→L (0.47)
RSX-QIDH	$S_0 \rightarrow S_4$	395.4	3.136	2.09	H-1→L +1 (0.50), H →L (0.44)
ωB2GPPLYP	$S_0 \rightarrow S_4$	401.6	3.087	2.05	H-1→L +1 (0.52), H →L (0.46)
ωB88PP86	$S_0 \rightarrow S_4$	410.6	3.020	2.00	H →L +1 (0.50), H-1→L (0.47)
ωPBEPP86	$S_0 \rightarrow S_4$	414.3	2.992	1.99	H-1→L +1 (0.51), H →L (0.46)
DSD-BLYP	$S_0 \rightarrow S_4$	496.8	2.496	1.67	H-1→L +1 (0.52), H →L (0.46)
DSD-PBEP86	$S_0 \rightarrow S_4$	503.8	2.461	1.64	H-1→L +1 (0.51), H →L (0.47)
SCS/SOS-B2PLYP	$S_0 \rightarrow S_4$	384.9	3.221	2.09	H-1→L (0.52), H →L+1(0.46)
SCS-B2GPPLYP	$S_0 \rightarrow S_4$	402.6	3.080	2.03	H →L +1 (0.50), H-1→L (0.48)
SOS-B2GPPLYP	$S_0 \rightarrow S_4$	408.6	3.034	2.00	H →L +1 (0.50), H-1→L (0.48)
SCS-PBE-QIDH	$S_0 \rightarrow S_4$	422.3	2.936	1.95	H →L +1 (0.50), H-1→L (0.48)
SOS-PBE-QIDH	$S_0 \rightarrow S_4$	422.8	2.933	1.94	H →L +1 (0.50), H-1→L (0.48)
SCS/SOS-ωB2PLYP	$S_0 \rightarrow S_4$	407.7	3.041	2.00	H →L +1 (0.50), H-1→L (0.47)
SCS-ωB2GPPLYP	$S_0 \rightarrow S_4$	426.7	2.906	1.93	H-1→L (0.48), H →L+1(0.47)
SOS-ωB2GPPLYP	$S_0 \rightarrow S_4$	417.4	2.970	1.98	H-1→L +1 (0.52), H →L (0.46)
SCS-RSX-QIDH	$S_0 \rightarrow S_4$	451.1	2.748	1.83	H-1→L +1 (0.50), H →L (0.44)
SOS-RSX-QIDH	$S_0 \rightarrow S_4$	437.5	2.834	1.89	H-1→L +1 (0.50), H →L (0.44)
SCS-ωB88PP86	$S_0 \rightarrow S_4$	418.9	2.960	1.96	H →L +1 (0.50), H-1→L (0.47)
SOS-ωB88PP86	$S_0 \rightarrow S_4$	420.3	2.950	1.96	H →L +1 (0.50), H-1→L (0.47)
SCS-ωPBEPP86	$S_0 \rightarrow S_4$	445.9	2.780	1.85	H-1→L +1 (0.51), H →L (0.46)
SOS-ωPBEPP86	$S_0 \rightarrow S_4$	428.8	2.891	1.93	H-1→L +1 (0.51), H →L (0.46)

Table S11: Calculated Vertical Excitaion Energies (EE) in nm and eV, Oscillator Strength (f), and their MO Designation and Corresponding Coefficients, of the B Band of Molecule **8**, based on BPE0/def2-TZVP/CPCM optimized geometry.

Functional	Electronic transition	EE (nm)	EE (eV)	f	MO designation (coefficient)
PBE0-DH	$S_0 \rightarrow S_3$	403.90	3.070	1.94	H-1→L (0.46), H→ L+1(0.39)
mPW2PLYP	$S_0 \rightarrow S_3$	419.00	2.958	1.89	H-1→L (0.42), H→ L+1(0.39)
B2PLYP	$S_0 \rightarrow S_3$	424.60	2.920	1.86	H-1→L (0.43), H→ L+1(0.39)
PBE-QIDH	$S_0 \rightarrow S_3$	412.40	3.006	1.96	H →L+1 (0.33), H-1→L (0.30)
B2GPPLYP	$S_0 \rightarrow S_3$	423.00	2.933	1.91	H-1→L+1 (0.29), H→ L(0.27)
PBE0-2	$S_0 \rightarrow S_3$	421.50	2.941	1.95	H-1→L (0.30), H→ L+1(0.25)
RSX-0DH	$S_0 \rightarrow S_3$	383.60	3.232	2.09	H-1→L (0.28), H-1→L+1(0.23)
ωB2PLYP	$S_0 \rightarrow S_3$	399.00	3.105	2.00	H-1→L+1 (0.38), H→ L (0.35)
RSX-QIDH	$S_0 \rightarrow S_3$	397.90	3.116	2.05	H-1→L+1 (0.31), H→ L (0.26)
ωB2GPPLYP	$S_0 \rightarrow S_3$	404.10	3.068	2.00	H-1→L+1 (0.34), H→ L (0.29)
ωB88PP86	$S_0 \rightarrow S_3$	413.50	2.999	1.96	H-1→L+1 (0.38), H→ L (0.34)
ωPBEPP86	$S_0 \rightarrow S_3$	417.40	2.971	1.95	H-1→L+1 (0.36), H→ L (0.31)
DSD-BLYP	$S_0 \rightarrow S_3$	503.00	2.463	1.62	H-1→L+1 (0.28), H-1→L (0.26)
DSD-PBEP86	$S_0 \rightarrow S_3$	510.00	2.430	1.59	H-1→L+1 (0.32), H→ L (0.28)
SCS/SOS-B2PLYP	$S_0 \rightarrow S_3$	387.30	3.202	2.04	H-1→L (0.43), H→ L+1(0.39)
SCS-B2GPPLYP	$S_0 \rightarrow S_3$	405.30	3.059	1.99	H-1→L+1 (0.29), H→ L (0.27)
SOS-B2GPPLYP	$S_0 \rightarrow S_3$	411.40	3.013	1.96	H-1→L+1 (0.29), H→ L (0.27)
SCS-PBE-QIDH	$S_0 \rightarrow S_3$	425.50	2.914	1.90	H-1→L+1 (0.33), H→ L (0.30)
SOS-PBE-QIDH	$S_0 \rightarrow S_3$	426.00	2.910	1.90	H-1→L+1 (0.33), H→ L (0.30)
SCS/SOS-ωB2PLYP	$S_0 \rightarrow S_3$	410.40	3.021	1.96	H-1→L+1 (0.37), H→ L (0.34)
SCS-ωB2GPPLYP	$S_0 \rightarrow S_3$	429.90	2.884	1.89	H-1→L+1 (0.33), H→ L (0.28)
SOS-ωB2GPPLYP	$S_0 \rightarrow S_3$	420.40	2.950	1.93	H-1→L+1 (0.33), H→ L (0.28)
SCS-RSX-QIDH	$S_0 \rightarrow S_3$	455.40	2.723	1.79	H-1→L+1 (0.31), H→ L (0.26)
SOS-RSX-QIDH	$S_0 \rightarrow S_3$	441.10	2.811	1.85	H-1→L+1 (0.31), H→ L (0.26)
SCS-ωB88PP86	$S_0 \rightarrow S_3$	422.00	2.938	1.92	H-1→L+1 (0.38), H→ L (0.34)
SOS-ωB88PP86	$S_0 \rightarrow S_3$	423.40	2.928	1.91	H-1→L+1 (0.38), H→ L (0.34)
SCS-ωPBEPP86	$S_0 \rightarrow S_3$	449.90	2.756	1.81	H-1→L+1 (0.36), H→ L (0.31)
SOS-ωPBEPP86	$S_0 \rightarrow S_3$	432.10	2.869	1.88	H-1→L+1 (0.36), H→ L (0.31)

Table S12: Calculated Vertical Excitaion Energies (EE) in nm and eV, Oscillator Strength (f), and their MO Designation and Corresponding Coefficients, of the B Band of Molecule **9**, based on BPE0/def2-TZVP/CPCM optimized geometry.

Functional	Electronic transition	EE (nm)	EE (eV)	f	MO designation (coefficient)
PBE0-DH	$S_0 \rightarrow S_4$	399.9	3.101	1.92	H-1→L (0.44), H→L+1 (0.41)
mPW2PLYP	$S_0 \rightarrow S_4$	415.0	2.987	1.87	H-1→L (0.40), H→L+1 (0.39)
B2PLYP	$S_0 \rightarrow S_4$	420.5	2.948	1.84	H-1→L (0.40), H→L+1 (0.40)
PBE-QIDH	$S_0 \rightarrow S_4$	408.2	3.038	1.95	H-1→L (0.49), H→L+1 (0.41)
B2GPPLYP	$S_0 \rightarrow S_4$	418.3	2.964	1.89	H-1→L (0.46), H→L+1 (0.40)
PBE0-2	$S_0 \rightarrow S_4$	417.0	2.973	1.93	H-1→L (0.51), H→L+1 (0.39)
RSX-0DH	$S_0 \rightarrow S_4$	378.9	3.272	2.07	H-1→L (0.50), H→L+1 (0.43)
ωB2PLYP	$S_0 \rightarrow S_4$	394.5	3.143	2.00	H-1→L (0.49), H→L+1 (0.41)
RSX-QIDH	$S_0 \rightarrow S_4$	393.2	3.153	2.03	H-1→L (0.51), H→L+1 (0.41)
ωB2GPPLYP	$S_0 \rightarrow S_4$	399.4	3.104	2.00	H-1→L (0.50), H→L+1 (0.40)
ωB88PP86	$S_0 \rightarrow S_4$	408.9	3.032	1.94	H-1→L (0.49), H→L+1 (0.41)
ωPBEPP86	$S_0 \rightarrow S_4$	412.9	3.003	1.94	H-1→L (0.50), H→L+1 (0.41)
DSD-BLYP	$S_0 \rightarrow S_4$	499.0	2.484	1.61	H-1→L (0.49), H→L+1 (0.38)
DSD-PBEP86	$S_0 \rightarrow S_4$	506.3	2.449	1.58	H-1→L (0.48), H→L+1 (0.39)
SCS/SOS-B2PLYP	$S_0 \rightarrow S_4$	383.1	3.237	2.02	H-1→L (0.40), H→L+1 (0.40)
SCS-B2GPPLYP	$S_0 \rightarrow S_4$	400.9	3.093	1.97	H-1→L (0.46), H→L+1 (0.40)
SOS-B2GPPLYP	$S_0 \rightarrow S_4$	407.0	3.046	1.94	H-1→L (0.46), H→L+1 (0.40)
SCS-PBE-QIDH	$S_0 \rightarrow S_4$	421.2	2.944	1.89	H-1→L (0.49), H→L+1 (0.41)
SOS-PBE-QIDH	$S_0 \rightarrow S_4$	421.6	2.941	1.88	H-1→L (0.49), H→L+1 (0.41)
SCS/SOS-ωB2PLYP	$S_0 \rightarrow S_4$	405.5	3.058	1.94	H-1→L (0.49), H→L+1 (0.42)
SCS-ωB2GPPLYP	$S_0 \rightarrow S_4$	425.2	2.916	1.87	H-1→L (0.50), H→L+1 (0.40)
SOS-ωB2GPPLYP	$S_0 \rightarrow S_4$	415.5	2.984	1.92	H-1→L (0.50), H→L+1 (0.40)
SCS-RSX-QIDH	$S_0 \rightarrow S_4$	451.1	2.749	1.77	H-1→L (0.51), H→L+1 (0.41)
SOS-RSX-QIDH	$S_0 \rightarrow S_4$	436.3	2.842	1.83	H-1→L (0.51), H→L+1 (0.41)
SCS-ωB88PP86	$S_0 \rightarrow S_4$	417.5	2.970	1.90	H-1→L (0.49), H→L+1 (0.41)
SOS-ωB88PP86	$S_0 \rightarrow S_4$	418.7	2.961	1.90	H-1→L (0.49), H→L+1 (0.41)
SCS-ωPBEPP86	$S_0 \rightarrow S_4$	445.7	2.782	1.79	H-1→L (0.50), H→L+1 (0.41)
SOS-ωPBEPP86	$S_0 \rightarrow S_4$	427.5	2.900	1.87	H-1→L (0.50), H→L+1 (0.41)

Table S13: Calculated Vertical Excitaion Energies (EE) in nm and eV, Oscillator Strength (f), and their MO Designation and Corresponding Coefficients, of the B Band of Molecule **10**, based on BPE0/def2-TZVP/CPCM optimized geometry.

Functional	Electronic transition	EE (nm)	EE (eV)	f	MO designation (coefficient)
PBE0-DH	$S_0 \rightarrow S_4$	398.4	3.112	1.94	H-1→L+1 (0.49), H→L (0.48)
mPW2PLYP	$S_0 \rightarrow S_4$	413.5	2.999	1.90	H-1→L+1 (0.51), H→L (0.46)
B2PLYP	$S_0 \rightarrow S_4$	419.0	2.959	1.87	H-1→L+1 (0.51), H→L (0.47)
PBE-QIDH	$S_0 \rightarrow S_4$	382.4	3.050	1.97	H-1→L+1 (0.54), H→L (0.43)
B2GPPLYP	$S_0 \rightarrow S_4$	416.7	2.975	1.92	H-1→L+1 (0.54), H→L (0.44)
PBE0-2	$S_0 \rightarrow S_4$	415.2	2.986	1.96	H-1→L+1 (0.56), H→L (0.41)
RSX-0DH	$S_0 \rightarrow S_4$	378.9	3.273	2.10	H-1→L+1 (0.53), H→L (0.42)
ωB2PLYP	$S_0 \rightarrow S_4$	394.5	3.143	2.03	H-1→L+1 (0.54), H→L (0.43)
RSX-QIDH	$S_0 \rightarrow S_4$	382.4	3.158	2.10	H-1→L+1 (0.53), H→L (0.42)
ωB2GPPLYP	$S_0 \rightarrow S_4$	398.9	3.108	2.03	H-1→L+1 (0.55), H→L (0.42)
ωB88PP86	$S_0 \rightarrow S_4$	382.4	3.038	1.97	H-1→L+1 (0.54), H→L (0.43)
ωPBEPP86	$S_0 \rightarrow S_4$	382.4	3.011	1.96	H-1→L+1 (0.55), H→L (0.42)
DSD-BLYP	$S_0 \rightarrow S_4$	495.4	2.503	1.63	H-1→L+1 (0.56), H→L (0.41)
DSD-PBEP86	$S_0 \rightarrow S_4$	503.1	2.464	1.61	H-1→L+1 (0.55), H→L (0.45)
SCS/SOS-B2PLYP	$S_0 \rightarrow S_4$	382.0	3.245	2.05	H-1→L+1 (0.51), H→L (0.47)
SCS-B2GPPLYP	$S_0 \rightarrow S_4$	399.5	3.104	2.00	H-1→L+1 (0.54), H→L (0.44)
SOS-B2GPPLYP	$S_0 \rightarrow S_4$	405.5	3.058	1.97	H-1→L+1 (0.54), H→L (0.44)
SCS-PBE-QIDH	$S_0 \rightarrow S_4$	419.2	2.958	1.92	H-1→L+1 (0.54), H→L (0.43)
SOS-PBE-QIDH	$S_0 \rightarrow S_4$	419.7	2.954	1.91	H-1→L+1 (0.54), H→L (0.43)
SCS/SOS-ωB2PLYP	$S_0 \rightarrow S_4$	382.4	3.061	1.98	H-1→L+1 (0.54), H→L (0.43)
SCS-ωB2GPPLYP	$S_0 \rightarrow S_4$	382.4	2.925	1.91	H-1→L+1 (0.55), H→L (0.42)
SOS-ωB2GPPLYP	$S_0 \rightarrow S_4$	382.4	2.990	1.95	H-1→L+1 (0.55), H→L (0.42)
SCS-RSX-QIDH	$S_0 \rightarrow S_4$	382.4	2.765	1.80	H-1→L+1 (0.55), H→L (0.41)
SOS-RSX-QIDH	$S_0 \rightarrow S_4$	382.4	2.853	1.86	H-1→L+1 (0.55), H→L (0.41)
SCS-ωB88PP86	$S_0 \rightarrow S_4$	382.4	2.979	1.93	H-1→L+1 (0.54), H→L (0.43)
SOS-ωB88PP86	$S_0 \rightarrow S_4$	382.4	2.969	1.93	H-1→L+1 (0.54), H→L (0.43)
SCS-ωPBEPP86	$S_0 \rightarrow S_4$	382.4	2.798	1.82	H-1→L+1 (0.55), H→L (0.42)
SOS-ωPBEPP86	$S_0 \rightarrow S_4$	382.4	2.911	1.90	H-1→L+1 (0.55), H→L (0.42)

Table S14: Calculated Vertical Excitaion Energies (EE) in nm and eV, Oscillator Strength (f), and their MO Designation and Corresponding Coefficients, of the B Band of Molecule **11**, based on BPE0/def2-TZVP/CPCM optimized geometry.

Functional	Electronic transition	EE (nm)	EE (eV)	f	MO designation (coefficient)
PBE0-DH	$S_0 \to S_3$	423.2	2.929	1.65	H-1→L (0.67), H→L+1 (0.29)
mPW2PLYP	$S_0 \to S_3$	437.5	2.834	1.64	H-1→L (0.66), H→L+1 (0.31)
B2PLYP	$S_0 \to S_3$	442.8	2.800	1.61	H-1→L (0.66), H→L+1 (0.30)
PBE-QIDH	$S_0 \to S_3$	430.4	2.881	1.73	H-1→L (0.62), H→L+1 (0.35)
B2GPPLYP	$S_0 \to S_3$	440.0	2.818	1.68	H-1→L (0.62), H→L+1 (0.34)
PBE0-2	$S_0 \to S_3$	438.0	2.831	1.72	H-1→L (0.58), H→L+1 (0.37)
RSX-0DH	$S_0 \to S_3$	404.3	3.067	1.81	H-1→L (0.59), H→L+1 (0.34)
ωB2PLYP	$S_0 \to S_3$	418.6	2.962	1.76	H-1→L (0.59), H→L+1 (0.35)
RSX-QIDH	$S_0 \to S_3$	417.1	2.972	1.79	H-1→L (0.57), H→L+1 (0.36)
ωB2GPPLYP	$S_0 \to S_3$	422.9	2.932	1.76	H-1→L (0.57), H→L+1 (0.36)
ωB88PP86	$S_0 \to S_3$	430.6	2.879	1.72	H-1→L (0.60), H→L+1 (0.35)
ωPBEPP86	$S_0 \to S_3$	434.0	2.857	1.72	H-1→L (0.59), H→L+1 (0.36)
DSD-BLYP	$S_0 \to S_3$	507.9	2.441	1.49	H-1→L (0.59), H→L+1 (0.37)
DSD-PBEP86	$S_0 \to S_3$	514.4	2.410	1.46	H-1→L (0.60), H→L+1 (0.36)
SCS/SOS-B2PLYP	$S_0 \to S_3$	404.3	3.066	1.77	H-1→L (0.66), H→L+1 (0.30)
SCS-B2GPPLYP	$S_0 \to S_3$	424.5	2.921	1.74	H-1→L (0.62), H→L+1 (0.34)
SOS-B2GPPLYP	$S_0 \to S_3$	431.2	2.875	1.72	H-1→L (0.62), H→L+1 (0.34)
SCS-PBE-QIDH	$S_0 \to S_3$	446.1	2.779	1.67	H-1→L (0.62), H→L+1 (0.34)
SOS-PBE-QIDH	$S_0 \to S_3$	447.6	2.770	1.66	H-1→L (0.62), H→L+1 (0.35)
SCS/SOS-ωB2PLYP	$S_0 \to S_3$	433.3	2.862	1.70	H-1→L (0.59), H→L+1 (0.35)
SCS-ωB2GPPLYP	$S_0 \to S_3$	453.4	2.734	1.64	H-1→L (0.57), H→L+1 (0.36)
SOS-ωB2GPPLYP	$S_0 \to S_3$	444.3	2.791	1.68	H-1→L (0.57), H→L+1 (0.36)
SCS-RSX-QIDH	$S_0 \to S_3$	476.3	2.603	1.57	H-1→L (0.57), H→L+1 (0.36)
SOS-RSX-QIDH	$S_0 \to S_3$	468.0	2.649	1.60	H-1→L (0.57), H→L+1 (0.36)
SCS-ωB88PP86	$S_0 \to S_3$	443.6	2.795	1.67	H-1→L (0.60), H→L+1 (0.35)
SOS-ωB88PP86	$S_0 \to S_3$	446.2	2.779	1.66	H-1→L (0.60), H→L+1 (0.35)
SCS-ωPBEPP86	$S_0 \to S_3$	472.4	2.625	1.58	H-1→L (0.59), H→L+1 (0.36)
SOS-ωPBEPP86	$S_0 \to S_3$	456.1	2.719	1.64	H-1→L (0.59), H→L+1 (0.36)

Table S15: Calculated Vertical Excitaion Energies (EE) in nm and eV, Oscillator Strength (f), and their MO Designation and Corresponding Coefficients, of the B Band of Molecule **12**, based on BPE0/def2-TZVP/CPCM optimized geometry.

Functional	Electronic transition	EE (nm)	EE (eV)	f	MO designation (coefficient)
PBE0-DH	$S_0 \rightarrow S_3$	405.6	3.057	1.82	H-1→L (0.74), H→L+1 (0.23)
mPW2PLYP	$S_0 \rightarrow S_3$	423.2	2.930	1.78	H-1→L (0.74), H→L+1 (0.22)
B2PLYP	$S_0 \rightarrow S_3$	430.3	2.881	1.75	H-1→L (0.74), H→L+1 (0.22)
PBE-QIDH	$S_0 \rightarrow S_3$	413.5	2.999	1.85	H-1→L (0.74), H→L+1 (0.22)
B2GPPLYP	$S_0 \rightarrow S_3$	426.2	2.909	1.79	H-1→L (0.75), H→L+1 (0.22)
PBE0-2	$S_0 \rightarrow S_3$	423.2	2.930	1.82	H-1→L (0.75), H→L+1 (0.23)
RSX-0DH	$S_0 \rightarrow S_3$	378.6	3.275	2.05	H-1→L (0.71), H→L+1 (0.24)
ωB2PLYP	$S_0 \rightarrow S_3$	396.6	3.126	1.96	H-1→L (0.72), H→L+1 (0.24)
RSX-QIDH	$S_0 \rightarrow S_3$	394.6	3.142	1.97	H-1→L (0.73), H→L+1 (0.23)
ωB2GPPLYP	$S_0 \rightarrow S_3$	401.9	3.085	1.94	H-1→L (0.73), H→L+1 (0.23)
ωB88PP86	$S_0 \rightarrow S_3$	413.6	2.998	1.88	H-1→L (0.73), H→L+1 (0.23)
ωPBEPP86	$S_0 \rightarrow S_3$	418.3	2.964	1.85	H-1→L (0.73), H→L+1 (0.23)
DSD-BLYP	$S_0 \rightarrow S_3$	529.8	2.340	1.45	H-1→L (0.75), H→L+1 (0.21)
DSD-PBEP86	$S_0 \rightarrow S_3$	539.6	2.298	1.42	H-1→L (0.75), H→L+1 (0.21)
SCS/SOS-B2PLYP	$S_0 \rightarrow S_3$	389.5	3.183	1.93	H-1→L (0.74), H→L+1 (0.22)
SCS-B2GPPLYP	$S_0 \rightarrow S_3$	408.9	3.032	1.87	H-1→L (0.75), H→L+1 (0.22)
SOS-B2GPPLYP	$S_0 \rightarrow S_3$	415.7	2.982	1.84	H-1→L (0.75), H→L+1 (0.22)
SCS-PBE-QIDH	$S_0 \rightarrow S_3$	430.7	2.879	1.77	H-1→L (0.74), H→L+1 (0.22)
SOS-PBE-QIDH	$S_0 \rightarrow S_3$	432.2	2.869	1.77	H-1→L (0.74), H→L+1 (0.22)
SCS/SOS-ωB2PLYP	$S_0 \rightarrow S_3$	410.4	3.021	1.90	H-1→L (0.72), H→L+1 (0.24)
SCS-ωB2GPPLYP	$S_0 \rightarrow S_3$	431.1	2.876	1.81	H-1→L (0.73), H→L+1 (0.23)
SOS-ωB2GPPLYP	$S_0 \rightarrow S_3$	422.4	2.936	1.85	H-1→L (0.73), H→L+1 (0.23)
SCS-RSX-QIDH	$S_0 \rightarrow S_3$	453.9	2.731	1.72	H-1→L (0.73), H→L+1 (0.23)
SOS-RSX-QIDH	$S_0 \rightarrow S_3$	445.9	2.780	1.75	H-1→L (0.73), H→L+1 (0.23)
SCS-ωB88PP86	$S_0 \rightarrow S_3$	423.6	2.927	1.83	H-1→L (0.73), H→L+1 (0.23)
SOS-ωB88PP86	$S_0 \rightarrow S_3$	426.3	2.908	1.82	H-1→L (0.73), H→L+1 (0.23)
SCS-ωPBEPP86	$S_0 \rightarrow S_3$	451.8	2.744	1.72	H-1→L (0.73), H→L+1 (0.23)
SOS-ωPBEPP86	$S_0 \rightarrow S_3$	436.6	2.840	1.78	H-1→L (0.73), H→L+1 (0.23)

Table S16: Calculated Vertical Excitaion Energies (EE) in nm and eV, Oscillator Strength (f), and their MO Designation and Corresponding Coefficients, of the B Band of Molecule **13**, based on BPE0/def2-TZVP/CPCM optimized geometry.

Functional	Electronic transition	EE (nm)	EE (eV)	f	MO designation (coefficient)
PBE0-DH	$S_0 \rightarrow S_3$	393.5	3.151	1.63	H-1→L (0.83)
mPW2PLYP	$S_0 \rightarrow S_4$	410.4	3.021	1.58	H-1→L+1 (0.83)
B2PLYP	$S_0 \rightarrow S_4$	417.5	2.970	1.55	H-1→L+1 (0.83)
PBE-QIDH	$S_0 \rightarrow S_4$	401.4	3.089	1.65	H-1→L+1 (0.82)
B2GPPLYP	$S_0 \rightarrow S_4$	413.3	3.000	1.59	H-1→L+1 (0.83)
PBE0-2	$S_0 \rightarrow S_4$	410.6	3.020	1.64	H-1→L+1 (0.82)
RSX-0DH	$S_0 \rightarrow S_4$	367.3	3.375	1.84	H-1→L+1 (0.80)
ωB2PLYP	$S_0 \rightarrow S_4$	384.5	3.225	1.76	H-1→L+1 (0.80)
RSX-QIDH	$S_0 \rightarrow S_3$	383.2	3.236	1.77	H-1→L (0.80)
ωB2GPPLYP	$S_0 \rightarrow S_4$	389.8	3.181	1.74	H-1→L+1 (0.80)
ωB88PP86	$S_0 \rightarrow S_3$	401.6	3.087	1.68	H-1→L (0.81)
ωPBEPP86	$S_0 \rightarrow S_3$	406.2	3.052	1.66	H-1→L (0.81)
DSD-BLYP	$S_0 \rightarrow S_4$	513.2	2.416	1.30	H-1→L+1 (0.82)
DSD-PBEP86	$S_0 \rightarrow S_4$	523.7	2.367	1.27	H-1→L (0.82)
SCS/SOS-B2PLYP	$S_0 \rightarrow S_4$	377.6	3.284	1.71	H-1→L (0.83)
SCS-B2GPPLYP	$S_0 \rightarrow S_3$	397.1	3.122	1.66	H-1→L (0.83)
SOS-B2GPPLYP	$S_0 \rightarrow S_3$	403.9	3.070	1.63	H-1→L (0.83)
SCS-PBE-QIDH	$S_0 \rightarrow S_4$	419.2	2.958	1.58	H-1→L+1 (0.82)
SOS-PBE-QIDH	$S_0 \rightarrow S_4$	420.9	2.946	1.57	H-1→L+1 (0.82)
SCS/SOS-ωB2PLYP	$S_0 \rightarrow S_4$	398.9	3.108	1.70	H-1→L+1 (0.80)
SCS-ωB2GPPLYP	$S_0 \rightarrow S_4$	419.4	2.956	1.62	H-1→L+1 (0.80)
SOS-ωB2GPPLYP	$S_0 \rightarrow S_4$	410.9	3.018	1.63	H-1→L (0.83)
SCS-RSX-QIDH	$S_0 \rightarrow S_4$	442.1	2.804	1.54	H-1→L+1 (0.80)
SOS-RSX-QIDH	$S_0 \rightarrow S_3$	435.2	2.849	1.56	H-1→L (0.80)
SCS-ωB88PP86	$S_0 \rightarrow S_3$	412.3	3.007	1.64	H-1→L+1 (0.81)
SOS-ωB88PP86	$S_0 \rightarrow S_3$	415.2	2.986	1.62	H-1→L (0.81)
SCS-ωPBEPP86	$S_0 \rightarrow S_3$	440.2	2.817	1.53	H-1→L (0.81)
SOS-ωPBEPP86	$S_0 \rightarrow S_3$	425.5	2.914	1.59	H-1→L (0.81)

S4 Calculated Vertical Excitaion Energies of Q1 Band

Table S17: Calculated Vertical Excitaion Energies (EE) in nm and eV, Oscillator Strength (f), and their MO Designation and Corresponding Coefficients, of the Q1 Band of Molecule **1**, based on BPE0/def2-TZVP/CPCM optimized geometry.

Functional	Electronic transition	EE (nm)	EE (eV)	f	MO designation (coefficient)
PBE0-DH	$S_0 \to S_2$	500.6	2.477	0.009	H→L+1 (0.53), H-1→L (0.46)
mPW2PLYP	$S_0 \to S_2$	498.0	2.489	0.010	H→L+1 (0.55), H-1→L (0.43)
B2PLYP	$S_0 \to S_2$	496.7	2.496	0.011	H→L+1 (0.54), H-1→L (0.43)
PBE-QIDH	$S_0 \to S_2$	492.9	2.515	0.020	H→L+1 (0.57), H-1→L (0.40)
B2GPPLYP	$S_0 \to S_2$	490.2	2.529	0.019	H→L+1 (0.57), H-1→L (0.40)
PBE0-2	$S_0 \to S_2$	477.1	2.599	0.027	H→L+1 (0.59), H-1→L (0.38)
RSX-0DH	$S_0 \to S_2$	560.0	2.214	0.015	H→L+1 (0.56), H-1→L (0.41)
ωB2PLYP	$S_0 \to S_2$	536.8	2.310	0.020	H→L+1 (0.56), H-1→L (0.40)
RSX-QIDH	$S_0 \to S_2$	525.4	2.360	0.020	H→L+1 (0.58), H-1→L (0.39)
ωB2GPPLYP	$S_0 \to S_2$	521.7	2.376	0.020	H→L+1 (0.57), H-1→L (0.40)
ωB88PP86	$S_0 \to S_2$	502.1	2.469	0.019	H→L+1 (0.57), H-1→L (0.40)
ωPBEPP86	$S_0 \to S_2$	491.4	2.523	0.020	H→L+1 (0.57), H-1→L (0.40)
DSD-BLYP	$S_0 \to S_2$	413.6	2.997	0.030	H→L+1 (0.58), H-1→L (0.40)
DSD-PBEP86	$S_0 \to S_2$	417.5	2.969	0.030	H→L+1 (0.58), H-1→L (0.40)
SCS/SOS-B2PLYP	$S_0 \to S_2$	459.3	2.699	0.012	H→L+1 (0.54), H-1→L (0.43)
SCS-B2GPPLYP	$S_0 \to S_2$	505.3	2.454	0.018	H→L+1 (0.57), H-1→L (0.41)
SOS-B2GPPLYP	$S_0 \to S_2$	519.5	2.387	0.018	H→L+1 (0.57), H-1→L (0.41)
SCS-PBE-QIDH	$S_0 \to S_2$	544.9	2.275	0.018	H→L+1 (0.57), H-1→L (0.40)
SOS-PBE-QIDH	$S_0 \to S_2$	563.6	2.200	0.017	H→L+1 (0.57), H-1→L (0.40)
SCS/SOS-ωB2PLYP	$S_0 \to S_2$	618.0	2.006	0.014	H→L+1 (0.56), H-1→L (0.40)
SCS-ωB2GPPLYP	$S_0 \to S_2$	641.5	1.933	0.017	H→L+1 (0.57), H-1→L (0.39)
SOS-ωB2GPPLYP	$S_0 \to S_2$	637.5	1.945	0.017	H→L+1 (0.57), H-1→L (0.39)
SCS-RSX-QIDH	$S_0 \to S_2$	625.7	1.982	0.018	H→L+1 (0.58), H-1→L (0.39)
SOS-RSX-QIDH	$S_0 \to S_2$	735.3	1.686	0.015	H→L+1 (0.58), H-1→L (0.39)
SCS-ωB88PP86	$S_0 \to S_2$	590.4	2.100	0.016	H→L+1 (0.57), H-1→L (0.40)
SOS-ωB88PP86	$S_0 \to S_2$	618.3	2.005	0.015	H→L+1 (0.57), H-1→L (0.40)
SCS-ωPBEPP86	$S_0 \to S_2$	634.7	1.953	0.016	H→L+1 (0.57), H-1→L (0.40)
SOS-ωPBEPP86	$S_0 \to S_2$	645.8	1.920	0.016	H→L+1 (0.57), H-1→L (0.40)

Table S18: Calculated Vertical Excitaion Energies (EE) in nm and eV, Oscillator Strength (f), and their MO Designation and Corresponding Coefficients, of the Q1 Band of Molecule **2**, based on BPE0/def2-TZVP/CPCM optimized geometry.

Functional	Electronic transition	EE (nm)	EE (eV)	f	MO designation (coefficient)
PBE0-DH	$S_0 \rightarrow S_1$	520.6	2.382	0.011	H→L (0.49), H-1→L+1 (0.40)
mPW2PLYP	$S_0 \rightarrow S_1$	524.1	2.366	0.016	H→L (0.47), H-1→L+1 (0.36)
B2PLYP	$S_0 \rightarrow S_1$	523.8	2.367	0.015	H→L (0.54), H-1→L+1 (0.41)
PBE-QIDH	$S_0 \rightarrow S_1$	523.0	2.371	0.025	H→L (0.50), H-1→L+1 (0.34)
B2GPPLYP	$S_0 \rightarrow S_1$	521.5	2.377	0.023	H→L (0.54), H-1→L+1 (0.37)
PBE0-2	$S_0 \rightarrow S_1$	514.4	2.410	0.034	H→L (0.52), H-1→L+1 (0.33)
RSX-0DH	$S_0 \rightarrow S_1$	591.5	2.096	0.019	H→L (0.40), H-1→L+1 (0.28)
ωB2PLYP	$S_0 \rightarrow S_1$	573.1	2.164	0.020	H→L (0.34), H-1→L+1 (0.24)
RSX-QIDH	$S_0 \rightarrow S_1$	562.6	2.204	0.027	H→L (0.41), H-1→L+1 (0.26)
ωB2GPPLYP	$S_0 \rightarrow S_1$	560.1	2.214	0.026	H→L (0.34), H-1→L+1 (0.25)
ωB88PP86	$S_0 \rightarrow S_1$	539.3	2.299	0.020	H→L (0.35), H-1→L+1 (0.24)
ωPBEPP86	$S_0 \rightarrow S_1$	530.0	2.339	0.026	H→L (0.32), H-1→L+1 (0.26)
DSD-BLYP	$S_0 \rightarrow S_1$	464.2	2.671	0.035	H→L (0.54), H-1→L+1 (0.34)
DSD-PBEP86	$S_0 \rightarrow S_1$	467.7	2.651	0.030	H→L (0.47), H-1→L+1 (0.31)
SCS/SOS-B2PLYP	$S_0 \rightarrow S_1$	480.9	2.578	0.016	H→L (0.54), H-1→L+1 (0.41)
SCS-B2GPPLYP	$S_0 \rightarrow S_1$	542.8	2.284	0.022	H→L (0.54), H-1→L+1 (0.37)
SOS-B2GPPLYP	$S_0 \rightarrow S_1$	560.7	2.211	0.022	H→L (0.54), H-1→L+1 (0.37)
SCS-PBE-QIDH	$S_0 \rightarrow S_1$	595.1	2.083	0.022	H→L (0.50), H-1→L+1 (0.34)
SOS-PBE-QIDH	$S_0 \rightarrow S_1$	620.7	1.998	0.020	H→L (0.50), H-1→L+1 (0.34)
SCS/SOS-ωB2PLYP	$S_0 \rightarrow S_1$	685.4	1.809	0.017	H→L (0.34), H-1→L+1 (0.24)
SCS-ωB2GPPLYP	$S_0 \rightarrow S_1$	726.0	1.708	0.020	H→L (0.34), H-1→L+1 (0.25)
SOS-ωB2GPPLYP	$S_0 \rightarrow S_1$	722.8	1.715	0.020	H→L (0.34), H-1→L+1 (0.25)
SCS-RSX-QIDH	$S_0 \rightarrow S_1$	700.9	1.769	0.020	H→L (0.41), H-1→L+1 (0.26)
SOS-RSX-QIDH	$S_0 \rightarrow S_1$	873.0	1.420	0.017	H→L (0.41), H-1→L+1 (0.26)
SCS-ωB88PP86	$S_0 \rightarrow S_1$	653.9	1.896	0.019	H→L (0.35), H-1→L+1 (0.24)
SOS-ωB88PP86	$S_0 \rightarrow S_1$	692.4	1.791	0.018	H→L (0.35), H-1→L+1 (0.24)
SCS-ωPBEPP86	$S_0 \rightarrow S_1$	713.8	1.737	0.019	H→L (0.32), H-1→L+1 (0.26)
SOS-ωPBEPP86	$S_0 \rightarrow S_1$	734.4	1.688	0.019	H→L (0.32), H-1→L+1 (0.26)

Table S19: Calculated Vertical Excitaion Energies (EE) in nm and eV, Oscillator Strength (f), and their MO Designation and Corresponding Coefficients, of the Q1 Band of Molecule **3**, based on BPE0/def2-TZVP/CPCM optimized geometry.

Functional	Electronic transition	EE (nm)	EE (eV)	f	MO designation (coefficient)
PBE0-DH	$S_0 \rightarrow S_1$	510.0	2.431	0.018	H→L (0.57), H-1→L+1 (0.42)
mPW2PLYP	$S_0 \rightarrow S_1$	512.9	2.417	0.023	H→L (0.58), H-1→L+1 (0.40)
B2PLYP	$S_0 \rightarrow S_1$	512.5	2.419	0.021	H→L (0.58), H-1→L+1 (0.41)
PBE-QIDH	$S_0 \rightarrow S_1$	511.8	2.423	0.030	H→L (0.58), H-1→L+1 (0.36)
B2GPPLYP	$S_0 \rightarrow S_1$	510.2	2.430	0.030	H→L (0.59), H-1→L+1 (0.37)
PBE0-2	$S_0 \rightarrow S_1$	503.3	2.464	0.041	H→L (0.48), H-1→L+1 (0.28)
RSX-0DH	$S_0 \rightarrow S_1$	575.2	2.155	0.025	H→L (0.57), H-1→L+1 (0.37)
ωB2PLYP	$S_0 \rightarrow S_1$	557.5	2.224	0.027	H→L (0.57), H-1→L+1 (0.37)
RSX-QIDH	$S_0 \rightarrow S_1$	548.2	2.262	0.030	H→L (0.54), H-1→L+1 (0.33)
ωB2GPPLYP	$S_0 \rightarrow S_1$	545.6	2.273	0.032	H→L (0.56), H-1→L+1 (0.34)
ωB88PP86	$S_0 \rightarrow S_1$	525.9	2.358	0.030	H→L (0.56), H-1→L+1 (0.36)
ωPBEPP86	$S_0 \rightarrow S_1$	517.2	2.397	0.030	H→L (0.55), H-1→L+1 (0.34)
DSD-BLYP	$S_0 \rightarrow S_1$	454.3	2.729	0.043	H→L (0.53), H-1→L+1 (0.31)
DSD-PBEP86	$S_0 \rightarrow S_1$	457.5	2.710	0.039	H→L (0.57), H-1→L+1 (0.34)
SCS/SOS-B2PLYP	$S_0 \rightarrow S_1$	471.8	2.628	0.023	H→L (0.58), H-1→L+1 (0.41)
SCS-B2GPPLYP	$S_0 \rightarrow S_1$	530.9	2.335	0.029	H→L (0.59), H-1→L+1 (0.37)
SOS-B2GPPLYP	$S_0 \rightarrow S_1$	548.0	2.262	0.028	H→L (0.59), H-1→L+1 (0.37)
SCS-PBE-QIDH	$S_0 \rightarrow S_1$	580.7	2.135	0.028	H→L (0.58), H-1→L+1 (0.36)
SOS-PBE-QIDH	$S_0 \rightarrow S_1$	604.9	2.050	0.027	H→L (0.58), H-1→L+1 (0.36)
SCS/SOS-ωB2PLYP	$S_0 \rightarrow S_1$	662.6	1.871	0.020	H→L (0.57), H-1→L+1 (0.37)
SCS-ωB2GPPLYP	$S_0 \rightarrow S_1$	701.5	1.767	0.025	H→L (0.56), H-1→L+1 (0.34)
SOS-ωB2GPPLYP	$S_0 \rightarrow S_1$	697.6	1.777	0.025	H→L (0.56), H-1→L+1 (0.34)
SCS-RSX-QIDH	$S_0 \rightarrow S_1$	679.7	1.824	0.030	H→L (0.54), H-1→L+1 (0.33)
SOS-RSX-QIDH	$S_0 \rightarrow S_1$	835.0	1.485	0.020	H→L (0.54), H-1→L+1 (0.33)
SCS-ωB88PP86	$S_0 \rightarrow S_1$	634.7	1.953	0.020	H→L (0.56), H-1→L+1 (0.36)
SOS-ωB88PP86	$S_0 \rightarrow S_1$	670.0	1.850	0.023	H→L (0.56), H-1→L+1 (0.36)
SCS-ωPBEPP86	$S_0 \rightarrow S_1$	692.2	1.791	0.024	H→L (0.55), H-1→L+1 (0.34)
SOS-ωPBEPP86	$S_0 \rightarrow S_1$	709.3	1.748	0.024	H→L (0.55), H-1→L+1 (0.34)

Table S20: Calculated Vertical Excitaion Energies (EE) in nm and eV, Oscillator Strength (f), and their MO Designation and Corresponding Coefficients, of the Q1 Band of Molecule **4**, based on BPE0/def2-TZVP/CPCM optimized geometry.

Functional	Electronic transition	EE (nm)	EE (eV)	f	MO designation (coefficient)
PBE0-DH	$S_0 \rightarrow S_2$	509.8	2.432	0.040	H→L+1 (0.63), H-1→L (0.36)
mPW2PLYP	$S_0 \rightarrow S_2$	505.9	2.451	0.050	H→L+1 (0.64), H-1→L (0.35)
B2PLYP	$S_0 \rightarrow S_2$	503.9	2.460	0.048	H→L+1 (0.63), H-1→L (0.35)
PBE-QIDH	$S_0 \rightarrow S_2$	499.1	2.484	0.060	H→L+1 (0.65), H-1→L (0.33)
B2GPPLYP	$S_0 \rightarrow S_2$	496.0	2.499	0.060	H→L+1 (0.65), H-1→L (0.33)
PBE0-2	$S_0 \rightarrow S_2$	479.8	2.584	0.070	H→L+1 (0.66), H-1→L (0.31)
RSX-0DH	$S_0 \rightarrow S_2$	571.2	2.170	0.047	H→L+1 (0.63), H-1→L (0.33)
ωB2PLYP	$S_0 \rightarrow S_2$	544.8	2.276	0.050	H→L+1 (0.64), H-1→L (0.33)
RSX-QIDH	$S_0 \rightarrow S_2$	531.4	2.333	0.059	H→L+1 (0.64), H-1→L (0.32)
ωB2GPPLYP	$S_0 \rightarrow S_2$	527.4	2.351	0.058	H→L+1 (0.64), H-1→L (0.32)
ωB88PP86	$S_0 \rightarrow S_2$	506.9	2.446	0.057	H→L+1 (0.64), H-1→L (0.33)
ωPBEPP86	$S_0 \rightarrow S_2$	495.0	2.505	0.060	H→L+1 (0.65), H-1→L (0.32)
DSD-BLYP	$S_0 \rightarrow S_2$	410.1	3.023	0.082	H→L+1 (0.66), H-1→L (0.32)
DSD-PBEP86	$S_0 \rightarrow S_2$	414.5	2.991	0.078	H→L+1 (0.65), H-1→L (0.32)
SCS/SOS-B2PLYP	$S_0 \rightarrow S_2$	464.0	2.672	0.052	H→L+1 (0.63), H-1→L (0.35)
SCS-B2GPPLYP	$S_0 \rightarrow S_2$	509.8	2.432	0.060	H→L+1 (0.65), H-1→L (0.33)
SOS-B2GPPLYP	$S_0 \rightarrow S_2$	524.8	2.363	0.060	H→L+1 (0.65), H-1→L (0.33)
SCS-PBE-QIDH	$S_0 \rightarrow S_2$	551.7	2.247	0.060	H→L+1 (0.65), H-1→L (0.33)
SOS-PBE-QIDH	$S_0 \rightarrow S_2$	569.8	2.176	0.054	H→L+1 (0.65), H-1→L (0.33)
SCS/SOS-ωB2PLYP	$S_0 \rightarrow S_2$	628.4	1.973	0.040	H→L+1 (0.64), H-1→L (0.33)
SCS-ωB2GPPLYP	$S_0 \rightarrow S_2$	656.2	1.889	0.046	H→L+1 (0.64), H-1→L (0.32)
SOS-ωB2GPPLYP	$S_0 \rightarrow S_2$	646.2	1.919	0.047	H→L+1 (0.64), H-1→L (0.32)
SCS-RSX-QIDH	$S_0 \rightarrow S_2$	650.6	1.906	0.048	H→L+1 (0.64), H-1→L (0.32)
SOS-RSX-QIDH	$S_0 \rightarrow S_2$	747.2	1.659	0.040	H→L+1 (0.64), H-1→L (0.32)
SCS-ωB88PP86	$S_0 \rightarrow S_2$	601.0	2.063	0.048	H→L+1 (0.64), H-1→L (0.33)
SOS-ωB88PP86	$S_0 \rightarrow S_2$	627.0	1.977	0.046	H→L+1 (0.64), H-1→L (0.33)
SCS-ωPBEPP86	$S_0 \rightarrow S_2$	656.0	1.890	0.046	H→L+1 (0.65), H-1→L (0.32)
SOS-ωPBEPP86	$S_0 \rightarrow S_2$	655.1	1.893	0.046	H→L+1 (0.65), H-1→L (0.32)

Table S21: Calculated Vertical Excitaion Energies (EE) in nm and eV, Oscillator Strength (f), and their MO Designation and Corresponding Coefficients, of the Q1 Band of Molecule **5**, based on BPE0/def2-TZVP/CPCM optimized geometry.

Functional	Electronic transition	EE (nm)	EE (eV)	f	MO designation (coefficient)
PBE0-DH	$S_0 \rightarrow S_1$	530.2	2.338	0.051	H→L+1 (0.63), H-1→L (0.35)
mPW2PLYP	$S_0 \rightarrow S_1$	532.1	2.330	0.058	H→L+1 (0.65), H-1→L (0.33)
B2PLYP	$S_0 \rightarrow S_1$	531.0	2.335	0.056	H→L+1 (0.65), H-1→L (0.34)
PBE-QIDH	$S_0 \rightarrow S_1$	528.9	2.344	0.070	H→L+1 (0.66), H-1→L (0.31)
B2GPPLYP	$S_0 \rightarrow S_1$	527.1	2.352	0.069	H→L+1 (0.66), H-1→L (0.31)
PBE0-2	$S_0 \rightarrow S_1$	516.2	2.402	0.084	H→L+1 (0.67), H-1→L (0.29)
RSX-0DH	$S_0 \rightarrow S_1$	603.6	2.054	0.054	H→L+1 (0.64), H-1→L (0.32)
ωB2PLYP	$S_0 \rightarrow S_1$	581.5	2.132	0.058	H→L+1 (0.65), H-1→L (0.32)
RSX-QIDH	$S_0 \rightarrow S_1$	568.4	2.181	0.068	H→L+1 (0.66), H-1→L (0.31)
ωB2GPPLYP	$S_0 \rightarrow S_1$	565.7	2.192	0.067	H→L+1 (0.66), H-1→L (0.31)
ωB88PP86	$S_0 \rightarrow S_1$	543.6	2.281	0.066	H→L+1 (0.65), H-1→L (0.31)
ωPBEPP86	$S_0 \rightarrow S_1$	532.8	2.327	0.071	H→L+1 (0.66), H-1→L (0.31)
DSD-BLYP	$S_0 \rightarrow S_1$	457.3	2.711	0.091	H→L+1 (0.67), H-1→L (0.30)
DSD-PBEP86	$S_0 \rightarrow S_1$	461.3	2.688	0.086	H→L+1 (0.67), H-1→L (0.30)
SCS/SOS-B2PLYP	$S_0 \rightarrow S_1$	485.6	2.553	0.060	H→L+1 (0.64), H-1→L (0.33)
SCS-B2GPPLYP	$S_0 \rightarrow S_1$	546.8	2.267	0.070	H→L+1 (0.66), H-1→L (0.31)
SOS-B2GPPLYP	$S_0 \rightarrow S_1$	565.6	2.192	0.064	H→L+1 (0.66), H-1→L (0.31)
SCS-PBE-QIDH	$S_0 \rightarrow S_1$	601.3	2.062	0.060	H→L+1 (0.66), H-1→L (0.31)
SOS-PBE-QIDH	$S_0 \rightarrow S_1$	626.1	1.980	0.060	H→L+1 (0.66), H-1→L (0.31)
SCS/SOS-ωB2PLYP	$S_0 \rightarrow S_1$	696.8	1.779	0.048	H→L+1 (0.65), H-1→L (0.32)
SCS-ωB2GPPLYP	$S_0 \rightarrow S_1$	742.8	1.669	0.050	H→L+1 (0.66), H-1→L (0.31)
SOS-ωB2GPPLYP	$S_0 \rightarrow S_1$	731.7	1.694	0.050	H→L+1 (0.66), H-1→L (0.31)
SCS-RSX-QIDH	$S_0 \rightarrow S_1$	730.2	1.698	0.050	H→L+1 (0.66), H-1→L (0.31)
SOS-RSX-QIDH	$S_0 \rightarrow S_1$	885.1	1.401	0.044	H→L+1 (0.66), H-1→L (0.31)
SCS-ωB88PP86	$S_0 \rightarrow S_1$	665.1	1.864	0.053	H→L+1 (0.65), H-1→L (0.31)
SOS-ωB88PP86	$S_0 \rightarrow S_1$	701.0	1.769	0.051	H→L+1 (0.65), H-1→L (0.31)
SCS-ωPBEPP86	$S_0 \rightarrow S_1$	738.3	1.679	0.051	H→L+1 (0.66), H-1→L (0.31)
SOS-ωPBEPP86	$S_0 \rightarrow S_1$	743.2	1.668	0.051	H→L+1 (0.66), H-1→L (0.31)

Table S22: Calculated Vertical Excitaion Energies (EE) in nm and eV, Oscillator Strength (f), and their MO Designation and Corresponding Coefficients, of the Q1 Band of Molecule **6**, based on BPE0/def2-TZVP/CPCM optimized geometry.

Functional	Electronic transition	EE (nm)	EE (eV)	f	MO designation (coefficient)
PBE0-DH	$S_0 \rightarrow S_1$	520.3	2.383	0.060	H→L+1 (0.66), H-1→L (0.32)
mPW2PLYP	$S_0 \rightarrow S_1$	521.7	2.376	0.070	H→L+1 (0.67), H-1→L (0.31)
B2PLYP	$S_0 \rightarrow S_1$	520.6	2.381	0.068	H→L+1 (0.66), H-1→L (0.31)
PBE-QIDH	$S_0 \rightarrow S_1$	518.5	2.391	0.082	H→L+1 (0.68), H-1→L (0.29)
B2GPPLYP	$S_0 \rightarrow S_1$	516.6	2.400	0.080	H→L+1 (0.66), H-1→L (0.29)
PBE0-2	$S_0 \rightarrow S_1$	506.0	2.450	0.100	H→L+1 (0.69), H-1→L (0.28)
RSX-0DH	$S_0 \rightarrow S_1$	587.7	2.109	0.060	H→L+1 (0.66), H-1→L (0.31)
ωB2PLYP	$S_0 \rightarrow S_1$	566.6	2.188	0.068	H→L+1 (0.65), H-1→L (0.30)
RSX-QIDH	$S_0 \rightarrow S_1$	554.8	2.235	0.078	H→L+1 (0.67), H-1→L (0.29)
ωB2GPPLYP	$S_0 \rightarrow S_1$	552.0	2.246	0.077	H→L+1 (0.66), H-1→L (0.29)
ωB88PP86	$S_0 \rightarrow S_1$	531.1	2.335	0.080	H→L+1 (0.62), H-1→L (0.28)
ωPBEPP86	$S_0 \rightarrow S_1$	521.0	2.380	0.080	H→L+1 (0.64), H-1→L (0.28)
DSD-BLYP	$S_0 \rightarrow S_1$	448.7	2.763	0.100	H→L+1 (0.68), H-1→L (0.28)
DSD-PBEP86	$S_0 \rightarrow S_1$	452.4	2.741	0.099	H→L+1 (0.68), H-1→L (0.29)
SCS/SOS-B2PLYP	$S_0 \rightarrow S_1$	477.2	2.598	0.070	H→L+1 (0.66), H-1→L (0.31)
SCS-B2GPPLYP	$S_0 \rightarrow S_1$	535.6	2.315	0.080	H→L+1 (0.67), H-1→L (0.29)
SOS-B2GPPLYP	$S_0 \rightarrow S_1$	553.6	2.239	0.080	H→L+1 (0.67), H-1→L (0.29)
SCS-PBE-QIDH	$S_0 \rightarrow S_1$	587.6	2.110	0.070	H→L+1 (0.68), H-1→L (0.29)
SOS-PBE-QIDH	$S_0 \rightarrow S_1$	611.0	2.029	0.070	H→L+1 (0.68), H-1→L (0.29)
SCS/SOS-ωB2PLYP	$S_0 \rightarrow S_1$	674.4	1.839	0.060	H→L+1 (0.65), H-1→L (0.30)
SCS-ωB2GPPLYP	$S_0 \rightarrow S_1$	718.3	1.726	0.060	H→L+1 (0.06), H-1→L (0.29)
SOS-ωB2GPPLYP	$S_0 \rightarrow S_1$	707.1	1.753	0.060	H→L+1 (0.66), H-1→L (0.29)
SCS-RSX-QIDH	$S_0 \rightarrow S_1$	708.5	1.750	0.060	H→L+1 (0.67), H-1→L (0.29)
SOS-RSX-QIDH	$S_0 \rightarrow S_1$	847.4	1.463	0.050	H→L+1 (0.67), H-1→L (0.29)
SCS-ωB88PP86	$S_0 \rightarrow S_1$	646.4	1.918	0.060	H→L+1 (0.66), H-1→L (0.29)
SOS-ωB88PP86	$S_0 \rightarrow S_1$	679.3	1.825	0.060	H→L+1 (0.62), H-1→L (0.28)
SCS-ωPBEPP86	$S_0 \rightarrow S_1$	716.5	1.730	0.060	H→L+1 (0.67), H-1→L (0.29)
SOS-ωPBEPP86	$S_0 \rightarrow S_1$	718.8	1.725	0.060	H→L+1 (0.64), H-1→L (0.28)

Table S23: Calculated Vertical Excitaion Energies (EE) in nm and eV, Oscillator Strength (f), and their MO Designation and Corresponding Coefficients, of the Q1 Band of Molecule **7**, based on BPE0/def2-TZVP/CPCM optimized geometry.

Functional	Electronic transition	EE (nm)	EE (eV)	f	MO designation (coefficient)
PBE0-DH	$S_0 \rightarrow S_2$	524.3	2.365	0.00950	H→L+1 (0.54), H-1→L (0.45)
mPW2PLYP	$S_0 \rightarrow S_2$	520.3	2.383	0.00500	H→L+1 (0.52), H-1→L (0.47)
B2PLYP	$S_0 \rightarrow S_2$	519.3	2.387	0.00630	H→L+1 (0.52), H-1→L (0.46)
PBE-QIDH	$S_0 \rightarrow S_2$	510.1	2.122	0.00090	H→L+1 (0.50), H-1→L (0.47)
B2GPPLYP	$S_0 \rightarrow S_2$	508.0	2.441	0.00140	H→L+1 (0.49), H-1→L (0.48)
PBE0-2	$S_0 \rightarrow S_2$	487.8	2.542	0.00070	H→L+1 (0.52), H-1→L (0.44)
RSX-0DH	$S_0 \rightarrow S_2$	582.1	2.130	0.00100	H→L+1 (0.44), H-1→L (0.42)
ωB2PLYP	$S_0 \rightarrow S_2$	555.2	2.233	0.00130	H→L+1 (0.50), H-1→L (0.46)
RSX-QIDH	$S_0 \rightarrow S_2$	540.2	2.295	0.00016	H→L+1 (0.49), H-1→L (0.43)
ωB2GPPLYP	$S_0 \rightarrow S_2$	536.3	2.312	0.00030	H→L+1 (0.51), H-1→L (0.45)
ωB88PP86	$S_0 \rightarrow S_2$	517.2	2.397	0.00100	H→L+1 (0.50), H-1→L (0.46)
ωPBEPP86	$S_0 \rightarrow S_2$	504.6	2.457	0.00047	H→L+1 (0.50), H-1→L (0.46)
DSD-BLYP	$S_0 \rightarrow S_2$	418.5	2.963	0.00008	H→L+1 (0.52), H-1→L (0.45)
DSD-PBEP86	$S_0 \rightarrow S_2$	425.0	2.917	0.00050	H→L+1 (0.51), H-1→L (0.46)
SCS/SOS-B2PLYP	$S_0 \rightarrow S_2$	473.5	2.618	0.00600	H→L+1 (0.52), H-1→L (0.46)
SCS-B2GPPLYP	$S_0 \rightarrow S_2$	520.8	2.381	0.00100	H→L+1 (0.49), H-1→L (0.48)
SOS-B2GPPLYP	$S_0 \rightarrow S_2$	537.2	2.308	0.00100	H→L+1 (0.49), H-1→L (0.48)
SCS-PBE-QIDH	$S_0 \rightarrow S_2$	566.9	2.187	0.00080	H→L+1 (0.50), H-1→L (0.47)
SOS-PBE-QIDH	$S_0 \rightarrow S_2$	586.2	2.115	0.00080	H→L+1 (0.50), H-1→L (0.47)
SCS/SOS-ωB2PLYP	$S_0 \rightarrow S_2$	644.8	1.923	0.00110	H→L+1 (0.50), H-1→L (0.46)
SCS-ωB2GPPLYP	$S_0 \rightarrow S_2$	676.8	1.832	0.00030	H→L+1 (0.51), H-1→L (0.45)
SOS-ωB2GPPLYP	$S_0 \rightarrow S_2$	664.1	1.867	0.00025	H→L+1 (0.51), H-1→L (0.45)
SCS-RSX-QIDH	$S_0 \rightarrow S_2$	675.3	1.836	0.00010	H→L+1 (0.49), H-1→L (0.43)
SOS-RSX-QIDH	$S_0 \rightarrow S_2$	776.6	1.596	0.00010	H→L+1 (0.49), H-1→L (0.43)
SCS-ωB88PP86	$S_0 \rightarrow S_2$	618.3	2.005	0.00083	H→L+1 (0.50), H-1→L (0.46)
SOS-ωB88PP86	$S_0 \rightarrow S_2$	645.9	1.920	0.00080	H→L+1 (0.50), H-1→L (0.46)
SCS-ωPBEPP86	$S_0 \rightarrow S_2$	681.6	1.819	0.00040	H→L+1 (0.50), H-1→L (0.46)
SOS-ωPBEPP86	$S_0 \rightarrow S_2$	676.8	1.315	0.00040	H→L+1 (0.50), H-1→L (0.46)

Table S24: Calculated Vertical Excitaion Energies (EE) in nm and eV, Oscillator Strength (f), and their MO Designation and Corresponding Coefficients, of the Q1 Band of Molecule **8**, based on BPE0/def2-TZVP/CPCM optimized geometry.

Functional	Electronic transition	EE (nm)	EE (eV)	f	MO designation (coefficient)
PBE0-DH	$S_0 \rightarrow S_1$	545.3	2.274	0.00400	H→L+1 (0.47), H-1→L (0.40)
mPW2PLYP	$S_0 \rightarrow S_1$	548.2	2.262	0.00160	H→L+1 (0.32), H-1→L (0.30)
B2PLYP	$S_0 \rightarrow S_1$	548.5	2.261	0.00220	H→L+1 (0.38), H-1→L (0.35)
PBE-QIDH	$S_0 \rightarrow S_1$	542.1	2.287	0.00002	H→L+1 (0.41), H-1→L (0.37)
B2GPPLYP	$S_0 \rightarrow S_1$	542.0	2.289	0.00002	H→L+1 (0.42), H-1→L (0.40)
PBE0-2	$S_0 \rightarrow S_1$	527.5	2.351	0.00100	H→L+1 (0.46), H-1→L (0.37)
RSX-0DH	$S_0 \rightarrow S_1$	614.8	2.017	0.00001	H→L+1 (0.47), H-1→L (0.44)
ωB2PLYP	$S_0 \rightarrow S_1$	593.5	2.089	0.00003	H→L+1 (0.39), H-1→L (0.35)
RSX-QIDH	$S_0 \rightarrow S_1$	579.2	2.141	0.00056	H→L+1 (0.43), H-1→L (0.35)
ωB2GPPLYP	$S_0 \rightarrow S_1$	576.9	2.149	0.00030	H→L+1 (0.41), H-1→L (0.35)
ωB88PP86	$S_0 \rightarrow S_1$	556.7	2.227	0.00001	H→L+1 (0.39), H-1→L (0.35)
ωPBEPP86	$S_0 \rightarrow S_1$	545.5	2.273	0.00020	H→L+1 (0.41), H-1→L (0.36)
DSD-BLYP	$S_0 \rightarrow S_1$	472.6	2.624	0.00090	H→L+1 (0.45), H-1→L (0.37)
DSD-PBEP86	$S_0 \rightarrow S_1$	478.8	2.589	0.00030	H→L+1 (0.43), H-1→L (0.37)
SCS/SOS-B2PLYP	$S_0 \rightarrow S_1$	495.9	2.500	0.00200	H→L+1 (0.38), H-1→L (0.34)
SCS-B2GPPLYP	$S_0 \rightarrow S_1$	560.6	2.212	0.00002	H→L+1 (0.42), H-1→L (0.40)
SOS-B2GPPLYP	$S_0 \rightarrow S_1$	581.4	2.132	0.00001	H→L+1 (0.42), H-1→L (0.40)
SCS-PBE-QIDH	$S_0 \rightarrow S_1$	621.6	1.995	0.00001	H→L+1 (0.41), H-1→L (0.37)
SOS-PBE-QIDH	$S_0 \rightarrow S_1$	648.4	1.912	0.00001	H→L+1 (0.41), H-1→L (0.37)
SCS/SOS-ωB2PLYP	$S_0 \rightarrow S_1$	718.6	1.725	0.00003	H→L+1 (0.39), H-1→L (0.35)
SCS-ωB2GPPLYP	$S_0 \rightarrow S_1$	773.4	1.603	0.00020	H→L+1 (0.42), H-1→L (0.35)
SOS-ωB2GPPLYP	$S_0 \rightarrow S_1$	758.6	1.634	0.00020	H→L+1 (0.42), H-1→L (0.35)
SCS-RSX-QIDH	$S_0 \rightarrow S_1$	766.1	1.618	0.00040	H→L+1 (0.43), H-1→L (0.35)
SOS-RSX-QIDH	$S_0 \rightarrow S_1$	934.6	1.327	0.00030	H→L+1 (0.43), H-1→L (0.35)
SCS-ωB88PP86	$S_0 \rightarrow S_1$	688.9	1.800	0.00001	H→L+1 (0.39), H-1→L (0.35)
SOS-ωB88PP86	$S_0 \rightarrow S_1$	727.6	1.704	0.00001	H→L+1 (0.39), H-1→L (0.35)
SCS-ωPBEPP86	$S_0 \rightarrow S_1$	775.6	1.599	0.00010	H→L+1 (0.41), H-1→L (0.36)
SOS-ωPBEPP86	$S_0 \rightarrow S_1$	775.7	1.598	0.00014	H→L+1 (0.41), H-1→L (0.36)

Table S25: Calculated Vertical Excitaion Energies (EE) in nm and eV, Oscillator Strength (f), and their MO Designation and Corresponding Coefficients, of the Q1 Band of Molecule **9**, based on BPE0/def2-TZVP/CPCM optimized geometry.

Functional	Electronic transition	EE (nm)	EE (eV)	f	MO designation (coefficient)
PBE0-DH	$S_0 \rightarrow S_1$	533.0	2.326	0.0010	H→L+1 (0.43), H-1→L (0.40)
mPW2PLYP	$S_0 \rightarrow S_1$	535.4	2.316	0.0001	H→L+1 (0.40), H-1→L (0.40)
B2PLYP	$S_0 \rightarrow S_1$	535.6	2.315	0.0003	H→L+1 (0.39), H-1→L (0.39)
PBE-QIDH	$S_0 \rightarrow S_1$	529.6	2.341	0.0009	H→L+1 (0.47), H-1→L (0.40)
B2GPPLYP	$S_0 \rightarrow S_1$	529.0	2.344	0.0005	H→L+1 (0.44), H-1→L (0.38)
PBE0-2	$S_0 \rightarrow S_1$	515.6	2.405	0.0040	H→L+1 (0.51), H-1→L (0.38)
RSX-0DH	$S_0 \rightarrow S_1$	596.6	2.078	0.0003	H→L+1 (0.49), H-1→L (0.41)
ωB2PLYP	$S_0 \rightarrow S_1$	576.2	2.151	0.0005	H→L+1 (0.48), H-1→L (0.40)
RSX-QIDH	$S_0 \rightarrow S_1$	563.6	2.200	0.0020	H→L+1 (0.51), H-1→L (0.40)
ωB2GPPLYP	$S_0 \rightarrow S_1$	561.2	2.209	0.0016	H→L+1 (0.49), H-1→L (0.39)
ωB88PP86	$S_0 \rightarrow S_1$	542.1	2.287	0.0008	H→L+1 (0.48), H-1→L (0.40)
ωPBEPP86	$S_0 \rightarrow S_1$	531.8	2.332	0.0010	H→L+1 (0.49), H-1→L (0.40)
DSD-BLYP	$S_0 \rightarrow S_1$	462.4	2.681	0.0030	H→L+1 (0.49), H-1→L (0.38)
DSD-PBEP86	$S_0 \rightarrow S_1$	468.2	2.648	0.0020	H→L+1 (0.48), H-1→L (0.38)
SCS/SOS-B2PLYP	$S_0 \rightarrow S_1$	485.7	2.553	0.0003	H→L+1 (0.39), H-1→L (0.39)
SCS-B2GPPLYP	$S_0 \rightarrow S_1$	547.5	2.265	0.0004	H→L+1 (0.44), H-1→L (0.38)
SOS-B2GPPLYP	$S_0 \rightarrow S_1$	567.3	2.185	0.0004	H→L+1 (0.44), H-1→L (0.38)
SCS-PBE-QIDH	$S_0 \rightarrow S_1$	605.3	2.048	0.0008	H→L+1 (0.47), H-1→L (0.40)
SOS-PBE-QIDH	$S_0 \rightarrow S_1$	630.6	1.966	0.0008	H→L+1 (0.47), H-1→L (0.40)
SCS/SOS-ωB2PLYP	$S_0 \rightarrow S_1$	692.7	1.790	0.0004	H→L+1 (0.48), H-1→L (0.40)
SCS-ωB2GPPLYP	$S_0 \rightarrow S_1$	744.6	1.665	0.0010	H→L+1 (0.49), H-1→L (0.39)
SOS-ωB2GPPLYP	$S_0 \rightarrow S_1$	730.2	1.698	0.0010	H→L+1 (0.49), H-1→L (0.39)
SCS-RSX-QIDH	$S_0 \rightarrow S_1$	739.6	1.676	0.0020	H→L+1 (0.51), H-1→L (0.40)
SOS-RSX-QIDH	$S_0 \rightarrow S_1$	889.9	1.393	0.0010	H→L+1 (0.51), H-1→L (0.40)
SCS-ωB88PP86	$S_0 \rightarrow S_1$	667.0	1.859	0.0006	H→L+1 (0.48), H-1→L (0.40)
SOS-ωB88PP86	$S_0 \rightarrow S_1$	702.3	1.765	0.0006	H→L+1 (0.48), H-1→L (0.40)
SCS-ωPBEPP86	$S_0 \rightarrow S_1$	749.0	1.655	0.0010	H→L+1 (0.49), H-1→L (0.40)
SOS-ωPBEPP86	$S_0 \rightarrow S_1$	747.1	1.660	0.0010	H→L+1 (0.49), H-1→L (0.40)

Table S26: Calculated Vertical Excitaion Energies (EE) in nm and eV, Oscillator Strength (f), and their MO Designation and Corresponding Coefficients, of the Q1 Band of Molecule **10**, based on BPE0/def2-TZVP/CPCM optimized geometry.

Functional	Electronic transition	EE (nm)	EE (eV)	f	MO designation (coefficient)
PBE0-DH	$S_0 \rightarrow S_2$	519.3	2.388	0.00025	H→L (0.50), H-1→L+1 (0.48)
mPW2PLYP	$S_0 \rightarrow S_2$	515.0	2.407	0.00010	H→L (0.50), H-1→L+1 (0.45)
B2PLYP	$S_0 \rightarrow S_2$	513.9	2.413	0.00005	H→L (0.51), H-1→L+1 (0.47)
PBE-QIDH	$S_0 \rightarrow S_2$	505.8	2.451	0.00200	H→L (0.54), H-1→L+1 (0.43)
B2GPPLYP	$S_0 \rightarrow S_2$	503.3	2.463	0.00100	H→L (0.54), H-1→L+1 (0.44)
PBE0-2	$S_0 \rightarrow S_2$	483.7	2.563	0.00500	H→L (0.56), H-1→L+1 (0.40)
RSX-0DH	$S_0 \rightarrow S_2$	579.1	2.141	0.00084	H→L (0.53), H-1→L+1 (0.42)
ωB2PLYP	$S_0 \rightarrow S_2$	551.9	2.246	0.00090	H→L (0.54), H-1→L+1 (0.43)
RSX-QIDH	$S_0 \rightarrow S_2$	536.7	2.310	0.00290	H→L (0.55), H-1→L+1 (0.41)
ωB2GPPLYP	$S_0 \rightarrow S_2$	532.9	2.327	0.00230	H→L (0.55), H-1→L+1 (0.41)
ωB88PP86	$S_0 \rightarrow S_2$	513.1	2.416	0.00200	H→L (0.54), H-1→L+1 (0.42)
ωPBEPP86	$S_0 \rightarrow S_2$	500.5	2.477	0.00200	H→L (0.55), H-1→L+1 (0.42)
DSD-BLYP	$S_0 \rightarrow S_2$	413.0	2.999	0.00500	H→L (0.56), H-1→L+1 (0.41)
DSD-PBEP86	$S_0 \rightarrow S_2$	419.5	2.956	0.00300	H→L (0.55), H-1→L+1 (0.42)
SCS/SOS-B2PLYP	$S_0 \rightarrow S_2$	469.8	2.639	0.00005	H→L (0.51), H-1→L+1 (0.47)
SCS-B2GPPLYP	$S_0 \rightarrow S_2$	517.3	2.397	0.00100	H→L (0.54), H-1→L+1 (0.44)
SOS-B2GPPLYP	$S_0 \rightarrow S_2$	533.7	2.323	0.00100	H→L (0.54), H-1→L+1 (0.44)
SCS-PBE-QIDH	$S_0 \rightarrow S_2$	562.9	2.203	0.00200	H→L (0.54), H-1→L+1 (0.43)
SOS-PBE-QIDH	$S_0 \rightarrow S_2$	582.3	2.129	0.00200	H→L (0.54), H-1→L+1 (0.43)
SCS/SOS-ωB2PLYP	$S_0 \rightarrow S_2$	642.2	1.930	0.00080	H→L (0.54), H-1→L+1 (0.43)
SCS-ωB2GPPLYP	$S_0 \rightarrow S_2$	674.3	1.839	0.00200	H→L (0.55), H-1→L+1 (0.41)
SOS-ωB2GPPLYP	$S_0 \rightarrow S_2$	661.4	1.875	0.00200	H→L (0.55), H-1→L+1 (0.41)
SCS-RSX-QIDH	$S_0 \rightarrow S_2$	672.1	1.845	0.00200	H→L (0.55), H-1→L+1 (0.41)
SOS-RSX-QIDH	$S_0 \rightarrow S_2$	773.0	1.604	0.00200	H→L (0.55), H-1→L+1 (0.41)
SCS-ωB88PP86	$S_0 \rightarrow S_2$	615.1	2.016	0.00100	H→L (0.54), H-1→L+1 (0.42)
SOS-ωB88PP86	$S_0 \rightarrow S_2$	642.4	1.930	0.00100	H→L (0.54), H-1→L+1 (0.42)
SCS-ωPBEPP86	$S_0 \rightarrow S_2$	678.3	1.828	0.00300	H→L (0.55), H-1→L+1 (0.42)
SOS-ωPBEPP86	$S_0 \rightarrow S_2$	673.1	1.842	0.00200	H→L (0.55), H-1→L+1 (0.42)

Table S27: Calculated Vertical Excitaion Energies (EE) in nm and eV, Oscillator Strength (f), and their MO Designation and Corresponding Coefficients, of the Q1 Band of Molecule **11**, based on BPE0/def2-TZVP/CPCM optimized geometry.

Functional	Electronic transition	EE (nm)	EE (eV)	f	MO designation (coefficient)
PBE0-DH	$S_0 \rightarrow S_2$	567.4	2.185	0.100	H→L+1 (0.69), H-1→L (0.29)
mPW2PLYP	$S_0 \rightarrow S_2$	571.2	2.170	0.100	H→L+1 (0.67), H-1→L (0.31)
B2PLYP	$S_0 \rightarrow S_2$	573.0	2.164	0.100	H→L+1 (0.67), H-1→L (0.31)
PBE-QIDH	$S_0 \rightarrow S_2$	562.0	2.206	0.070	H→L+1 (0.62), H-1→L (0.35)
B2GPPLYP	$S_0 \rightarrow S_2$	564.9	2.195	0.077	H→L+1 (0.63), H-1→L (0.34)
PBE0-2	$S_0 \rightarrow S_2$	559.6	2.215	0.060	H→L+1 (0.58), H-1→L (0.38)
RSX-0DH	$S_0 \rightarrow S_2$	587.2	2.111	0.070	H→L+1 (0.60), H-1→L (0.35)
ωB2PLYP	$S_0 \rightarrow S_2$	588.6	2.106	0.060	H→L+1 (0.60), H-1→L (0.35)
RSX-QIDH	$S_0 \rightarrow S_2$	581.2	2.133	0.056	H→L+1 (0.58), H-1→L (0.37)
ωB2GPPLYP	$S_0 \rightarrow S_2$	584.5	2.121	0.060	H→L+1 (0.58), H-1→L (0.37)
ωB88PP86	$S_0 \rightarrow S_2$	575.6	2.154	0.067	H→L+1 (0.60), H-1→L (0.35)
ωPBEPP86	$S_0 \rightarrow S_2$	571.0	2.171	0.063	H→L+1 (0.59), H-1→L (0.36)
DSD-BLYP	$S_0 \rightarrow S_2$	539.2	2.299	0.060	H→L+1 (0.59), H-1→L (0.37)
DSD-PBEP86	$S_0 \rightarrow S_2$	540.0	2.296	0.070	H→L+1 (0.60), H-1→L (0.36)
SCS/SOS-B2PLYP	$S_0 \rightarrow S_2$	507.6	2.442	0.100	H→L+1 (0.67), H-1→L (0.31)
SCS-B2GPPLYP	$S_0 \rightarrow S_2$	560.3	2.213	0.080	H→L+1 (0.63), H-1→L (0.34)
SOS-B2GPPLYP	$S_0 \rightarrow S_2$	579.1	2.141	0.070	H→L+1 (0.63), H-1→L (0.34)
SCS-PBE-QIDH	$S_0 \rightarrow S_2$	620.0	2.000	0.070	H→L+1 (0.62), H-1→L (0.35)
SOS-PBE-QIDH	$S_0 \rightarrow S_2$	636.4	1.948	0.060	H→L+1 (0.62), H-1→L (0.35)
SCS/SOS-ωB2PLYP	$S_0 \rightarrow S_2$	663.5	1.869	0.057	H→L+1 (0.60), H-1→L (0.35)
SCS-ωB2GPPLYP	$S_0 \rightarrow S_2$	721.0	1.720	0.046	H→L+1 (0.58), H-1→L (0.37)
SOS-ωB2GPPLYP	$S_0 \rightarrow S_2$	694.5	1.785	0.048	H→L+1 (0.58), H-1→L (0.37)
SCS-RSX-QIDH	$S_0 \rightarrow S_2$	777.3	1.595	0.040	H→L+1 (0.58), H-1→L (0.37)
SOS-RSX-QIDH	$S_0 \rightarrow S_2$	817.7	1.516	0.040	H→L+1 (0.58), H-1→L (0.37)
SCS-ωB88PP86	$S_0 \rightarrow S_2$	662.1	1.872	0.058	H→L+1 (0.60), H-1→L (0.35)
SOS-ωB88PP86	$S_0 \rightarrow S_2$	685.1	1.810	0.056	H→L+1 (0.60), H-1→L (0.35)
SCS-ωPBEPP86	$S_0 \rightarrow S_2$	756.0	1.640	0.048	H→L+1 (0.59), H-1→L (0.36)
SOS-ωPBEPP86	$S_0 \rightarrow S_2$	723.1	1.715	0.050	H→L+1 (0.59), H-1→L (0.36)

Table S28: Calculated Vertical Excitaion Energies (EE) in nm and eV, Oscillator Strength (f), and their MO Designation and Corresponding Coefficients, of the Q1 Band of Molecule **12**, based on BPE0/def2-TZVP/CPCM optimized geometry.

Functional	Electronic transition	EE (nm)	EE (eV)	f	MO designation (coefficient)
PBE0-DH	$S_0 \rightarrow S_2$	591.1	2.097	0.20	H→L+1 (0.75), H-1→L (0.23)
mPW2PLYP	$S_0 \rightarrow S_2$	572.5	2.166	0.49	H→L+1 (0.87), H-1→L (0.11)
B2PLYP	$S_0 \rightarrow S_2$	569.5	2.177	0.49	H→L+1 (0.87), H-1→L (0.11)
PBE-QIDH	$S_0 \rightarrow S_2$	600.8	2.064	0.27	H→L+1 (0.76), H-1→L (0.21)
B2GPPLYP	$S_0 \rightarrow S_2$	560.1	2.214	0.52	H→L+1 (0.86), H-1→L (0.11)
PBE0-2	$S_0 \rightarrow S_2$	597.3	2.076	0.30	H→L+1 (0.76), H-1→L (0.20)
RSX-0DH	$S_0 \rightarrow S_2$	647.4	1.915	0.40	H→L+1 (0.84), H-1→L (0.13)
ωB2PLYP	$S_0 \rightarrow S_2$	613.8	2.020	0.47	H→L+1 (0.84), H-1→L (0.12)
RSX-QIDH	$S_0 \rightarrow S_2$	598.6	2.071	0.50	H→L+1 (0.84), H-1→L (0.12)
ωB2GPPLYP	$S_0 \rightarrow S_2$	593.2	2.090	0.50	H→L+1 (0.84), H-1→L (0.12)
ωB88PP86	$S_0 \rightarrow S_2$	625.6	1.982	0.25	H→L+1 (0.74), H-1→L (0.22)
ωPBEPP86	$S_0 \rightarrow S_2$	616.1	2.012	0.27	H→L+1 (0.74), H-1→L (0.21)
DSD-BLYP	$S_0 \rightarrow S_2$	454.4	2.729	0.66	H→L+1 (0.85), H-1→L (0.11)
DSD-PBEP86	$S_0 \rightarrow S_2$	459.2	2.700	0.65	H→L+1 (0.86), H-1→L (0.11)
SCS/SOS-B2PLYP	$S_0 \rightarrow S_2$	538.0	2.304	0.30	H→L+1 (0.76), H-1→L (0.22)
SCS-B2GPPLYP	$S_0 \rightarrow S_2$	622.3	1.992	0.30	H→L+1 (0.76), H-1→L (0.21)
SOS-B2GPPLYP	$S_0 \rightarrow S_2$	647.7	1.914	0.30	H→L+1 (0.76), H-1→L (0.21)
SCS-PBE-QIDH	$S_0 \rightarrow S_2$	700.4	1.770	0.20	H→L+1 (0.76), H-1→L (0.21)
SOS-PBE-QIDH	$S_0 \rightarrow S_2$	734.2	1.689	0.20	H→L+1 (0.76), H-1→L (0.21)
SCS/SOS-ωB2PLYP	$S_0 \rightarrow S_2$	706.6	1.755	0.41	H→L+1 (0.84), H-1→L (0.12)
SCS-ωB2GPPLYP	$S_0 \rightarrow S_2$	743.3	1.668	0.40	H→L+1 (0.84), H-1→L (0.12)
SOS-ωB2GPPLYP	$S_0 \rightarrow S_2$	725.7	1.708	0.41	H→L+1 (0.83), H-1→L (0.12)
SCS-RSX-QIDH	$S_0 \rightarrow S_2$	751.4	1.650	0.45	H→L+1 (0.84), H-1→L (0.12)
SOS-RSX-QIDH	$S_0 \rightarrow S_2$	844.2	1.469	0.35	H→L+1 (0.84), H-1→L (0.12)
SCS-ωB88PP86	$S_0 \rightarrow S_2$	794.4	1.561	0.20	H→L+1 (0.74), H-1→L (0.22)
SOS-ωB88PP86	$S_0 \rightarrow S_2$	848.2	1.462	0.19	H→L+1 (0.74), H-1→L (0.21)
SCS-ωPBEPP86	$S_0 \rightarrow S_2$	913.2	1.358	0.18	H→L+1 (0.74), H-1→L (0.21)
SOS-ωPBEPP86	$S_0 \rightarrow S_2$	919.5	1.348	0.18	H→L+1 (0.74), H-1→L (0.21)

Table S29: Calculated Vertical Excitaion Energies (EE) in nm and eV, Oscillator Strength (f), and their MO Designation and Corresponding Coefficients, of the Q1 Band of Molecule **13**, based on BPE0/def2-TZVP/CPCM optimized geometry.

Functional	Electronic transition	EE (nm)	EE (eV)	f	MO designation (coefficient)
PBE0-DH	$S_0 \rightarrow S_1$	574.2	2.159	0.40	H→L+1 (0.84)
mPW2PLYP	$S_0 \rightarrow S_1$	573.8	2.161	0.41	H→L+1 (0.84)
B2PLYP	$S_0 \rightarrow S_1$	572.2	2.167	0.41	H→L+1 (0.84)
PBE-QIDH	$S_0 \rightarrow S_1$	569.3	2.178	0.43	H→L+1 (0.83)
B2GPPLYP	$S_0 \rightarrow S_1$	567.0	2.187	0.43	H→L+1 (0.83)
PBE0-2	$S_0 \rightarrow S_1$	554.3	2.237	0.47	H→L+1 (0.82)
RSX-0DH	$S_0 \rightarrow S_1$	642.7	1.929	0.36	H→L+1 (0.78)
ωB2PLYP	$S_0 \rightarrow S_1$	618.3	2.005	0.38	H→L+1 (0.79)
RSX-QIDH	$S_0 \rightarrow S_1$	606.1	2.045	0.41	H→L+1 (0.80)
ωB2GPPLYP	$S_0 \rightarrow S_1$	602.7	2.057	0.40	H→L+1 (0.80)
ωB88PP86	$S_0 \rightarrow S_1$	579.9	2.138	0.42	H→L+1 (0.80)
ωPBEPP86	$S_0 \rightarrow S_1$	568.9	2.179	0.43	H→L+1 (0.81)
DSD-BLYP	$S_0 \rightarrow S_1$	487.9	2.541	0.53	H→L+1 (0.83)
DSD-PBEP86	$S_0 \rightarrow S_1$	491.5	2.522	0.51	H→L+1 (0.83)
SCS/SOS-B2PLYP	$S_0 \rightarrow S_1$	519.2	2.388	0.45	H→L+1 (0.84)
SCS-B2GPPLYP	$S_0 \rightarrow S_1$	580.7	2.135	0.40	H→L+1 (0.83)
SOS-B2GPPLYP	$S_0 \rightarrow S_1$	600.5	2.065	0.40	H→L+1 (0.83)
SCS-PBE-QIDH	$S_0 \rightarrow S_1$	639.5	1.939	0.38	H→L+1 (0.83)
SOS-PBE-QIDH	$S_0 \rightarrow S_1$	662.3	1.872	0.37	H→L+1 (0.83)
SCS/SOS-ωB2PLYP	$S_0 \rightarrow S_1$	731.7	1.694	0.32	H→L+1 (0.79)
SCS-ωB2GPPLYP	$S_0 \rightarrow S_1$	785.6	1.578	0.31	H→L+1 (0.80)
SOS-ωB2GPPLYP	$S_0 \rightarrow S_1$	766.5	1.617	0.32	H→L+1 (0.80)
SCS-RSX-QIDH	$S_0 \rightarrow S_1$	792.9	1.564	0.31	H→L+1 (0.80)
SOS-RSX-QIDH	$S_0 \rightarrow S_1$	921.5	1.345	0.27	H→L+1 (0.80)
SCS-ωB88PP86	$S_0 \rightarrow S_1$	704.7	1.759	0.34	H→L+1 (0.81)
SOS-ωB88PP86	$S_0 \rightarrow S_1$	737.6	1.681	0.33	H→L+1 (0.81)
SCS-ωPBEPP86	$S_0 \rightarrow S_1$	793.9	1.562	0.31	H→L+1 (0.81)
SOS-ωPBEPP86	$S_0 \rightarrow S_1$	781.4	1.587	0.32	H→L+1 (0.81)

S5 Calculated Vertical Excitaion Energies of Q3 Band

Table S30: Calculated Vertical Excitaion Energies (EE) in nm and eV, Oscillator Strength (f), and their MO Designation and Corresponding Coefficients, of the Q3 Band of Molecule **1**, based on BPE0/def2-TZVP/CPCM optimized geometry.

Functional	Electronic transition	EE (nm)	EE (eV)	f	MO designation (coefficient)
PBE0-DH	$S_0 \rightarrow S_1$	546.3	2.270	0.007	H→L (0.49), H-1→L+1 (0.48)
mPW2PLYP	$S_0 \rightarrow S_1$	557.9	2.222	0.010	H→L (0.51), H-1→L+1 (0.46)
B2PLYP	$S_0 \rightarrow S_1$	558.8	2.219	0.009	H→L (0.51), H-1→L+1 (0.46)
PBE-QIDH	$S_0 \rightarrow S_1$	561.6	2.208	0.020	H→L (0.55), H-1→L+1 (0.42)
B2GPPLYP	$S_0 \rightarrow S_1$	561.9	2.206	0.017	H→L (0.54), H-1→L+1 (0.43)
PBE0-2	$S_0 \rightarrow S_1$	562.6	2.204	0.029	H→L (0.57), H-1→L+1 (0.40)
RSX-0DH	$S_0 \rightarrow S_1$	638.3	1.943	0.016	H→L (0.54), H-1→L+1 (0.42)
ωB2PLYP	$S_0 \rightarrow S_1$	625.5	1.982	0.020	H→L (0.54), H-1→L+1 (0.42)
RSX-QIDH	$S_0 \rightarrow S_1$	614.3	2.018	0.020	H→L (0.56), H-1→L+1 (0.40)
ωB2GPPLYP	$S_0 \rightarrow S_1$	614.1	2.019	0.020	H→L (0.56), H-1→L+1 (0.41)
ωB88PP86	$S_0 \rightarrow S_1$	589.3	2.104	0.019	H→L (0.55), H-1→L+1 (0.42)
ωPBEPP86	$S_0 \rightarrow S_1$	581.2	2.133	0.020	H→L (0.55), H-1→L+1 (0.41)
DSD-BLYP	$S_0 \rightarrow S_1$	530.3	2.338	0.030	H→L (0.56), H-1→L+1 (0.40)
DSD-PBEP86	$S_0 \rightarrow S_1$	533.3	2.325	0.020	H→L (0.56), H-1→L+1 (0.41)
SCS/SOS-B2PLYP	$S_0 \rightarrow S_1$	508.1	2.440	0.009	H→L (0.51), H-1→L+1 (0.46)
SCS-B2GPPLYP	$S_0 \rightarrow S_1$	593.3	2.090	0.016	H→L (0.54), H-1→L+1 (0.43)
SOS-B2GPPLYP	$S_0 \rightarrow S_1$	616.8	2.010	0.016	H→L (0.54), H-1→L+1 (0.43)
SCS-PBE-QIDH	$S_0 \rightarrow S_1$	664.6	1.866	0.017	H→L (0.55), H-1→L+1 (0.42)
SOS-PBE-QIDH	$S_0 \rightarrow S_1$	702.3	1.765	0.016	H→L (0.55), H-1→L+1 (0.42)
SCS/SOS-ωB2PLYP	$S_0 \rightarrow S_1$	794.8	1.560	0.014	H→L (0.54), H-1→L+1 (0.42)
SCS-ωB2GPPLYP	$S_0 \rightarrow S_1$	863.4	1.436	0.016	H→L (0.56), H-1→L+1 (0.40)
SOS-ωB2GPPLYP	$S_0 \rightarrow S_1$	866.6	1.431	0.016	H→L (0.56), H-1→L+1 (0.41)
SCS-RSX-QIDH	$S_0 \rightarrow S_1$	808.6	1.533	0.018	H→L (0.56), H-1→L+1 (0.40)
SOS-RSX-QIDH	$S_0 \rightarrow S_1$	1135.6	1.092	0.013	H→L (0.56), H-1→L+1 (0.40)
SCS-ωB88PP86	$S_0 \rightarrow S_1$	748.6	1.656	0.015	H→L (0.55), H-1→L+1 (0.42)
SOS-ωB88PP86	$S_0 \rightarrow S_1$	809.3	1.532	0.014	H→L (0.55), H-1→L+1 (0.42)
SCS-ωPBEPP86	$S_0 \rightarrow S_1$	831.3	1.491	0.015	H→L (0.55), H-1→L+1 (0.41)
SOS-ωPBEPP86	$S_0 \rightarrow S_1$	879.5	1.410	0.014	H→L (0.55), H-1→L+1 (0.41)

Table S31: Calculated Vertical Excitaion Energies (EE) in nm and eV, Oscillator Strength (f), and their MO Designation and Corresponding Coefficients, of the Q3 Band of Molecule **4**, based on BPE0/def2-TZVP/CPCM optimized geometry.

Functional	Electronic transition	EE (nm)	EE (eV)	f	MO designation (coefficient)
PBE0-DH	$S_0 \rightarrow S_1$	554.1	2.237	0.040	H→L (0.58), H-1→L+1 (0.40)
mPW2PLYP	$S_0 \rightarrow S_1$	563.7	2.199	0.042	H→L (0.60), H-1→L+1 (0.38)
B2PLYP	$S_0 \rightarrow S_1$	563.9	2.199	0.040	H→L (0.60), H-1→L+1 (0.39)
PBE-QIDH	$S_0 \rightarrow S_1$	565.1	2.194	0.060	H→L (0.63), H-1→L+1 (0.35)
B2GPPLYP	$S_0 \rightarrow S_1$	564.9	2.195	0.055	H→L (0.62), H-1→L+1 (0.35)
PBE0-2	$S_0 \rightarrow S_1$	560.9	2.210	0.070	H→L (0.64), H-1→L+1 (0.32)
RSX-0DH	$S_0 \rightarrow S_1$	648.4	1.912	0.047	H→L (0.61), H-1→L+1 (0.35)
ωB2PLYP	$S_0 \rightarrow S_1$	631.3	1.964	0.049	H→L (0.61), H-1→L+1 (0.35)
RSX-QIDH	$S_0 \rightarrow S_1$	617.0	2.009	0.060	H→L (0.63), H-1→L+1 (0.33)
ωB2GPPLYP	$S_0 \rightarrow S_1$	616.5	2.011	0.058	H→L (0.63), H-1→L+1 (0.34)
ωB88PP86	$S_0 \rightarrow S_1$	590.3	2.100	0.056	H→L (0.62), H-1→L+1 (0.35)
ωPBEPP86	$S_0 \rightarrow S_1$	580.4	2.136	0.060	H→L (0.63), H-1→L+1 (0.34)
DSD-BLYP	$S_0 \rightarrow S_1$	516.9	2.398	0.075	H→L (0.64), H-1→L+1 (0.33)
DSD-PBEP86	$S_0 \rightarrow S_1$	520.4	2.382	0.069	H→L (0.63), H-1→L+1 (0.34)
SCS/SOS-B2PLYP	$S_0 \rightarrow S_1$	510.9	2.427	0.044	H→L (0.60), H-1→L+1 (0.39)
SCS-B2GPPLYP	$S_0 \rightarrow S_1$	594.7	2.085	0.050	H→L (0.62), H-1→L+1 (0.35)
SOS-B2GPPLYP	$S_0 \rightarrow S_1$	619.2	2.002	0.050	H→L (0.62), H-1→L+1 (0.35)
SCS-PBE-QIDH	$S_0 \rightarrow S_1$	668.0	1.856	0.050	H→L (0.63), H-1→L+1 (0.35)
SOS-PBE-QIDH	$S_0 \rightarrow S_1$	704.6	1.760	0.046	H→L (0.63), H-1→L+1 (0.35)
SCS/SOS-ωB2PLYP	$S_0 \rightarrow S_1$	804.2	1.542	0.038	H→L (0.61), H-1→L+1 (0.35)
SCS-ωB2GPPLYP	$S_0 \rightarrow S_1$	880.4	1.408	0.040	H→L (0.63), H-1→L+1 (0.34)
SOS-ωB2GPPLYP	$S_0 \rightarrow S_1$	872.3	1.421	0.041	H→L (0.63), H-1→L+1 (0.34)
SCS-RSX-QIDH	$S_0 \rightarrow S_1$	841.5	1.473	0.045	H→L (0.63), H-1→L+1 (0.33)
SOS-RSX-QIDH	$S_0 \rightarrow S_1$	1144.2	1.084	0.030	H→L (0.63), H-1→L+1 (0.33)
SCS-ωB88PP86	$S_0 \rightarrow S_1$	757.9	1.636	0.043	H→L (0.62), H-1→L+1 (0.35)
SOS-ωB88PP86	$S_0 \rightarrow S_1$	814.9	1.521	0.040	H→L (0.62), H-1→L+1 (0.35)
SCS-ωPBEPP86	$S_0 \rightarrow S_1$	858.2	1.445	0.041	H→L (0.63), H-1→L+1 (0.34)
SOS-ωPBEPP86	$S_0 \rightarrow S_1$	884.8	1.401	0.040	H→L (0.63), H-1→L+1 (0.34)

Table S32: Calculated Vertical Excitaion Energies (EE) in nm and eV, Oscillator Strength (f), and their MO Designation and Corresponding Coefficients, of the Q3 Band of Molecule **7**, based on BPE0/def2-TZVP/CPCM optimized geometry.

Functional	Electronic transition	EE (nm)	EE (eV)	f	MO designation (coefficient)
PBE0-DH	$S_0 \rightarrow S_1$	574.1	2.160	0.00600	H→L (0.57), H-1→L+1 (0.41)
mPW2PLYP	$S_0 \rightarrow S_1$	586.2	2.115	0.00300	H→L (0.55), H-1→L+1 (0.43)
B2PLYP	$S_0 \rightarrow S_1$	587.9	2.109	0.00350	H→L (0.56), H-1→L+1 (0.42)
PBE-QIDH	$S_0 \rightarrow S_1$	584.4	2.431	0.00001	H→L (0.49), H-1→L+1 (0.47)
B2GPPLYP	$S_0 \rightarrow S_1$	586.5	2.114	0.00012	H→L (0.51), H-1→L+1 (0.46)
PBE0-2	$S_0 \rightarrow S_1$	579.3	2.140	0.00200	H→L (0.50), H-1→L+1 (0.46)
RSX-0DH	$S_0 \rightarrow S_1$	665.3	1.864	0.00001	H→L (0.43), H-1→L+1 (0.42)
ωB2PLYP	$S_0 \rightarrow S_1$	650.4	1.906	0.00001	H→L (0.48), H-1→L+1 (0.47)
RSX-QIDH	$S_0 \rightarrow S_1$	634.2	1.955	0.00100	H→L (0.48), H-1→L+1 (0.44)
ωB2GPPLYP	$S_0 \rightarrow S_1$	635.0	1.952	0.00040	H→L (0.49), H-1→L+1 (0.47)
ωB88PP86	$S_0 \rightarrow S_1$	610.7	2.030	0.00005	H→L (0.48), H-1→L+1 (0.48)
ωPBEPP86	$S_0 \rightarrow S_1$	600.5	2.065	0.00035	H→L (0.48), H-1→L+1 (0.47)
DSD-BLYP	$S_0 \rightarrow S_1$	543.7	2.281	0.00080	H→L (0.49), H-1→L+1 (0.47)
DSD-PBEP86	$S_0 \rightarrow S_1$	549.9	2.255	0.00020	H→L (0.48), H-1→L+1 (0.48)
SCS/SOS-B2PLYP	$S_0 \rightarrow S_1$	525.4	2.360	0.00400	H→L (0.55), H-1→L+1 (0.42)
SCS-B2GPPLYP	$S_0 \rightarrow S_1$	615.7	2.014	0.00010	H→L (0.51), H-1→L+1 (0.46)
SOS-B2GPPLYP	$S_0 \rightarrow S_1$	643.2	1.928	0.00010	H→L (0.51), H-1→L+1 (0.46)
SCS-PBE-QIDH	$S_0 \rightarrow S_1$	699.0	1.774	0.00008	H→L (0.49), H-1→L+1 (0.47)
SOS-PBE-QIDH	$S_0 \rightarrow S_1$	739.3	1.677	0.00008	H→L (0.49), H-1→L+1 (0.47)
SCS/SOS-ωB2PLYP	$S_0 \rightarrow S_1$	842.0	1.473	0.00001	H→L (0.48), H-1→L+1 (0.47)
SCS-ωB2GPPLYP	$S_0 \rightarrow S_1$	936.7	1.324	0.00030	H→L (0.49), H-1→L+1 (0.47)
SOS-ωB2GPPLYP	$S_0 \rightarrow S_1$	922.6	1.344	0.00034	H→L (0.49), H-1→L+1 (0.47)
SCS-RSX-QIDH	$S_0 \rightarrow S_1$	902.3	1.374	0.00070	H→L (0.48), H-1→L+1 (0.44)
SOS-RSX-QIDH	$S_0 \rightarrow S_1$	1249.8	0.992	0.00050	H→L (0.48), H-1→L+1 (0.44)
SCS-ωB88PP86	$S_0 \rightarrow S_1$	797.1	1.555	0.00004	H→L (0.48), H-1→L+1 (0.48)
SOS-ωB88PP86	$S_0 \rightarrow S_1$	860.2	1.441	0.00004	H→L (0.48), H-1→L+1 (0.48)
SCS-ωPBEPP86	$S_0 \rightarrow S_1$	921.4	1.346	0.00020	H→L (0.48), H-1→L+1 (0.47)
SOS-ωPBEPP86	$S_0 \rightarrow S_1$	942.7	1.832	0.00020	H→L (0.48), H-1→L+1 (0.47)

Table S33: Calculated Vertical Excitaion Energies (EE) in nm and eV, Oscillator Strength (f), and their MO Designation and Corresponding Coefficients, of the Q3 Band of Molecule **10**, based on BPE0/def2-TZVP/CPCM optimized geometry.

Functional	Electronic transition	EE (nm)	EE (eV)	f	MO designation (coefficient)
PBE0-DH	$S_0 \rightarrow S_1$	567.2	2.186	0.0005	H→L+1 (0.53), H-1→L (0.44)
mPW2PLYP	$S_0 \rightarrow S_1$	578.7	2.143	0.0002	H→L+1 (0.50), H-1→L (0.44)
B2PLYP	$S_0 \rightarrow S_1$	580.0	2.138	0.0001	H→L+1 (0.52), H-1→L (0.45)
PBE-QIDH	$S_0 \rightarrow S_1$	576.7	2.150	0.0020	H→L+1 (0.50), H-1→L (0.46)
B2GPPLYP	$S_0 \rightarrow S_1$	578.5	2.143	0.0010	H→L+1 (0.49), H-1→L (0.48)
PBE0-2	$S_0 \rightarrow S_1$	570.9	2.172	0.0070	H→L+1 (0.53), H-1→L (0.43)
RSX-0DH	$S_0 \rightarrow S_1$	658.8	1.882	0.0021	H→L+1 (0.50), H-1→L (0.45)
ωB2PLYP	$S_0 \rightarrow S_1$	643.1	1.928	0.0017	H→L+1 (0.50), H-1→L (0.46)
RSX-QIDH	$S_0 \rightarrow S_1$	626.3	1.979	0.0055	H→L+1 (0.52), H-1→L (0.43)
ωB2GPPLYP	$S_0 \rightarrow S_1$	627.2	1.977	0.0040	H→L+1 (0.51), H-1→L (0.44)
ωB88PP86	$S_0 \rightarrow S_1$	602.3	2.059	0.0030	H→L+1 (0.50), H-1→L (0.46)
ωPBEPP86	$S_0 \rightarrow S_1$	591.7	2.095	0.0040	H→L+1 (0.51), H-1→L (0.45)
DSD-BLYP	$S_0 \rightarrow S_1$	531.6	2.332	0.0050	H→L+1 (0.52), H-1→L (0.44)
DSD-PBEP86	$S_0 \rightarrow S_1$	537.4	2.307	0.0040	H→L+1 (0.51), H-1→L (0.46)
SCS/SOS-B2PLYP	$S_0 \rightarrow S_1$	519.7	2.385	0.0001	H→L+1 (0.52), H-1→L (0.45)
SCS-B2GPPLYP	$S_0 \rightarrow S_1$	608.4	2.038	0.0010	H→L+1 (0.49), H-1→L (0.48)
SOS-B2GPPLYP	$S_0 \rightarrow S_1$	635.4	1.951	0.0010	H→L+1 (0.49), H-1→L (0.48)
SCS-PBE-QIDH	$S_0 \rightarrow S_1$	689.2	1.799	0.0020	H→L+1 (0.50), H-1→L (0.46)
SOS-PBE-QIDH	$S_0 \rightarrow S_1$	728.7	1.701	0.0020	H→L+1 (0.50), H-1→L (0.46)
SCS/SOS-ωB2PLYP	$S_0 \rightarrow S_1$	831.2	1.492	0.0014	H→L+1 (0.50), H-1→L (0.46)
SCS-ωB2GPPLYP	$S_0 \rightarrow S_1$	923.0	1.343	0.0010	H→L+1 (0.51), H-1→L (0.44)
SOS-ωB2GPPLYP	$S_0 \rightarrow S_1$	908.4	1.365	0.0030	H→L+1 (0.51), H-1→L (0.44)
SCS-RSX-QIDH	$S_0 \rightarrow S_1$	888.6	1.395	0.0040	H→L+1 (0.52), H-1→L (0.43)
SOS-RSX-QIDH	$S_0 \rightarrow S_1$	1220.1	1.016	0.0030	H→L+1 (0.52), H-1→L (0.43)
SCS-ωB88PP86	$S_0 \rightarrow S_1$	785.9	1.578	0.0020	H→L+1 (0.50), H-1→L (0.46)
SOS-ωB88PP86	$S_0 \rightarrow S_1$	846.9	1.464	0.0020	H→L+1 (0.50), H-1→L (0.46)
SCS-ωPBEPP86	$S_0 \rightarrow S_1$	907.1	1.367	0.0020	H→L+1 (0.51), H-1→L (0.45)
SOS-ωPBEPP86	$S_0 \rightarrow S_1$	926.0	1.339	0.0030	H→L+1 (0.51), H-1→L (0.45)

Table S34: Calculated Vertical Excitaion Energies (EE) in nm and eV, Oscillator Strength (f), and their MO Designation and Corresponding Coefficients, of the Q3 Band of Molecule **11**, based on BPE0/def2-TZVP/CPCM optimized geometry.

Functional	Electronic transition	EE (nm)	EE (eV)	f	MO designation (coefficient)
PBE0-DH	$S_0 \rightarrow S_1$	658.0	1.884	0.100	H→L (0.80), H-1→L+1 (0.17)
mPW2PLYP	$S_0 \rightarrow S_1$	674.8	1.837	0.130	H→L (0.78), H-1→L+1 (0.19)
B2PLYP	$S_0 \rightarrow S_1$	678.5	1.827	0.130	H→L (0.79), H-1→L+1 (0.18)
PBE-QIDH	$S_0 \rightarrow S_1$	671.9	1.845	0.097	H→L (0.73), H-1→L+1 (0.23)
B2GPPLYP	$S_0 \rightarrow S_1$	677.7	1.830	0.100	H→L (0.75), H-1→L+1 (0.22)
PBE0-2	$S_0 \rightarrow S_1$	676.8	1.832	0.070	H→L (0.69), H-1→L+1 (0.25)
RSX-0DH	$S_0 \rightarrow S_1$	739.2	1.677	0.060	H→L (0.69), H-1→L+1 (0.25)
ωB2PLYP	$S_0 \rightarrow S_1$	738.1	1.680	0.070	H→L (0.69), H-1→L+1 (0.25)
RSX-QIDH	$S_0 \rightarrow S_1$	722.4	1.716	0.065	H→L (0.68), H-1→L+1 (0.27)
ωB2GPPLYP	$S_0 \rightarrow S_1$	728.0	1.703	0.070	H→L (0.68), H-1→L+1 (0.26)
ωB88PP86	$S_0 \rightarrow S_1$	707.1	1.753	0.081	H→L (0.70), H-1→L+1 (0.24)
ωPBEPP86	$S_0 \rightarrow S_1$	700.1	1.771	0.078	H→L (0.70), H-1→L+1 (0.25)
DSD-BLYP	$S_0 \rightarrow S_1$	674.8	1.837	0.080	H→L (0.70), H-1→L+1 (0.25)
DSD-PBEP86	$S_0 \rightarrow S_1$	678.6	1.827	0.090	H→L (0.72), H-1→L+1 (0.23)
SCS/SOS-B2PLYP	$S_0 \rightarrow S_1$	589.6	2.103	0.100	H→L (0.79), H-1→L+1 (0.18)
SCS-B2GPPLYP	$S_0 \rightarrow S_1$	692.1	1.791	0.100	H→L (0.75), H-1→L+1 (0.22)
SOS-B2GPPLYP	$S_0 \rightarrow S_1$	724.7	1.711	0.100	H→L (0.75), H-1→L+1 (0.22)
SCS-PBE-QIDH	$S_0 \rightarrow S_1$	798.7	1.552	0.080	H→L (0.74), H-1→L+1 (0.23)
SOS-PBE-QIDH	$S_0 \rightarrow S_1$	840.2	1.476	0.080	H→L (0.74), H-1→L+1 (0.23)
SCS/SOS-ωB2PLYP	$S_0 \rightarrow S_1$	942.8	1.315	0.058	H→L (0.69), H-1→L+1 (0.25)
SCS-ωB2GPPLYP	$S_0 \rightarrow S_1$	1072.3	1.156	0.046	H→L (0.68), H-1→L+1 (0.26)
SOS-ωB2GPPLYP	$S_0 \rightarrow S_1$	1040.2	1.192	0.048	H→L (0.68), H-1→L+1 (0.26)
SCS-RSX-QIDH	$S_0 \rightarrow S_1$	1090.0	1.137	0.040	H→L (0.68), H-1→L+1 (0.27)
SOS-RSX-QIDH	$S_0 \rightarrow S_1$	1437.9	0.862	0.030	H→L (0.68), H-1→L+1 (0.27)
SCS-ωB88PP86	$S_0 \rightarrow S_1$	908.6	1.365	0.063	H→L (0.70), H-1→L+1 (0.24)
SOS-ωB88PP86	$S_0 \rightarrow S_1$	977.1	1.269	0.059	H→L (0.70), H-1→L+1 (0.24)
SCS-ωPBEPP86	$S_0 \rightarrow S_1$	1086.5	1.141	0.051	H→L (0.70), H-1→L+1 (0.25)
SOS-ωPBEPP86	$S_0 \rightarrow S_1$	1079.2	1.149	0.051	H→L (0.70), H-1→L+1 (0.25)

Table S35: Calculated Vertical Excitaion Energies (EE) in nm and eV, Oscillator Strength (f), and their MO Designation and Corresponding Coefficients, of the Q3 Band of Molecule **12**, based on BPE0/def2-TZVP/CPCM optimized geometry.

Functional	Electronic transition	EE (nm)	EE (eV)	f	MO designation (coefficient)
PBE0-DH	$S_0 \rightarrow S_1$	579.1	2.141	0.50	H→L (0.87), H-1→L+1 (0.11)
mPW2PLYP	$S_0 \rightarrow S_1$	599.6	2.068	0.25	H→L (0.76), H-1→L+1 (0.22)
B2PLYP	$S_0 \rightarrow S_1$	599.0	2.069	0.24	H→L (0.76), H-1→L+1 (0.22)
PBE-QIDH	$S_0 \rightarrow S_1$	564.2	2.198	0.52	H→L (0.86), H-1→L+1 (0.11)
B2GPPLYP	$S_0 \rightarrow S_1$	600.4	2.065	0.27	H→L (0.76), H-1→L+1 (0.21)
PBE0-2	$S_0 \rightarrow S_1$	539.6	2.298	0.60	H→L (0.85), H-1→L+1 (0.12)
RSX-0DH	$S_0 \rightarrow S_1$	681.5	1.819	0.20	H→L (0.72), H-1→L+1 (0.23)
ωB2PLYP	$S_0 \rightarrow S_1$	665.5	1.863	0.22	H→L (0.73), H-1→L+1 (0.23)
RSX-QIDH	$S_0 \rightarrow S_1$	652.2	1.901	0.26	H→L (0.74), H-1→L+1 (0.22)
ωB2GPPLYP	$S_0 \rightarrow S_1$	651.9	1.902	0.25	H→L (0.74), H-1→L+1 (0.22)
ωB88PP86	$S_0 \rightarrow S_1$	568.8	2.180	0.51	H→L (0.84), H-1→L+1 (0.12)
ωPBEPP86	$S_0 \rightarrow S_1$	555.1	2.180	0.53	H→L (0.84), H-1→L+1 (0.12)
DSD-BLYP	$S_0 \rightarrow S_1$	550.9	2.251	0.31	H→L (0.76), H-1→L+1 (0.20)
DSD-PBEP86	$S_0 \rightarrow S_1$	553.1	2.241	0.30	H→L (0.76), H-1→L+1 (0.21)
SCS/SOS-B2PLYP	$S_0 \rightarrow S_1$	519.5	2.387	0.50	H→L (0.87), H-1→L+1 (0.11)
SCS-B2GPPLYP	$S_0 \rightarrow S_1$	569.7	2.176	0.50	H→L (0.86), H-1→L+1 (0.11)
SOS-B2GPPLYP	$S_0 \rightarrow S_1$	587.0	2.112	0.50	H→L (0.86), H-1→L+1 (0.11)
SCS-PBE-QIDH	$S_0 \rightarrow S_1$	619.3	2.002	0.50	H→L (0.86), H-1→L+1 (0.11)
SOS-PBE-QIDH	$S_0 \rightarrow S_1$	637.7	1.944	0.46	H→L (0.86), H-1→L+1 (0.11)
SCS/SOS-ωB2PLYP	$S_0 \rightarrow S_1$	834.8	1.485	0.18	H→L (0.73), H-1→L+1 (0.23)
SCS-ωB2GPPLYP	$S_0 \rightarrow S_1$	920.3	1.347	0.18	H→L (0.74), H-1→L+1 (0.22)
SOS-ωB2GPPLYP	$S_0 \rightarrow S_1$	902.3	1.374	0.18	H→L (0.74), H-1→L+1 (0.22)
SCS-RSX-QIDH	$S_0 \rightarrow S_1$	907.3	1.367	0.18	H→L (0.74), H-1→L+1 (0.22)
SOS-RSX-QIDH	$S_0 \rightarrow S_1$	1173.3	1.057	0.14	H→L (0.74), H-1→L+1 (0.22)
SCS-ωB88PP86	$S_0 \rightarrow S_1$	676.8	1.832	0.43	H→L (0.84), H-1→L+1 (0.12)
SOS-ωB88PP86	$S_0 \rightarrow S_1$	704.1	1.761	0.41	H→L (0.84), H-1→L+1 (0.12)
SCS-ωPBEPP86	$S_0 \rightarrow S_1$	751.2	1.650	0.39	H→L (0.84), H-1→L+1 (0.12)
SOS-ωPBEPP86	$S_0 \rightarrow S_1$	737.0	1.682	0.40	H→L (0.84), H-1→L+1 (0.12)

S6 Molecular Orbital Plots

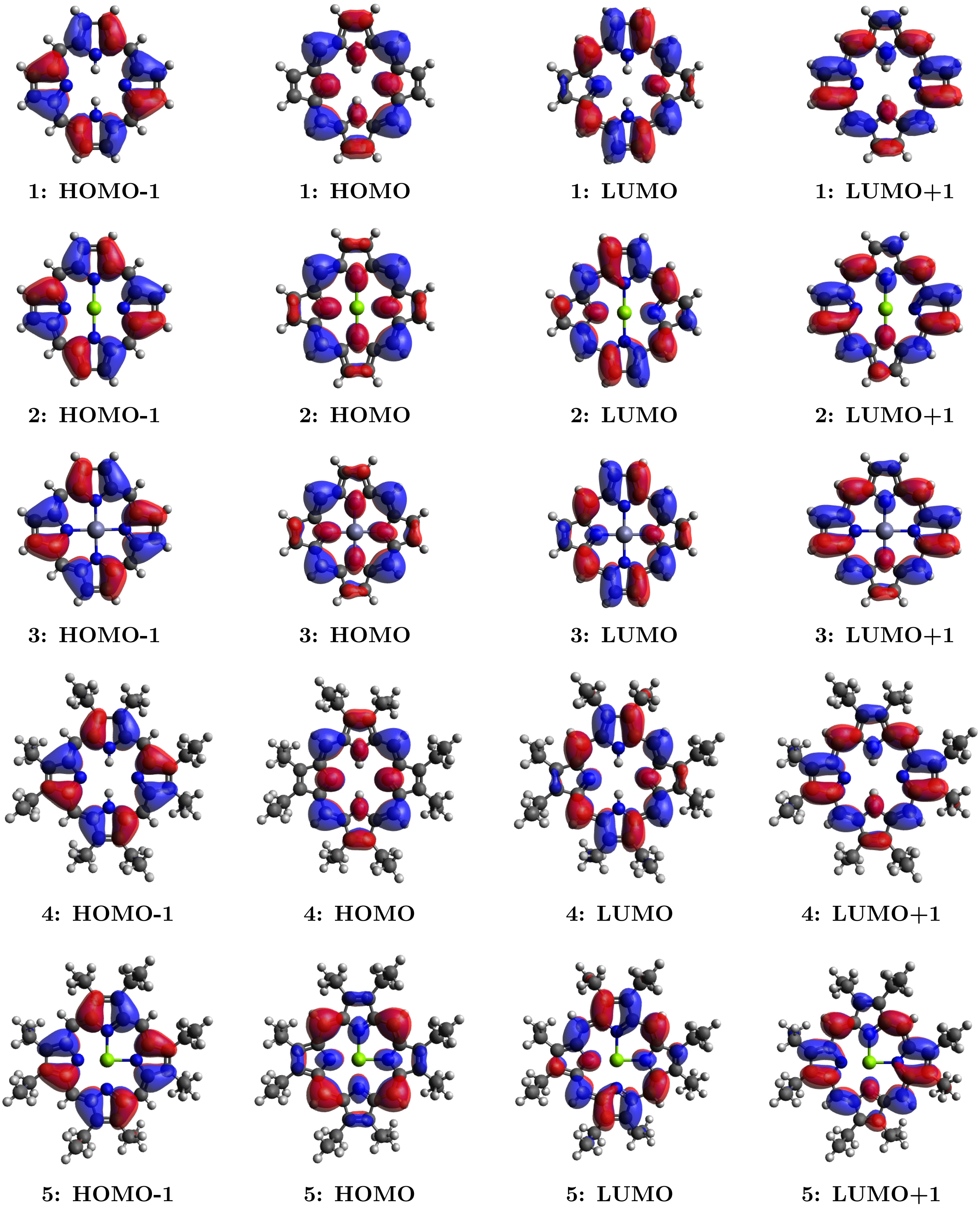

Figure S1: Frontier MOs (HOMO-1, HOMO, LUOM and LUMO+1) involved in the main transitions of porphyrins **1–5**. Isosurface value 0.02.

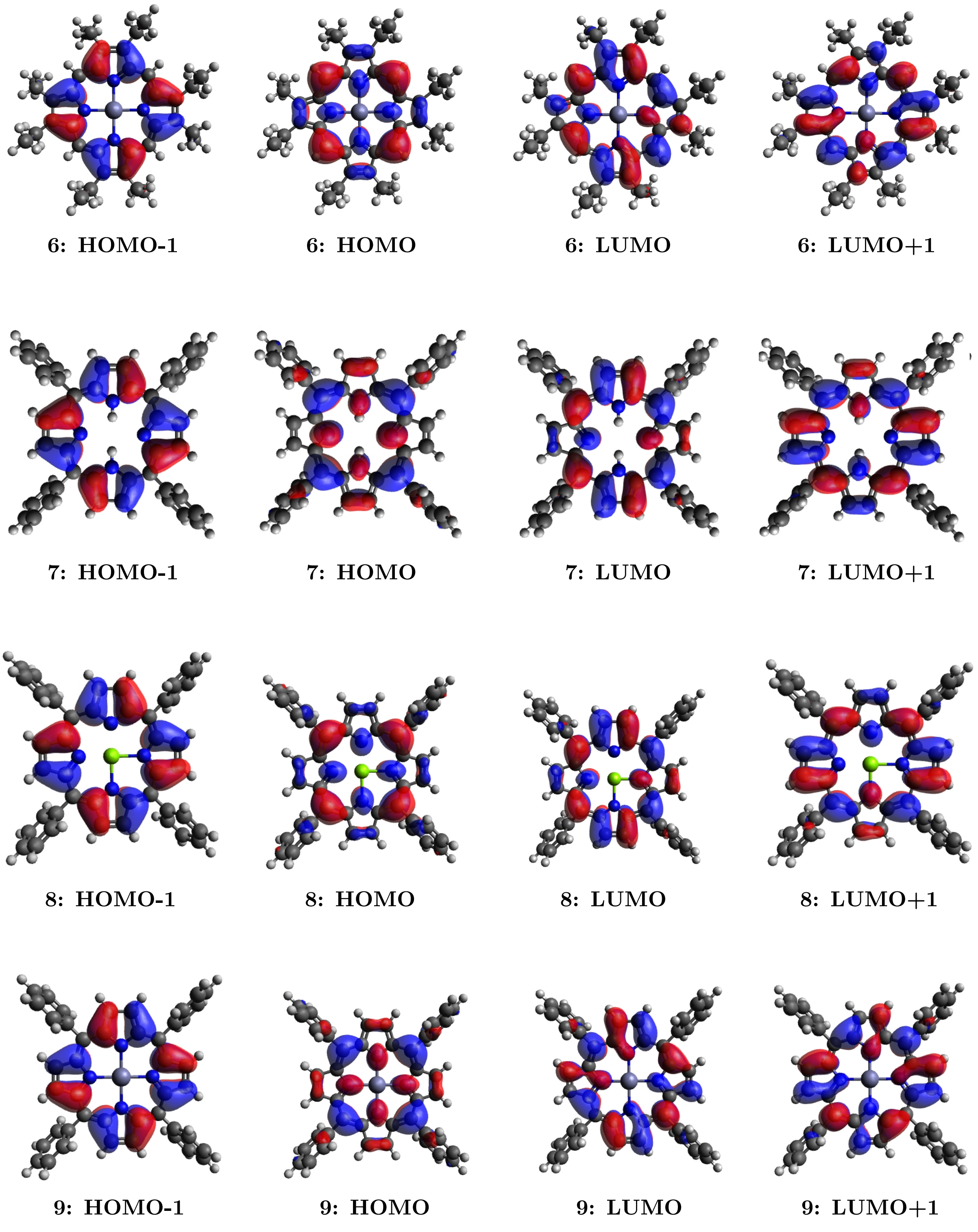

Figure S2: Frontier MOs (HOMO-1, HOMO, LUOM and LUMO+1) involved in the main transitions of porphyrins **6–9**. Isosurface value 0.02.

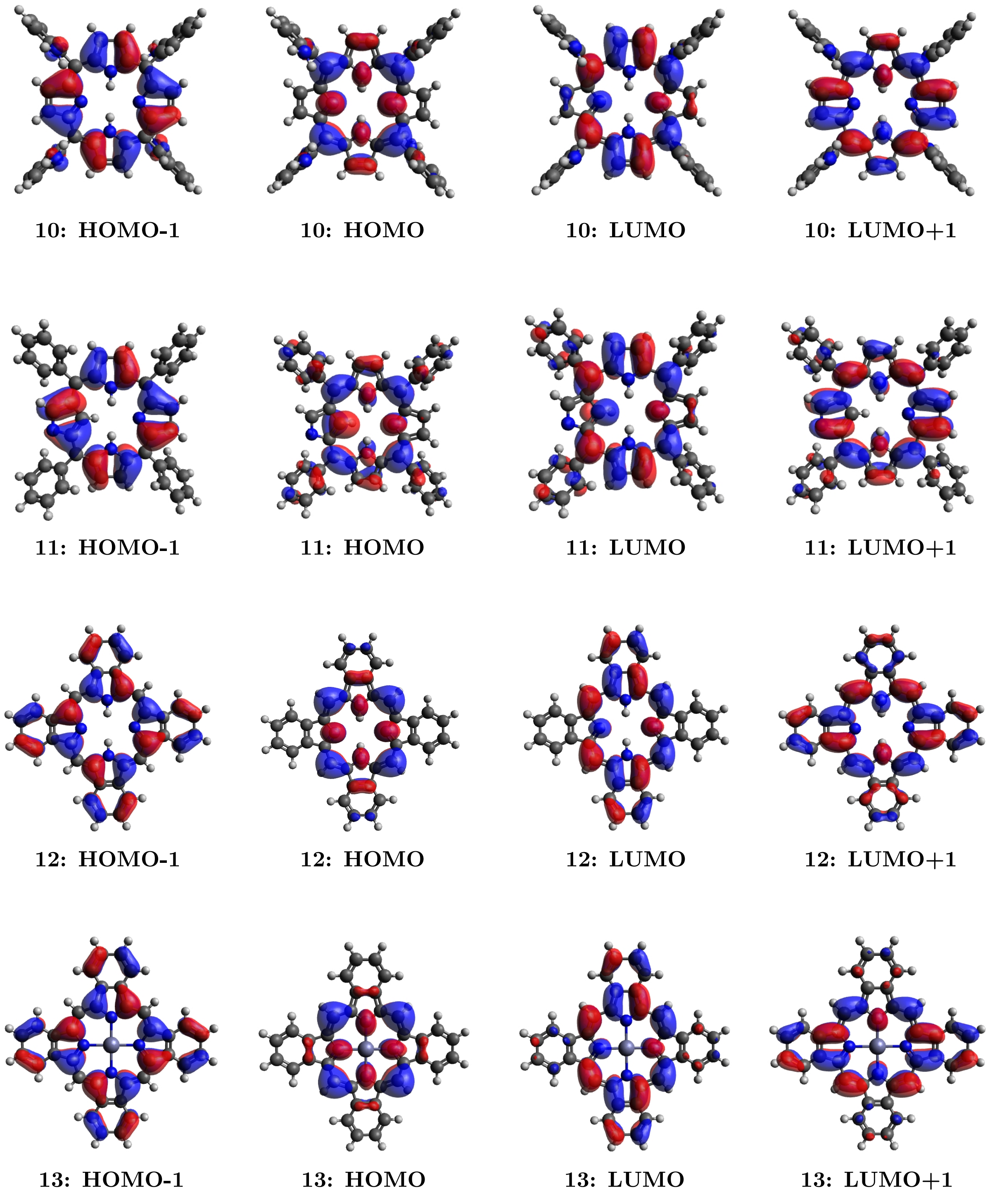

Figure S3: Frontier MOs (HOMO-1, HOMO, LUOM and LUMO+1) involved in the main transitions of porphyrins **10–13**. Isosurface value 0.02.

S7 Comparison of Calculated and Experimnetal Excitaion Energies: The B Band

Table S36: Vertical Excitation Energies (eV) of the B Band (Soret) Using GDH Density Functionals with the def2-TZVP Basis Set and CPCM solvation Model.

	PBE0-DH	mPW2PLYP	B2PLYP	PBE-QIDH	B2GPPLYP	PBE0-2	Exp
**1**	3.284	3.164	3.123	3.210	3.135	3.141	3.129
**2**	3.266	3.146	3.105	3.189	3.115	3.117	3.084
**3**	3.301	3.179	3.137	3.225	3.148	3.152	3.107
**4**	3.209	3.082	3.038	3.139	3.058	3.069	3.100
**5**	3.190	3.062	3.018	3.116	3.034	3.041	3.026
**6**	3.230	3.101	3.056	3.156	3.073	3.081	3.067
**7**	3.090	2.978	2.941	3.027	2.955	2.964	2.957
**8**	3.070	2.958	2.920	3.006	2.933	2.941	2.910
**9**	3.101	2.987	2.948	3.038	2.964	2.973	2.933
**10**	3.112	2.999	2.959	3.050	2.975	2.986	2.947
**11**	2.929	2.834	2.800	2.881	2.818	2.831	2.818
**12a**	3.057	2.930	2.881	2.999	2.909	2.930	2.890
**12b**	3.223	3.097	3.044	3.177	3.086	3.122	2.995
**13**	3.151	3.021	2.970	3.089	3.000	3.020	2.870
MAE	0.170	0.053	0.026	0.105	0.033	0.043	
MSE	0.170	0.050	0.008	0.105	0.026	0.038	
Max	0.281	0.151	0.100	0.219	0.130	0.150	
Min	0.109	-0.018	-0.062	0.039	-0.042	-0.031	
Δ_{err}	0.172	0.170	0.160	0.180	0.170	0.181	
SDAE	0.044	0.036	0.027	0.046	0.037	0.043	
R^2	0.822	0.841	0.860	0.793	0.826	0.779	

Table S37: Vertical Excitation Energies (eV) of the B Band (Soret) Using RSDH Density Functionals with the def2-TZVP Basis Set and CPCM solvation Model.

	RSX-0DH	ωB2PLYP	RSX-QIDH	ωB2GPPLYP	ωB88PP86	ωPBEPP86	Exp
**1**	3.436	3.305	3.317	3.268	3.197	3.168	3.129
**2**	3.419	3.287	3.297	3.248	3.175	3.145	3.084
**3**	3.462	3.328	3.337	3.287	3.212	3.181	3.107
**4**	3.385	3.243	3.258	3.204	3.125	3.094	3.100
**5**	3.366	3.221	3.236	3.181	3.098	3.066	3.026
**6**	3.414	3.267	3.279	3.224	3.140	3.107	3.067
**7**	3.249	3.123	3.136	3.087	3.020	2.992	2.957
**8**	3.232	3.105	3.116	3.068	2.999	2.971	2.910
**9**	3.272	3.143	3.153	3.104	3.032	3.003	2.933
**10**	3.273	3.143	3.158	3.108	3.038	3.011	2.947
**11**	3.067	2.962	2.972	2.932	2.879	2.857	2.818
**12a**	3.275	3.126	3.142	3.085	2.998	2.964	2.890
**12b**	3.454	3.306	3.326	3.269	3.176	3.146	2.995
**13**	3.375	3.225	3.236	3.181	3.087	3.052	2.870
MAE	0.346	0.211	0.224	0.172	0.096	0.067	
MSE	0.346	0.211	0.224	0.172	0.096	0.066	
Max	0.505	0.355	0.366	0.311	0.217	0.182	
Min	0.249	0.143	0.154	0.104	0.025	-0.006	
Δ_{err}	0.256	0.210	0.212	0.210	0.192	0.188	
SDAE	0.067	0.059	0.059	0.057	0.049	0.047	
R^2	0.643	0.692	0.686	0.702	0.761	0.770	

Table S38: Vertical Excitation Energies (eV) of the B Band (Soret) Using SCS/SOS-GDH Density Functionals with the def2-TZVP Basis Set and CPCM solvation Model.

	DSD-BLYP	DSD-PBEP86	SCS/SOS-B2PLYP	SCS-B2GPPLYP	SOS-B2GPPLYP	SCS-PBE-QIDH	SOS-PBE-QIDH	Exp
**1**	2.640	2.604	3.394	3.254	3.212	3.120	3.115	3.129
**2**	2.601	2.567	3.376	3.234	3.192	3.097	3.092	3.084
**3**	2.628	2.592	3.415	3.271	3.227	3.130	3.124	3.107
**4**	2.530	2.487	3.332	3.192	3.148	3.047	3.044	3.100
**5**	2.479	2.439	3.313	3.168	3.123	3.020	3.017	3.026
**6**	2.512	2.471	3.357	3.210	3.163	3.058	3.054	3.067
**7**	2.496	2.461	3.221	3.080	3.034	2.936	2.933	2.957
**8**	2.463	2.430	3.202	3.059	3.013	2.914	2.910	2.910
**9**	2.484	2.449	3.237	3.093	3.046	2.944	2.941	2.933
**10**	2.503	2.464	3.245	3.104	3.058	2.958	2.954	2.947
**11**	2.441	2.410	3.066	2.921	2.875	2.779	2.770	2.818
**12a**	2.340	2.298	3.183	3.032	2.982	2.879	2.869	2.890
**12b**	2.547	2.492	3.357	3.211	3.160	3.055	3.044	2.995
**13**	2.416	2.367	3.284	3.122	3.070	2.958	2.946	2.870
MAE	0.482	0.522	0.296	0.151	0.105	0.026	0.025	
MSE	-0.482	-0.522	0.296	0.151	0.105	0.004	-0.001	
Max	-0.377	-0.408	0.414	0.252	0.200	0.088	0.076	
Min	-0.570	-0.613	0.232	0.092	0.048	-0.053	-0.056	
Δ_{err}	0.190	0.210	0.182	0.160	0.152	0.141	0.132	
SDAE	0.055	0.057	0.046	0.041	0.039	0.025	0.023	
R^2	0.694	0.671	0.797	0.833	0.850	0.872	0.885	

Table S39: Vertical Excitation Energies (eV) of the B Band (Soret) Using SCS/SOS-RSDH Density Functionals with the def2-TZVP Basis Set and CPCM solvation Model.

	SCS/SOS-ωB2PLYP	SCS-ωB2GPPLYP	SOS-ωB2GPPLYP	SCS-RSX-QIDH	SOS-RSX-QIDH	SCS-ωB88PP86	SOS-ωB88PP86	SCS-ωPBEPP86	SOS-ωPBEPP86	Exp
**1**	3.188	3.102	3.154	2.970	3.023	3.150	3.134	2.994	3.078	3.129
**2**	3.205	3.079	3.132	2.942	2.997	3.127	3.112	2.967	3.054	3.084
**3**	3.243	3.112	3.168	2.971	3.029	3.161	3.146	2.997	3.087	3.107
**4**	3.163	3.029	3.090	2.875	2.951	3.078	3.066	2.904	3.007	3.100
**5**	3.138	2.999	3.061	2.841	2.919	3.049	3.037	2.871	2.977	3.026
**6**	3.180	3.037	3.102	2.874	2.955	3.088	3.076	2.904	3.014	3.067
**7**	3.041	2.906	2.970	2.748	2.834	2.960	2.950	2.780	2.891	2.957
**8**	3.021	2.884	2.950	2.723	2.811	2.938	2.928	2.756	2.869	2.910
**9**	3.058	2.916	2.984	2.749	2.842	2.970	2.961	2.782	2.900	2.933
**10**	3.061	2.925	2.990	2.765	2.853	2.979	2.969	2.798	2.911	2.947
**11**	2.862	2.734	2.791	2.603	2.649	2.795	2.779	2.625	2.719	2.818
**12a**	3.021	2.876	2.936	2.731	2.780	2.927	2.908	2.744	2.840	2.890
**12b**	3.197	3.058	3.116	2.915	2.956	3.104	3.081	2.923	3.015	2.995
**13**	3.108	2.956	3.018	2.804	2.849	3.007	2.986	2.817	2.914	2.870
MAE	0.118	0.038	0.050	0.166	0.099	0.042	0.033	0.141	0.049	
MSE	0.118	-0.016	0.045	-0.166	-0.099	0.036	0.021	-0.141	-0.040	
Max	0.238	0.086	0.148	-0.066	-0.021	0.137	0.116	-0.053	0.044	
Min	0.044	-0.084	-0.027	-0.225	-0.169	-0.023	-0.039	-0.196	-0.099	
Δ_{err}	0.194	0.170	0.175	0.159	0.148	0.160	0.155	0.143	0.143	
SDAE	0.052	0.028	0.039	0.047	0.038	0.037	0.032	0.041	0.024	
R^2	0.751	0.819	0.815	0.812	0.872	0.825	0.844	0.854	0.867	

S8 Comparison of Calculated and Experimnetal Excitaion Energies: The Q1 Band

Table S40: Vertical Excitation Energies (eV) of the Q1 Band Using GDH Density Functionals with the def2-TZVP Basis Set and CPCM solvation Model.

	PBE0-DH	mPW2PLYP	B2PLYP	PBE-QIDH	B2GPPLYP	PBE0-2	Exp
**1**	2.477	2.489	2.496	2.515	2.529	2.599	2.537
**2**	2.381	2.365	2.367	2.370	2.377	2.410	2.309
**3**	2.431	2.417	2.419	2.422	2.430	2.463	2.357
**4**	2.432	2.451	2.460	2.484	2.499	2.584	2.487
**5**	2.338	2.330	2.330	2.344	2.352	2.402	2.277
**6**	2.383	2.376	2.381	2.391	2.400	2.450	2.327
**7**	2.365	2.383	2.387	2.431	2.441	2.542	2.411
**8**	2.274	2.262	2.261	2.287	2.289	2.351	2.198
**9**	2.326	2.316	2.315	2.341	2.344	2.404	2.258
**10**	2.388	2.407	2.413	2.451	2.463	2.563	2.409
**11**	2.185	2.170	2.162	2.206	2.195	2.215	2.127
**12**	2.141	2.166	2.177	2.198	2.214	2.298	2.046
**13**	2.159	2.161	2.167	2.178	2.187	2.237	1.974
MAE	0.071	0.062	0.062	0.073	0.078	0.139	
MSE	0.043	0.044	0.048	0.069	0.077	0.139	
Max	0.185	0.187	0.193	0.204	0.213	0.263	
Min	-0.060	-0.048	-0.041	-0.022	-0.008	0.062	
Δ_{err}	0.245	0.235	0.230	0.226	0.220	0.201	
SDAE	0.038	0.046	0.049	0.054	0.057	0.059	
R^2	0.910	0.945	0.938	0.975	0.961	0.909	

Table S41: Vertical Excitation Energies (eV) of the Q1 Band Using RSDH Density Functionals with the def2-TZVP Basis Set and CPCM solvation Model.

	RSX-0DH	ωB2PLYP	RSX-QIDH	ωB2GPPLYP	ωB88PP86	ωPBEPP86	Exp
**1**	2.214	2.310	2.360	2.376	2.469	2.523	2.537
**2**	2.096	2.163	2.203	2.213	2.298	2.339	2.309
**3**	2.155	2.224	2.261	2.272	2.357	2.397	2.357
**4**	2.170	2.276	2.333	2.351	2.446	2.505	2.487
**5**	2.054	2.132	2.181	2.191	2.281	2.327	2.277
**6**	2.109	2.188	2.235	2.246	2.335	2.380	2.327
**7**	2.130	2.233	2.295	2.312	2.397	2.457	2.411
**8**	2.017	2.089	2.141	2.149	2.227	2.273	2.198
**9**	2.078	2.151	2.199	2.209	2.287	2.331	2.258
**10**	2.141	2.246	2.310	2.327	2.416	2.477	2.409
**11**	2.111	2.106	2.133	2.121	2.154	2.171	2.127
**12**	1.915	2.020	2.071	2.090	2.180	2.234	2.046
**13**	1.929	2.005	2.046	2.057	2.138	2.179	1.974
MAE	0.200	0.126	0.089	0.081	0.041	0.070	
MSE	-0.200	-0.121	-0.073	-0.062	0.021	0.067	
Max	-0.016	0.031	0.072	0.083	0.164	0.205	
Min	-0.323	-0.227	-0.177	-0.161	-0.068	-0.014	
Δ_{err}	0.307	0.258	0.249	0.240	0.232	0.219	
SDAE	0.093	0.067	0.047	0.040	0.052	0.059	
R^2	0.823	0.967	0.975	0.971	0.952	0.923	

Table S42: Vertical Excitation Energies (eV) of the Q1 Band Using SCS/SOS-GDH Density Functionals with the def2-TZVP Basis Set and CPCM solvation Model.

	DSD-BLYP	DSD-PBEP86	SCS/SOS-B2PLYP	SCS-B2GPPLYP	SOS-B2GPPLYP	SCS-PBE-QIDH	SOS-PBE-QIDH	Exp
**1**	2.997	2.969	2.699	2.454	2.387	2.275	2.200	2.537
**2**	2.670	2.650	2.578	2.284	2.211	2.083	1.997	2.309
**3**	2.729	2.710	2.628	2.335	2.262	2.135	2.050	2.357
**4**	3.023	2.991	2.672	2.432	2.363	2.247	2.176	2.487
**5**	2.711	2.688	2.553	2.267	2.192	2.062	1.980	2.277
**6**	2.763	2.741	2.598	2.315	2.239	2.110	2.029	2.327
**7**	2.963	2.917	2.618	2.381	2.308	2.187	2.115	2.411
**8**	2.624	2.589	2.500	2.212	2.132	1.995	1.912	2.198
**9**	2.681	2.648	2.553	2.264	2.185	2.048	1.966	2.258
**10**	2.999	2.956	2.639	2.397	2.323	2.203	2.129	2.409
**11**	2.299	2.296	2.442	2.213	2.141	2.000	1.948	2.127
**12**	2.729	2.700	2.387	2.176	2.112	2.002	1.944	2.046
**13**	2.541	2.523	2.388	2.135	2.065	1.939	1.872	1.974
MAE	0.462	0.435	0.272	0.050	0.088	0.187	0.261	
MSE	0.462	0.435	0.272	0.011	-0.061	-0.187	-0.261	
Max	0.590	0.549	0.414	0.161	0.091	-0.035	-0.102	
Min	0.172	0.169	0.162	-0.083	-0.150	-0.262	-0.337	
Δ_{err}	0.420	0.380	0.252	0.244	0.241	0.227	0.235	
SDAE	0.128	0.118	0.067	0.051	0.032	0.072	0.080	
R^2	0.787	0.797	0.976	0.958	0.944	0.915	0.864	

Table S43: Vertical Excitation Energies (eV) of the Q1 Band Using SCS/SOS-RSDH Density Functionals with the def2-TZVP Basis Set and CPCM solvation Model.

	SCS/SOS-ωB2PLYP	SCS-ωB2GPPLYP	SOS-ωB2GPPLYP	SCS-RSX-QIDH	SOS-RSX-QIDH	SCS-ωB88PP86	SOS-ωB88PP86	SCS-ωPBEPP86	SOS-ωPBEPP86	Exp
**1**	2.006	1.933	1.945	1.982	1.686	2.100	2.005	1.953	1.920	2.537
**2**	1.808	1.707	1.715	1.768	1.419	1.896	1.790	1.737	1.688	2.309
**3**	1.871	1.767	1.777	1.824	1.485	1.953	1.850	1.791	1.748	2.357
**4**	1.973	1.889	1.919	1.906	1.659	2.063	1.977	1.890	1.893	2.487
**5**	1.779	1.669	1.694	1.698	1.401	1.864	1.769	1.679	1.668	2.277
**6**	1.838	1.726	1.753	1.750	1.463	1.918	1.825	1.730	1.725	2.327
**7**	1.923	1.832	1.867	1.836	1.596	2.005	1.920	1.819	1.832	2.411
**8**	1.725	1.603	1.634	1.618	1.327	1.800	1.704	1.599	1.598	2.198
**9**	1.790	1.665	1.698	1.676	1.393	1.858	1.765	1.655	1.659	2.258
**10**	1.930	1.839	1.875	1.845	1.604	2.016	1.930	1.828	1.842	2.409
**11**	1.869	1.720	1.785	1.595	1.516	1.872	1.810	1.640	1.715	2.127
**12**	1.755	1.668	1.708	1.650	1.469	1.832	1.761	1.651	1.682	2.046
**13**	1.695	1.578	1.618	1.564	1.346	1.759	1.681	1.562	1.587	1.974
MAE	0.443	0.548	0.518	0.539	0.796	0.368	0.456	0.553	0.551	
MSE	-0.443	-0.548	-0.518	-0.539	-0.796	-0.368	-0.456	-0.553	-0.551	
Max	-0.258	-0.378	-0.338	-0.396	-0.577	-0.214	-0.285	-0.395	-0.364	
Min	-0.531	-0.608	-0.594	-0.582	-0.890	-0.437	-0.532	-0.603	-0.621	
Δ_{err}	0.273	0.230	0.256	0.186	0.313	0.223	0.247	0.208	0.257	
SDAE	0.097	0.089	0.100	0.063	0.112	0.081	0.091	0.073	0.095	
R^2	0.739	0.763	0.673	0.886	0.544	0.851	0.768	0.856	0.716	

S9 Comparison of Calculated and Experimnetal Excitaion Energies: The Q3 Band

Table S44: Vertical Excitation Energies (eV) of the Q3 Band Using GDH Density Functionals with the def2-TZVP Basis Set and CPCM solvation Model.

	PBE0-DH	mPW2PLYP	B2PLYP	PBE-QIDH	B2GPPLYP	PBE0-2	Exp
**1**	2.270	2.222	2.219	2.208	2.206	2.204	2.203
**4**	2.237	2.199	2.199	2.194	2.195	2.210	2.181
**7**	2.160	2.115	2.109	2.122	2.114	2.140	2.095
**10**	2.186	2.143	2.138	2.150	2.143	2.172	2.097
**11**	1.884	1.837	1.827	1.845	1.830	1.832	1.701
**12**	2.097	2.068	2.069	2.064	2.065	2.076	1.873
MAE	0.114	0.072	0.068	0.072	0.067	0.081	
MSE	0.114	0.072	0.068	0.072	0.067	0.081	
Max	0.224	0.195	0.196	0.191	0.192	0.203	
Min	0.056	0.018	0.014	0.005	0.003	0.001	
Δ_{err}	0.168	0.180	0.180	0.186	0.190	0.202	
SDAE	0.071	0.075	0.076	0.077	0.076	0.075	
R^2	0.939	0.917	0.907	0.927	0.914	0.909	

Table S45: Vertical Excitation Energies (eV) of the Q3 Band Using RSDH Density Functionals with the def2-TZVP Basis Set and CPCM solvation Model.

	RSX-0DH	ωB2PLYP	RSX-QIDH	ωB2GPPLYP	ωB88PP86	ωPBEPP86	Exp
**1**	1.943	1.982	2.018	2.019	2.104	2.133	2.203
**4**	1.912	1.964	2.009	2.011	2.100	2.136	2.181
**7**	1.864	1.906	1.955	1.952	2.030	2.065	2.095
**10**	1.882	1.928	1.979	1.977	2.059	2.095	2.097
**11**	1.677	1.680	1.716	1.703	1.753	1.771	1.701
**12**	1.819	1.863	1.901	1.902	1.982	2.012	1.873
MAE	0.176	0.138	0.110	0.108	0.074	0.059	
MSE	-0.176	-0.138	-0.095	-0.098	-0.020	0.010	
Max	-0.024	-0.010	0.028	0.029	0.109	0.139	
Min	-0.269	-0.221	-0.185	-0.184	-0.099	-0.070	
Δ_{err}	0.245	0.210	0.213	0.210	0.208	0.209	
SDAE	0.108	0.097	0.072	0.075	0.027	0.047	
R^2	0.941	0.920	0.927	0.917	0.905	0.902	

Table S46: Vertical Excitation Energies (eV) of the Q3 Band Using SCS/SOS-GDH Density Functionals with the def2-TZVP Basis Set and CPCM solvation Model.

	DSD-BLYP	DSD-PBEP86	SCS/SOS-B2PLYP	SCS-B2GPPLYP	SOS-B2GPPLYP	SCS-PBE-QIDH	SOS-PBE-QIDH	Exp
**1**	2.338	2.325	2.440	2.090	2.010	1.866	1.765	2.203
**4**	2.398	2.382	2.427	2.085	2.002	1.856	1.760	2.181
**7**	2.281	2.255	2.360	2.014	1.928	1.774	1.677	2.095
**10**	2.332	2.307	2.385	2.038	1.951	1.799	1.701	2.097
**11**	1.837	1.827	2.103	1.791	1.711	1.552	1.476	1.701
**12**	2.251	2.241	2.304	1.992	1.914	1.770	1.689	1.873
MAE	0.215	0.198	0.312	0.093	0.123	0.256	0.347	
MSE	0.215	0.198	0.312	-0.023	-0.106	-0.256	-0.347	
Max	0.378	0.368	0.431	0.119	0.041	-0.103	-0.184	
Min	0.135	0.122	0.237	-0.113	-0.193	-0.337	-0.438	
Δ_{err}	0.240	0.250	0.194	0.232	0.234	0.234	0.254	
SDAE	0.090	0.091	0.084	0.022	0.077	0.102	0.112	
R^2	0.809	0.801	0.931	0.882	0.869	0.849	0.810	

Table S47: Vertical Excitation Energies (eV) of the Q3 Band Using SCS/SOS-RSDH Density Functionals with the def2-TZVP Basis Set and CPCM solvation Model.

	SCS/SOS-ωB2PLYP	SCS-ωB2GPPLYP	SOS-ωB2GPPLYP	SCS-RSX-QIDH	SOS-RSX-QIDH	SCS-ωB88PP86	SOS-ωB88PP86	SCS-ωPBEPP86	SOS-ωPBEPP86	Exp
**1**	1.560	1.436	1.431	1.533	1.092	1.656	1.532	1.491	1.410	2.203
**4**	1.542	1.408	1.421	1.473	1.084	1.636	1.521	1.445	1.401	2.181
**7**	1.473	1.324	1.344	1.374	0.992	1.555	1.441	1.346	1.315	2.095
**10**	1.492	1.343	1.365	1.395	1.016	1.578	1.464	1.367	1.339	2.097
**11**	1.315	1.156	1.192	1.137	0.862	1.365	1.269	1.141	1.149	1.701
**12**	1.485	1.347	1.374	1.367	1.057	1.561	1.462	1.358	1.348	1.873
MAE	0.547	0.689	0.671	0.645	1.008	0.467	0.577	0.667	0.698	
MSE	-0.547	-0.689	-0.671	-0.645	-1.008	-0.467	-0.577	-0.667	-0.698	
Max	-0.386	-0.526	-0.499	-0.506	-0.816	-0.312	-0.411	-0.515	-0.525	
Min	-0.643	-0.773	-0.772	-0.721	-1.111	-0.547	-0.671	-0.749	-0.793	
Δ_{err}	0.257	0.247	0.273	0.215	0.295	0.235	0.260	0.234	0.268	
SDAE	0.125	0.119	0.130	0.089	0.140	0.111	0.121	0.102	0.124	
R^2	0.810	0.783	0.750	0.853	0.619	0.833	0.787	0.818	0.751	

S10 Summary of Statistical Measures Compared with Experiment

Table S48: The Mean Absolute Error (MAE), Mean Signed Error (MSE), Maximum Error (Max), Minimum Error (Min), Error Range (Δ_{err}), Standard Deviation of the Absolute Errors (SDAE), and squared Pearson correlation coefficient (R^2) of the Vertical Excitation Energies in eV of the B band with the def2-TZVP Basis Set of all Molecules **1**–**13**.

Functional	MAE	MSE	Max	Min	Δ_{err}	SDAE	R^2
**GDH**							
PBE0-DH	0.170	0.170	0.281	0.109	0.172	0.044	0.822
mPW2PLYP	0.053	0.050	0.151	-0.018	0.170	0.036	0.841
B2PLYP	0.026	0.008	0.100	-0.062	0.160	0.027	0.860
PBE-QIDH	0.105	0.105	0.219	0.039	0.180	0.046	0.793
B2GPPLYP	0.033	0.026	0.130	-0.042	0.170	0.037	0.826
PBE0-2	0.043	0.038	0.150	-0.031	0.181	0.043	0.779
**RSDH**							
RSX-0DH	0.346	0.346	0.505	0.249	0.256	0.067	0.643
ωB2PLYP	0.211	0.211	0.355	0.143	0.210	0.059	0.692
RSX-QIDH	0.224	0.224	0.366	0.154	0.212	0.059	0.686
ωB2GPPLYP	0.172	0.172	0.311	0.104	0.210	0.057	0.702
ωB88PP86	0.096	0.096	0.217	0.025	0.192	0.049	0.761
ωPBEPP86	0.067	0.066	0.182	-0.006	0.188	0.047	0.770
**SCS/SOS-GDH**							
DSD-BLYP	0.482	-0.482	-0.377	-0.570	0.190	0.055	0.694
DSD-PBEP86	0.522	-0.522	-0.408	-0.613	0.210	0.057	0.671
SCS/SOS-B2PLYP	0.296	0.296	0.414	0.232	0.182	0.046	0.797
SCS-B2GPPLYP	0.151	0.151	0.252	0.092	0.160	0.041	0.833
SOS-B2GPPLYP	0.105	0.105	0.200	0.048	0.152	0.039	0.850
SCS-PBE-QIDH	0.026	0.004	0.088	-0.053	0.141	0.025	0.872
SOS-PBE-QIDH	0.025	-0.001	0.076	-0.056	0.132	0.023	0.885
**SCS/SOS-RSDH**							
SCS/SOS-ωB2PLYP	0.118	0.118	0.238	0.044	0.194	0.052	0.751
SCS-ωB2GPPLYP	0.038	-0.016	0.086	-0.084	0.170	0.028	0.819
SOS-ωB2GPPLYP	0.050	0.045	0.148	-0.027	0.175	0.039	0.815
SCS-RSX-QIDH	0.166	-0.166	-0.066	-0.225	0.159	0.047	0.812
SOS-RSX-QIDH	0.099	-0.099	-0.021	-0.169	0.148	0.038	0.872
SCS-ωB88PP86	0.042	0.036	0.137	-0.023	0.160	0.037	0.825
SOS-ωB88PP86	0.033	0.021	0.116	-0.039	0.155	0.032	0.844
SCS-ωPBEPP86	0.141	-0.141	-0.053	-0.196	0.143	0.041	0.854
SOS-ωPBEPP86	0.049	-0.040	0.044	-0.099	0.143	0.024	0.867

Table S49: The Mean Absolute Error (MAE), Mean Signed Error (MSE), Maximum Error (Max), Minimum Error (Min), Error Range (Δ_{err}), Standard Deviation of the Absolute Errors (SDAE), and squared Pearson correlation coefficient (R^2) of the Vertical Excitation Energies in eV of the Q1 band with the def2-TZVP Basis Set of all Molecules **1–13**.

Functional	MAE	MSE	Max	Min	Δ_{err}	SDAE	R^2
**GDH**							
PBE0-DH	0.071	0.043	0.185	-0.060	0.245	0.038	0.910
mPW2PLYP	0.062	0.044	0.187	-0.048	0.235	0.046	0.945
B2PLYP	0.062	0.048	0.193	-0.041	0.230	0.049	0.938
PBE-QIDH	0.073	0.069	0.204	-0.022	0.226	0.054	0.975
B2GPPLYP	0.078	0.077	0.213	-0.008	0.220	0.057	0.961
PBE0-2	0.139	0.139	0.263	0.062	0.201	0.059	0.909
**RSDH**							
RSX-0DH	0.200	-0.200	-0.016	-0.323	0.307	0.093	0.823
ωB2PLYP	0.126	-0.121	0.031	-0.227	0.258	0.067	0.967
RSX-QIDH	0.089	-0.073	0.072	-0.177	0.249	0.047	0.975
ωB2GPPLYP	0.081	-0.062	0.083	-0.161	0.240	0.040	0.971
ωB88PP86	0.041	0.021	0.164	-0.068	0.232	0.052	0.952
ωPBEPP86	0.070	0.067	0.205	-0.014	0.219	0.059	0.923
**SCS/SOS-GDH**							
DSD-BLYP	0.462	0.462	0.590	0.172	0.420	0.128	0.787
DSD-PBEP86	0.435	0.435	0.549	0.169	0.380	0.118	0.797
SCS/SOS-B2PLYP	0.272	0.272	0.414	0.162	0.252	0.067	0.976
SCS-B2GPPLYP	0.050	0.011	0.161	-0.083	0.244	0.051	0.958
SOS-B2GPPLYP	0.088	-0.061	0.091	-0.150	0.241	0.032	0.944
SCS-PBE-QIDH	0.187	-0.187	-0.035	-0.262	0.227	0.072	0.915
SOS-PBE-QIDH	0.261	-0.261	-0.102	-0.337	0.235	0.080	0.864
**SCS/SOS-RSDH**							
SCS/SOS-ωB2PLYP	0.443	-0.443	-0.258	-0.531	0.273	0.097	0.739
SCS-ωB2GPPLYP	0.548	-0.548	-0.378	-0.608	0.230	0.089	0.763
SOS-ωB2GPPLYP	0.518	-0.518	-0.338	-0.594	0.256	0.100	0.673
SCS-RSX-QIDH	0.539	-0.539	-0.396	-0.582	0.186	0.063	0.886
SOS-RSX-QIDH	0.796	-0.796	-0.577	-0.890	0.313	0.112	0.544
SCS-ωB88PP86	0.368	-0.368	-0.214	-0.437	0.223	0.081	0.851
SOS-ωB88PP86	0.456	-0.456	-0.285	-0.532	0.247	0.091	0.768
SCS-ωPBEPP86	0.553	-0.553	-0.395	-0.603	0.208	0.073	0.856
SOS-ωPBEPP86	0.551	-0.551	-0.364	-0.621	0.257	0.095	0.716

Table S50: The Mean Absolute Error (MAE), Mean Signed Error (MSE), Maximum Error (Max), Minimum Error (Min), Error Range (Δ_{err}), Standard Deviation of the Absolute Errors (SDAE), and squared Pearson correlation coefficient (R^2) of the Vertical Excitation Energies in eV of the Q3 band with the def2-TZVP Basis Set of Molecules **1**, **4**, **7**, **10**, **11**, and **12**.

Functional	MAE	MSE	Max	Min	Δ_{err}	SDAE	R^2
**GDH**							
PBE0-DH	0.114	0.114	0.224	0.056	0.168	0.071	0.939
mPW2PLYP	0.072	0.072	0.195	0.018	0.180	0.075	0.917
B2PLYP	0.068	0.068	0.196	0.014	0.180	0.076	0.907
PBE-QIDH	0.072	0.072	0.191	0.005	0.186	0.077	0.927
B2GPPLYP	0.067	0.067	0.192	0.003	0.190	0.076	0.914
PBE0-2	0.081	0.081	0.203	0.001	0.202	0.075	0.909
**RSDH**							
RSX-0DH	0.176	-0.176	-0.024	-0.269	0.245	0.108	0.941
ωB2PLYP	0.138	-0.138	-0.010	-0.221	0.210	0.097	0.920
RSX-QIDH	0.110	-0.095	0.028	-0.185	0.213	0.072	0.927
ωB2GPPLYP	0.108	-0.098	0.029	-0.184	0.210	0.075	0.917
ωB88PP86	0.074	-0.020	0.109	-0.099	0.208	0.027	0.905
ωPBEPP86	0.059	0.010	0.139	-0.070	0.209	0.047	0.902
**SCS/SOS-GDH**							
DSD-BLYP	0.215	0.215	0.378	0.135	0.240	0.090	0.809
DSD-PBEP86	0.198	0.198	0.368	0.122	0.250	0.091	0.801
SCS/SOS-B2PLYP	0.312	0.312	0.431	0.237	0.194	0.084	0.931
SCS-B2GPPLYP	0.093	-0.023	0.119	-0.113	0.232	0.022	0.882
SOS-B2GPPLYP	0.123	-0.106	0.041	-0.193	0.234	0.077	0.869
SCS-PBE-QIDH	0.256	-0.256	-0.103	-0.337	0.234	0.102	0.849
SOS-PBE-QIDH	0.347	-0.347	-0.184	-0.438	0.254	0.112	0.810
**SCS/SOS-RSDH**							
SCS/SOS-ωB2PLYP	0.547	-0.547	-0.386	-0.643	0.257	0.125	0.810
SCS-ωB2GPPLYP	0.689	-0.689	-0.526	-0.773	0.247	0.119	0.783
SOS-ωB2GPPLYP	0.671	-0.671	-0.499	-0.772	0.273	0.130	0.750
SCS-RSX-QIDH	0.645	-0.645	-0.506	-0.721	0.215	0.089	0.853
SOS-RSX-QIDH	1.008	-1.008	-0.816	-1.111	0.295	0.140	0.619
SCS-ωB88PP86	0.467	-0.467	-0.312	-0.547	0.235	0.111	0.833
SOS-ωB88PP86	0.577	-0.577	-0.411	-0.671	0.260	0.121	0.787
SCS-ωPBEPP86	0.667	-0.667	-0.515	-0.749	0.234	0.102	0.818
SOS-ωPBEPP86	0.698	-0.698	-0.525	-0.793	0.268	0.124	0.751

S11 Leave-One-molecule-Out (LOO) Sensitivity Analysis

Table S51: Leave-one-molecule-out (LOO) sensitivity analysis of the B-band for the full set of DH functionals. MAE_{Full} is denoted by MAE while MAE_{LOO} is denoted by LOO for simplicity. The MAE obtained using all porphyrin transitions (N = 14), MAE and LOO values values are given in eV.

Functional	MAE	LOO min	LOO max	LOO span	MAE rank	Best LOO rank	Worst LOO rank
PBE0-DH	0.170	0.161	0.175	0.013	21	20	21
mPW2PLYP	0.053	0.045	0.056	0.010	11	10	11
B2PLYP	0.026	0.021	0.028	0.007	3	1	3
PBE-QIDH	0.105	0.096	0.110	0.014	15	14	16
B2GPPLYP	0.033	0.025	0.035	0.010	4	4	6
PBE0-2	0.043	0.034	0.045	0.011	8	6	8
RSX-0DH	0.346	0.334	0.354	0.020	26	26	26
ωB2PLYP	0.211	0.200	0.216	0.016	23	23	23
RSX-QIDH	0.224	0.212	0.229	0.017	24	24	24
ωB2GPPLYP	0.172	0.162	0.178	0.016	22	20	22
ωB88PP86	0.096	0.087	0.101	0.015	13	13	14
ωPBEPP86	0.067	0.058	0.072	0.014	12	12	12
DSD-BLYP	0.482	0.476	0.490	0.015	27	27	27
DSD-PBEP86	0.522	0.515	0.530	0.016	28	28	28
SCS/SOS-B2PLYP	0.296	0.287	0.301	0.014	25	25	25
SCS-B2GPPLYP	0.151	0.144	0.156	0.012	19	18	19
SOS-B2GPPLYP	0.105	0.098	0.109	0.012	16	15	16
SCS-PBE-QIDH	0.026	0.021	0.027	0.006	2	2	3
SOS-PBE-QIDH	0.025	0.021	0.027	0.006	1	1	3
SCS/SOS-ωB2PLYP	0.118	0.109	0.124	0.015	17	17	17
SCS-ωB2GPPLYP	0.038	0.034	0.040	0.006	6	5	8
SOS-ωB2GPPLYP	0.050	0.043	0.053	0.011	10	9	10
SCS-RSX-QIDH	0.166	0.161	0.174	0.012	20	20	22
SOS-RSX-QIDH	0.099	0.094	0.105	0.011	14	13	16
SCS-ωB88PP86	0.042	0.035	0.045	0.010	7	7	8
SOS-ωB88PP86	0.033	0.026	0.035	0.009	5	4	5
SCS-ωPBEPP86	0.141	0.137	0.148	0.011	18	18	19
SOS-ωPBEPP86	0.049	0.045	0.051	0.006	9	9	11

Table S52: Leave-one-molecule-out (LOO) sensitivity analysis of the Q1-band for the full set of DH functionals. MAE_{Full} is denoted by MAE while MAE_{LOO} is denoted by LOO for simplicity. The MAE obtained using all porphyrin molecules (N = 13), MAE and LOO values values are given in eV.

Functional	MAE	LOO min	LOO max	LOO span	MAE rank	Best LOO rank	Worst LOO rank
PBE0-DH	0.071	0.062	0.076	0.014	6	5	7
mPW2PLYP	0.062	0.051	0.067	0.015	4	3	4
B2PLYP	0.062	0.051	0.067	0.016	3	3	4
PBE-QIDH	0.073	0.062	0.079	0.017	7	6	8
B2GPPLYP	0.078	0.067	0.084	0.017	8	8	11
PBE0-2	0.139	0.128	0.145	0.017	13	12	13
RSX-0DH	0.200	0.190	0.215	0.026	15	15	15
ωB2PLYP	0.126	0.117	0.135	0.017	12	12	13
RSX-QIDH	0.089	0.082	0.096	0.014	11	9	11
ωB2GPPLYP	0.081	0.075	0.088	0.013	9	7	9
ωB88PP86	0.041	0.031	0.045	0.014	1	1	1
ωPBEPP86	0.070	0.058	0.074	0.016	5	5	6
DSD-BLYP	0.462	0.444	0.487	0.043	22	20	22
DSD-PBEP86	0.435	0.417	0.458	0.040	19	19	20
SCS/SOS-B2PLYP	0.272	0.260	0.281	0.021	17	16	17
SCS-B2GPPLYP	0.050	0.040	0.053	0.013	2	2	2
SOS-B2GPPLYP	0.088	0.082	0.094	0.011	10	10	11
SCS-PBE-QIDH	0.187	0.181	0.200	0.019	14	14	14
SOS-PBE-QIDH	0.261	0.255	0.275	0.020	16	16	17
SCS/SOS-ωB2PLYP	0.443	0.435	0.458	0.023	20	19	21
SCS-ωB2GPPLYP	0.548	0.543	0.562	0.019	25	25	26
SOS-ωB2GPPLYP	0.518	0.511	0.533	0.021	23	23	23
SCS-RSX-QIDH	0.539	0.535	0.551	0.016	24	24	24
SOS-RSX-QIDH	0.796	0.789	0.815	0.026	28	28	28
SCS-ωB88PP86	0.368	0.362	0.381	0.019	18	18	18
SOS-ωB88PP86	0.456	0.450	0.470	0.021	21	21	22
SCS-ωPBEPP86	0.553	0.548	0.566	0.017	27	25	27
SOS-ωPBEPP86	0.551	0.545	0.566	0.021	26	26	27

Table S53: Leave-one-molecule-out (LOO) sensitivity analysis of the Q3-band for the full set of DH functionals. MAE_{Full} is denoted by MAE while MAE_{LOO} is denoted by LOO for simplicity. The MAE obtained using all six free-base porphyrins ($N = 6$), MAE and LOO values values are given in eV.

Functional	MAE	LOO omit 1	LOO omit 4	LOO omit 7	LOO omit 10	LOO omit 11	LOO omit 12	LOO min	LOO max	LOO span	MAE rank	Best LOO rank	Worst LOO rank
PBE0-DH	0.114	0.123	0.126	0.124	0.119	0.100	0.092	0.092	0.126	0.034	11	9	13
mPW2PLYP	0.072	0.083	0.083	0.083	0.078	0.060	0.048	0.048	0.083	0.035	5	4	6
B2PLYP	0.068	0.079	0.079	0.079	0.074	0.057	0.043	0.043	0.079	0.036	3	2	4
PBE-QIDH	0.072	0.086	0.084	0.081	0.076	0.058	0.048	0.048	0.086	0.037	4	4	6
B2GPPLYP	0.067	0.080	0.078	0.077	0.071	0.055	0.042	0.042	0.080	0.038	2	1	4
PBE0-2	0.081	0.097	0.091	0.088	0.082	0.071	0.056	0.056	0.097	0.040	7	6	10
RSX-0DH	0.176	0.159	0.157	0.164	0.168	0.206	0.200	0.157	0.206	0.049	14	14	16
ωB2PLYP	0.138	0.121	0.122	0.128	0.132	0.161	0.163	0.121	0.163	0.042	13	12	13
RSX-QIDH	0.110	0.095	0.097	0.104	0.108	0.129	0.126	0.095	0.129	0.034	10	9	11
ωB2GPPLYP	0.108	0.093	0.096	0.101	0.106	0.129	0.124	0.093	0.129	0.036	9	8	11
ωB88PP86	0.074	0.069	0.073	0.076	0.081	0.078	0.067	0.067	0.081	0.014	6	2	7
ωPBEPP86	0.059	0.057	0.062	0.065	0.071	0.057	0.043	0.043	0.071	0.027	1	1	3
DSD-BLYP	0.215	0.230	0.214	0.220	0.210	0.230	0.182	0.182	0.230	0.049	16	15	16
DSD-PBEP86	0.198	0.213	0.197	0.205	0.195	0.212	0.164	0.164	0.213	0.049	15	14	15
SCS/SOS-B2PLYP	0.312	0.326	0.325	0.321	0.316	0.293	0.288	0.288	0.326	0.039	18	18	18
SCS-B2GPPLYP	0.093	0.089	0.092	0.095	0.100	0.094	0.088	0.088	0.100	0.012	8	7	8
SOS-B2GPPLYP	0.123	0.109	0.111	0.114	0.118	0.145	0.139	0.109	0.145	0.037	12	11	12
SCS-PBE-QIDH	0.256	0.239	0.242	0.242	0.247	0.277	0.286	0.239	0.286	0.047	17	17	17
SOS-PBE-QIDH	0.347	0.329	0.332	0.333	0.337	0.371	0.380	0.329	0.380	0.051	19	19	19
SCS/SOS-ωB2PLYP	0.547	0.528	0.529	0.532	0.536	0.579	0.579	0.528	0.579	0.051	21	21	21
SCS-ωB2GPPLYP	0.689	0.674	0.673	0.673	0.676	0.718	0.722	0.673	0.722	0.049	26	26	26
SOS-ωB2GPPLYP	0.671	0.650	0.653	0.654	0.658	0.703	0.705	0.650	0.705	0.055	25	24	25
SCS-RSX-QIDH	0.645	0.640	0.633	0.630	0.634	0.661	0.673	0.630	0.673	0.043	23	23	23
SOS-RSX-QIDH	1.008	0.987	0.990	0.989	0.993	1.042	1.046	0.987	1.046	0.059	28	28	28
SCS-ωB88PP86	0.467	0.450	0.451	0.452	0.456	0.493	0.497	0.450	0.497	0.047	20	20	20
SOS-ωB88PP86	0.577	0.558	0.560	0.561	0.566	0.606	0.610	0.558	0.610	0.052	22	22	22
SCS-ωPBEPP86	0.667	0.658	0.653	0.651	0.654	0.688	0.697	0.651	0.697	0.047	24	24	25
SOS-ωPBEPP86	0.698	0.679	0.682	0.682	0.686	0.727	0.733	0.679	0.733	0.054	27	27	27

S12 Computational Cost of DH Functionals

Table S54: Computation times (in minutes) for TD-DFT excited-state calculations, with def2-TZVP basis set, of molecule **1** (38 atoms, 828 basis functions), using various DH functionals. All calculations were performed using a CPU with 8 cores in parallel, 4.3 GHz clock speed and 64 GB of RAM.

Functional	Time/minute
PBE0-DH	21.6
mPW2PLYP	22.1
B2PLYP	22.1
PBE-QIDH	22.9
B2GPPLYP	22.2
PBE0-2	22.8
RSX-0DH	25.6
ωB2PLYP	25.4
RSX-QIDH	25.5
ωB2GPPLYP	25.6
ωB88PP86	25.5
ωPBEPP86	25.8
DSD-BLYP	22.6
DSD-PBEP86	22.6
SCS/SOS-B2PLYP	22.9
SCS-B2GPPLYP	23.1
SOS-B2GPPLYP	23.1
SCS-PBE-QIDH	23.5
SOS-PBE-QIDH	23.7
SCS/SOS-ωB2PLYP	26.7
SCS-ωB2GPPLYP	26.4
SOS-ωB2GPPLYP	26.4
SCS-RSX-QIDH	26.3
SOS-RSX-QIDH	26.3
SCS-ωB88PP86	26.3
SOS-ωB88PP86	26.5
SCS-ωPBEPP86	26.5
SOS-ωPBEPP86	26.3
